# Synthesis of Nanometer Sized Bis- and Tris-trityl Model Compounds with Different Extent of Spin–Spin Coupling

**DOI:** 10.3390/molecules23030682

**Published:** 2018-03-17

**Authors:** J. Jacques Jassoy, Andreas Meyer, Sebastian Spicher, Christine Wuebben, Olav Schiemann

**Affiliations:** Institute of Physical and Theoretical Chemistry, University of Bonn, 53115 Bonn, Germany; jassoy@pc.uni-bonn.de (J.J.J.); ameyer1@uni-bonn.de (A.M.); s6sespic@uni-bonn.de (S.S.); wuebben@thch.uni-bonn.de (C.W.)

**Keywords:** radicals, para-phenylene ethynylene, EPR, exchange coupling, pulsed dipolar spectroscopy, PELDOR, DEER

## Abstract

Tris(2,3,5,6-tetrathiaaryl)methyl radicals, so-called trityl radicals, are emerging as spin labels for distance measurements in biological systems based on Electron Paramagnetic Resonance (EPR). Here, the synthesis and characterization of rigid model systems carrying either two or three trityl moieties is reported. The monofunctionalized trityl radicals are connected to the molecular bridging scaffold via an esterification reaction employing the Mukaiyama reagent 2-chloro-methylpyridinium iodide. The bis- and tris-trityl compounds exhibit different inter-spin distances, strength of electron–electron exchange and dipolar coupling and can give rise to multi-spin effects. They are to serve as benchmark systems in comparing EPR distance measurement methods.

## 1. Introduction

### 1.1. Motivation

**Figure 1:**
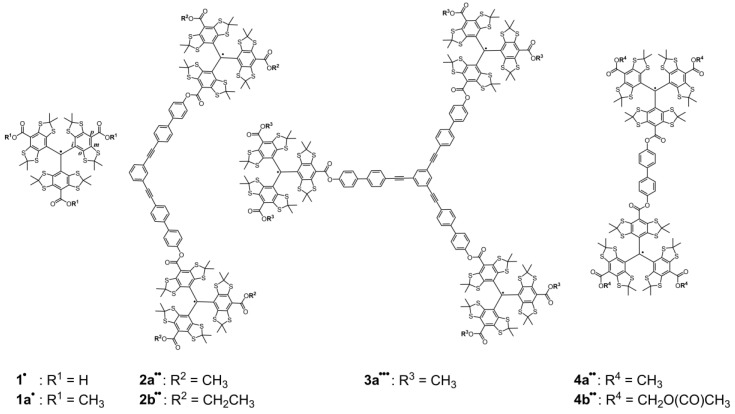
Lewis structures of the Finland Trityl **1^•^**, its methyl ester derivative **1a^•^** and of the five model compounds **2a^••^**, **2b^••^**, **3a^•••^**, **4a^••^** and **4b^••^**. The *ipso*, *ortho*, *meta* and *para* positions in the phenyl rings are indicated in molecule **1^•^** by the letters *i*, *o*, *m* and *p*, respectively.

The growing interest in EPR-based distance measurements [1,2,3,4,5] as a complementary method for elucidating the structure and dynamics of proteins [6,7,8,9,10,11], oligonucleotides [12,13,14,15,16,17] and their complexes [18,19] calls for the development of new spin labels [20,21,22,23,24,25,26,27,28,29,30] that broaden the scope of applications. In contrast to the conventionally used nitroxides [31], carbon-centered tris(2,3,5,6-tetrathiaaryl)methyl radicals [32], the so-called trityl radicals, display a higher persistence against the reducing environment in cells [33], a narrow EPR spectral width, 2 G vs. 70 G at X-band in the frozen state, and longer relaxation times T_1_ and T_2_ at room temperature [34,35]. These properties make trityl radicals promising candidates for single frequency pulsed EPR distance measurements such as Relaxation Induced Dipolar Modulation Enhancement (RIDME) [36,37,38], Double Quantum Coherence (DQC) [39,40,41], and Single-Frequency Technique for Refocusing dipolar couplings (SIFTER) [42] as well as for measurements at room temperature [43,44,45] and within cells [33]. However, although significant advancements have been achieved since the Nycomed Group patented the so-called Finland Trityl **1^•^** in 1996 (Figure 1) [46], the synthesis and functionalization of trityl radicals remains a challenging field in need of further improvements. Recently, it was demonstrated that the Mukaiyama esterification reagent 2-chloro-methyl-pyridinium iodide [47] leads to trityl esters under mild conditions [33]. Here, the Mukaiyama esterification reaction is used to synthesize methyl- and ethyl diester derivatives of Finland Trityl **1^•^**, which are then again esterified onto rigid molecular bridging scaffolds [48,49,50,51]. The obtained bis- and tris-trityl molecules **2a^••^**, **2b^••^**, **3a^•••^**, **4a^••^** and **4b^••^** (Figure 1) are interesting as model systems for EPR-based distance measurements for the following reasons: (1) Their line width and spin–spin distances may fall into a regime that could enable distance measurements via both cw and pulsed EPR methods, making a direct comparison of both approaches possible. (2) Compound **2a^••^** is the geometric parent to **3a^•••^** with the latter containing three instead of two trityl spin centers, which should enable the study of multispin effects under cw and pulsed EPR conditions [52]. (3) Compound **4a^••^** has a short trityl–trityl distance and may thus give rise to an isotropic exchange interaction, which would lead to the question into which coupling regime it belongs. (4) The different ester groups in the periphery (methyl, ethyl and acetylmethoxy esters) enable studying the effect of remote substitutions. Here, we report on the synthesis of these compounds and the cw EPR study.

### 1.2. Electron–Electron Interaction in Different Coupling Regimes

The starting point for discussing the interaction between electron spins is the spin Hamiltonian $H_{s}$. If isotropic *g*-values and hyperfine coupling to nearby nuclei are considered, the spin Hamiltonian operator in frequency units for a two-spin system has the following form [53]:

(1) $$H_{s}=\frac{\mu_{B}B_{0}}{h}(g_{1}S_{1,z}+g_{2}S_{2,z})+S_{1,z}\underset{Nuc}{\sum }a_{Nuc,1}I_{z,Nuc,1}+S_{2,z}\underset{Nuc}{\sum }a_{Nuc,2}I_{z,Nuc,2}+H_{ee}$$

In Equation (1), the first four terms refer only to one of the two electron spins (subscripts 1 or 2) and can therefore be classified as single-spin terms while the last term $H_{ee}$ sums up all electron–electron interactions. The single-spin terms can be used to calculate the transitions or resonance frequencies $\omega_{i}$ of either electron spin. To assign the coupling regime of two spins, the difference of their resonance frequencies $\Delta \omega =\omega_{1}-\omega_{2}$ is of importance. Inspection of Equation (1) reveals that Δω originates from differing *g*-values or hyperfine coupling interactions for the two electrons.

The two-electron operator $H_{ee}$ consists of two relevant terms accounting for isotropic exchange $H_{exc}$ and dipolar spin–spin coupling $H_{dip}$:

(2) $$H_{ee}=H_{exc}+H_{dip}=J\vec{S_{1}}\;\vec{S_{2}}+\vec{S_{1}}\overset{=}{D}\vec{S_{2}}$$

The isotropic exchange operator $H_{exc}$ contains the isotropic exchange coupling constant *J* as sole parameter and the spin operators ${\vec{S}}_{1}{\vec{S}}_{2}$. In the definition given in Equation (2), an exchange coupling constant *J* > 0 means that the two spins couple antiferromagnetically leading to a singlet ground state, whereas a negative value for *J* corresponds to a triplet ground state. If |*J*| >> *kT* holds, only the ground state is populated, which means that the molecule is diamagnetic, if *J* > 0. In the case presented below, |*J*| << *kT* holds for all temperatures used experimentally, meaning that both states are populated according to Boltzmann statistics. To explore how the exchange coupling in this case affects the resulting EPR spectrum, the operators in $H_{exc}$ can be extended to yield Equation (3) [54]:

(3) $$H_{exc}=J\vec{S_{1}}\;\vec{S_{2}}=JS_{1,z}S_{2,z}+\frac{1}{2}J\left(S_{1,+}S_{2,-}+S_{1,-}S_{2,+}\right)$$

The two terms in Equation (3) are called secular and pseudo-secular exchange, the latter of which leads to admixture of the spin wave functions in a diradical. The appearance of the cw EPR spectra in liquid solution is strongly affected by the occurrence of isotropic exchange. If |*J|* << |Δ*ω*|, the weak coupling regime is encountered, where the pseudo-secular component is negligible and can be omitted. Then, the system behaves like a typical diradical giving rise to two EPR signals with the *g*-values and hyperfine coupling constants (HFCC) of the individual spin centers. However, each observed EPR line will be shifted by *J*/2 due to the secular part of the exchange interaction. If |*J|* >> |Δ*ω*|, the molecule is in the strong coupling regime and can no longer be described as a diradical but rather as a molecule with a delocalized triplet ground state (or singlet ground state, depending on the sign of *J*). In this regime, only one EPR signal with a *g*-value corresponding to the average of the *g*-values of the individual spin centers and HFCCs reduced by a factor of 0.5. In between the strong and weak coupling regimes lies the intermediate coupling regime. In this regime, the number and position of the EPR lines depend in a complex manner on the ratio of *J* and Δ*ω.* Diagonalization of the spin Hamiltonian is necessary for a quantitative treatment of this coupling regime. The discussion of the coupling regimes is of pronounced interest for molecules, which contain a single ^13^C nucleus. For the corresponding isotopomers, the ^13^C nucleus is located on one of the two trityl groups (trityl **A**) and carries one of the two electron spins. In absence of exchange coupling, the transitions of this spin give rise to the satellite lines, whereas the transitions of spin 2 centered on trityl **B** contribute to the central line, which primarily originates from spin transitions of molecules that do not contain any ^13^C nucleus. Thus, Δ*ω* for these two spins is related to the hyperfine coupling constant of the ^13^C atoms as described by the relation $\Delta \omega =\frac{a_{iso}\left({}^{13}C\right)}{2}$.

**Figure 2:**
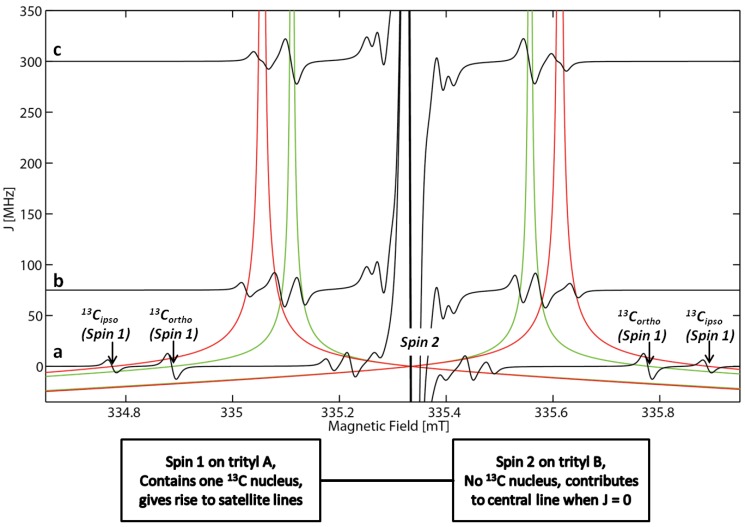
Simulated cw EPR spectra of a bis-trityl compound using typical parameters. The satellite lines stemming from the *i*- and *o*-^13^C nuclei are indicated by arrows and have hyperfine coupling constants of 31.25 and 25.00 MHz, respectively. The central line stems from electron spins centered on trityl groups that do not contain any ^13^C nuclei. The central line also includes the isotopomers that do not contain any ^13^C nuclei on either trityl group. The green and red lines indicate how the resonance fields of these satellite lines shift in dependence of the *J* value used in the simulation (spectrum **a**: *J* = 0 MHz; spectrum **b**: *J* = 75 MHz; spectrum **c**: *J* = 300 MHz). The resonance fields of the other satellites have been omitted for clarity.

If exchange coupling occurs, the spin centered on trityl **A** attains some of the character of the spin centered on trityl **B** and vice versa. Therefore, the effective isotropic coupling of the spin on trityl **A** is reduced. Furthermore, the spin centered on trityl **B** begins to show isotropic hyperfine coupling, even though the Lewis formula given in Figure 1 and the schematic drawing in Figure 2 imply that this spin is entirely centered on a trityl group, which does not contain any ^13^C nuclei. In the limit of strong exchange, i.e., |*J|*/|Δ*ω*| >> 1, the two spins become indistinguishable and give rise to transitions that appear as satellites with an apparent isotropic coupling that is reduced by 50% (a more theoretical discussion of this is found in the Supplementary Materials). Figure 2 illustrates the described behavior for two sets of satellite lines in dependence of the magnitude of the isotropic exchange coupling constant *J*.

Above, three coupling regimes have been distinguished (strong, intermediate and weak coupling) but the conditions for the occurrence of each regime have not been specified. A convenient choice seems to be |*J|*/|Δ*ω*| ≥ 10 for the occurrence of strong coupling and |*J|*/|Δ*ω*| ≤ 0.1 for weak coupling. This choice agrees with Figure 2, where two satellite lines originating from hyperfine interaction to the *i*- and *o*-^13^C nuclei occur on each side of the central line in the strong coupling regime (*J* = 300 MHz, label **c** in Figure 2) at half the separation expected for the absence of exchange coupling. In the intermediate coupling regime (*J* = 75 MHz, label **b** in Figure 2), four allowed transitions are expected. The simulated spectrum clearly differs from the simulation in the strong coupling regime. In the special case given in Figure 2, the position of the lines is already similar to the position expected for strong coupling. Furthermore, two of these transitions are accidentally almost degenerate, which reduces the number of EPR lines to three.

For distance measurements, particularly the dipolar spin–spin interaction introduced by the dipolar Hamiltonian $H_{dip}$ is of interest. $H_{dip}$ contains the spin operators $\vec{S_{i}}$ of the interacting spins and the dipolar interaction matrix $\overset{=}{D}$. In the case presented below, the Zeeman interaction strongly exceeds all other terms in the spin Hamiltonian, and the operator $H_{dip}$ can be written in analogy to $H_{exc}$ [55]:

(4) $$H_{dip}={\vec{S}}_{1}\overset{=}{D}{\overset{\to }{S}}_{2}=D\left(1-3cos^{2}\theta \right)(S_{1,z}S_{2,z}-\frac{1}{4}\left(S_{1,+}S_{2,-}+S_{1,-}S_{2,+})\right)$$

The first term in Equation (4) corresponds to the secular interaction, and the second to the pseudo-secular interaction. In the absence of an external magnetic field or if the external magnetic field is very weak, further terms are required [55]. D is the dipolar coupling constant, which depends on the inter-spin distance *r*. In the weak coupling regime, *r* can be calculated using Equation (5):

(5) $$D=52.01\;\mathrm{MHz}\cdot \mathrm{nm}{}^{3}/r^{3}$$

Note, that *r* in Equation (5) has to be considered an effective electron–electron separation. Due to electron delocalization into the phenyl rings, *r* is not necessarily identical to the separation of the central carbon atoms in the trityls. However, the spin density on these phenyl rings is rather low (~9% on each ring [29]). Furthermore, the three rings are arranged symmetrically around the central carbon atom of the trityl and therefore the effects of delocalization are partially cancelled out. In the examples presented below, it was not necessary to account for electron delocalization. The borders of the different coupling regimes can be defined in analogy to the case of pure exchange coupling as described above if the ratio of |*J* − *D*/2|/|Δ*ω*| is considered instead of |*J|*/|Δ*ω*|. If |*J* − *D*/2|/|Δ*ω*| ≤ 0.1, weak coupling occurs. In this case, the transition frequencies are affected by *J* and *D* and the resulting EPR spectra have the shape of a Pake pattern, in which the two halves of the spectrum are shifted against each other by half the isotropic exchange coupling constant *J*/2. For the perpendicular orientation, the shift of the resonance frequency is given by [56,57]:

(6) $$\upsilon_{┴}=\left|D\pm J\right|/2$$

where the plus sign is valid if the exchange coupling constant *J* is positive. If the inter-spin vector is parallel to the external field, $\upsilon_{\|}$ is obtained:

(7) $$\upsilon_{\|}=\left|-2D\pm J\right|/2$$

Thus, in the weak coupling regime, it is possible to extract *D*, *J*, and their relative signs from the frequency spectra using Equations (5) and (6). In the strong coupling regime (i.e., |*J* − *D*/2|/|Δ*ω*| ≥ 10), the frequency spectra have the shape of an apparently undistorted Pake pattern, meaning that the two halves of the spectrum are not shifted by the exchange coupling constant and the frequencies of the singularities can be used directly to calculate *r*. However, the singularities occur at frequency values 50% larger than expected for a given distance. Thus, Equation (5) has to be modified in the strong coupling regime to yield Equation (8):

(8) $$D_{strong}=1.5\cdot 52.01\;\mathrm{MHz}\cdot \mathrm{nm}{}^{3}/r^{3}$$

This yields Equations (9) and (10) [2]:

(9) $$\upsilon_{┴}=\left|1.5\cdot D\right|/2$$

(10) $$\upsilon_{\|}=\left|-3\cdot D\right|/2$$

A Pake pattern stretched by a factor of 1.5 leads to a distance distribution with peaks at values of roughly 87% of the actual inter-spin distances. Finally, the most complicated case is encountered in the intermediate coupling regime. There, the expected frequencies lie in between the extreme situations described by Equations (6)–(10) and the spectrum can only be simulated by diagonalization of the complete spin Hamiltonian matrix.

## 2. Results and Discussion

### 2.1. Syntheses

**Scheme 1:**
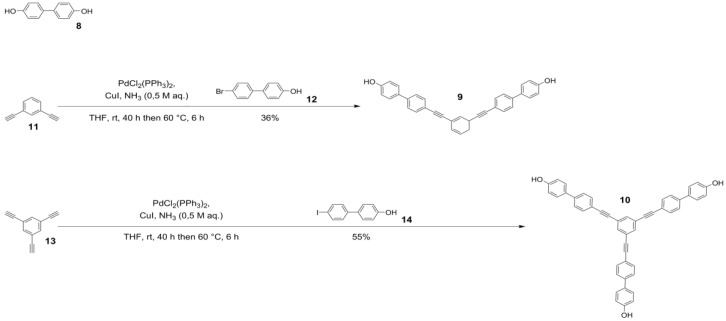
Bridging compound **8** and synthesis scheme for bridging compounds **9** and **10**.

The model compounds were designed based on a common modular concept for rigid molecular scaffold syntheses [50,51]. Each compound is composed of two building blocks, the paramagnetic trityl radicals and a diamagnetic bridge connecting them via ester groups. The bridging molecule **8** is a commercially available biphenyl while the bridging structures **9** and **10** are built of *para*-phenylene ethynylene and *meta*-phenylene ethynylene groups (Scheme 1).

Compounds **9** and **10** were synthesized by Sonogashira cross coupling of the commercially available 1,3-diethynyl benzene **11** with 4-bromo-4′-hydroxy biphenyl **12** and 1,3,5-tritethynyl benzene **13** with 4-hydroxy-4′-iodo biphenyl **14**, respectively. Compared to the literature [50], which employs the iodo derivative **14** for both the syntheses of **9** and **10**, the coupling of bromo derivative **12** to 1,3-diethynyl benzene **11** gave a significantly lower yield of compound **9** (570 mg, 1.23 mmol, 34% vs. lit.: 69%) under otherwise identical reaction conditions. This is due to the lower reactivity of the bromo derivative **12** in the oxidative addition step with the palladium catalyst [58]. Spacer molecule **10** was isolated in 55% yield (602 mg, 920 µmol; lit.: 60%).

**Scheme 2:**
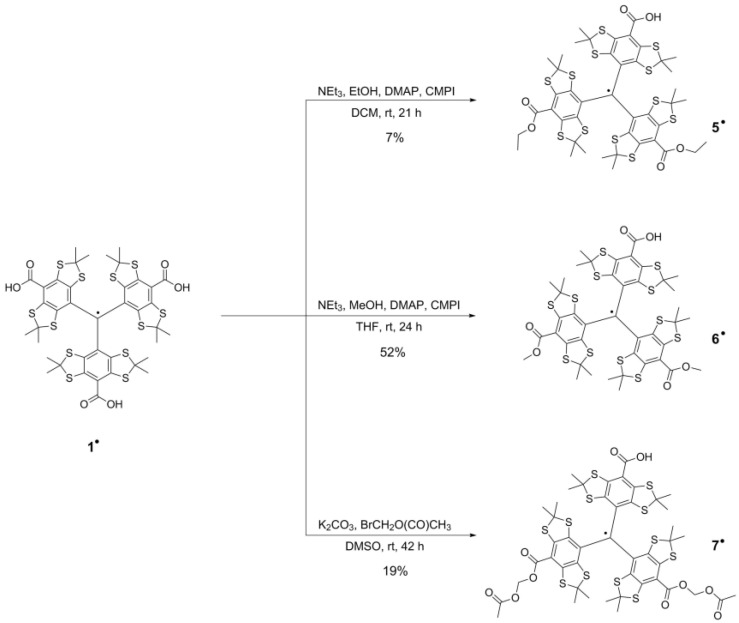
Synthesis scheme for the monofunctionalized trityl compounds **5^•^**, **6^•^** and **7^•^**.

As mentioned above, the trityl groups were then attached to the diamagnetic bridges using esterification reactions. This means that Finland Trityl **1^•^** cannot be used in this final step, as it contains three carboxylic acid groups that are susceptible to esterification and would therefore lead to oligomerization products. To avoid such side reactions, two of the three carboxylic acid groups of Finland Trityl **1^•^** are first esterified in a statistical fashion (Scheme 2).

Diethyl ester trityl radical **5^•^** was prepared in a Mukaiyama esterification reaction with ethanol using dichloromethane (DCM) as the solvent. MALDI-MS (see Figure S4 in the Supplementary Materials) showed that the product had partially undergone trans-esterification reactions with the methanol component of the eluent employed during the column chromatography purification step on silica gel. The resulting mixture of ester derivatives was separated via reversed phase high pressure liquid chromatography (HPLC) using a water gradient in acetonitrile (see Figure S5 in the Supplementary Materials) and gave 84.9 mg (80.5 µmol, 7%) of isolated compound **5^•^** as well as 158 mg (154 µmol, 25%) of compound **6^•^**. In a second approach, the solvent and reactant alcohol were changed to tetrahydrofuran (THF) and methanol, respectively, which gave 300 mg (292 µmol, 52%) of compound **6^•^**. The third monofunctionalization of **1^•^** towards compound **7^•^** (170 mg, 149 µmol, 19%) is achieved with a 19% yield via a S_N_2 reaction of the trityl acid groups with bromo-methyl acetate ester and was performed following a literature procedure [59].

**Scheme 3:**
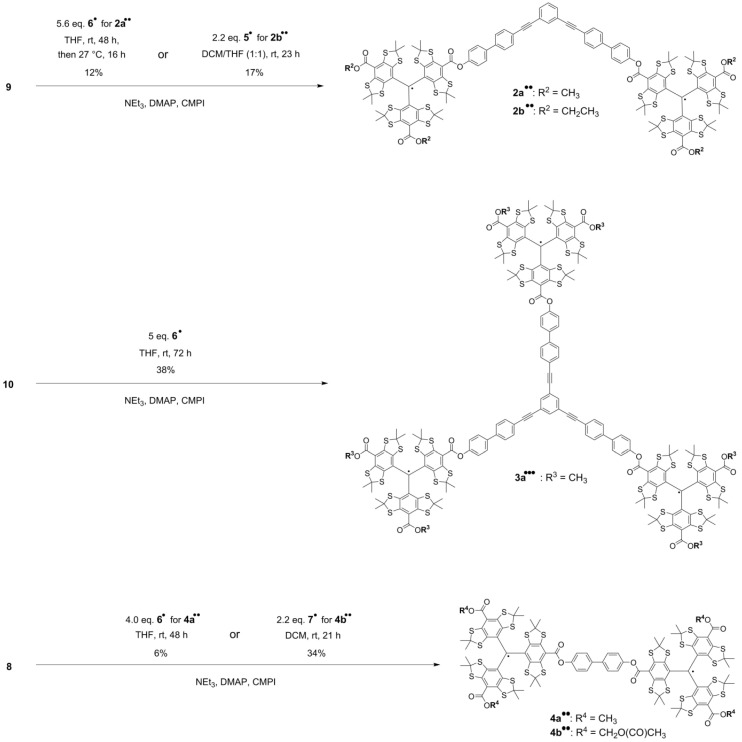
Synthesis scheme for model compounds **2a^••^**, **2b^••^**, **3a^•••^**, **4a^••^** and **4b^••^**.

The final esterification reactions towards model compounds **2a^••^**, **2b^••^**, **3a^•••^**, **4a^••^** and **4b^••^** are summarized in Scheme 3.

The purification of the model compounds proved to be challenging, since all compounds were obtained in a mixture with only partially esterified spacer molecules as well as unreacted monofunctionalized trityl radicals **5^•^**, **6^•^** or **7^•^**. The product compounds were isolated via reversed phase middle pressure liquid chromatography (MPLC) using first a water gradient (30–0% *v*/*v*) in acetonitrile to elute by-products and then THF to elute the product (Figures S15, S23 and S28). If necessary, a subsequent liquid chromatography step on silica using 1% *v*/*v* acetonitrile in chloroform was performed for separating the remaining by-products. In all cases, product amounts were lost due to mixed fractions obtained by the chromatography procedures, which contributed to the rather low reaction yields ranging from 6% to 38%. Especially low yields were obtained for compounds **2a^••^** (12%) and **4a^••^** (6%). For compound **4a^••^**, one-, two- and threefold oxidation by-products were observed after the reversed phase MPLC purification step at ~5 bar (Figure S28), resulting in an inseparable mixture which contained another ~60% worth of yield. According to the mass spectrometry analysis (Figure S29), several signals showing cumulative mass differences of Δ*m*/*z* = 16 next to the product compound are obtained. As this mass distribution pattern is not observed for the crude product (Figure S27), these oxidations are probably due to a reaction of the trityl’s sulfur atoms to form sulfoxides during the purification procedure. This kind of oxidation was also observed for compound **4b^••^** during size exclusion chromatography via gel permeation (GPC) at ~80 bar (Figures S35 and S36). Some trityl compounds seem thus to be sensitive to the combination of elevated pressure and oxygenated solvent. The phenomenon was not observed in any MPLC purification step of the other reported trityl compounds. Finally, all five model systems were characterized by cw EPR (see Section 2.2) and MALDI-MS, the latter of which yielded experimental masses that fitted the calculated ones (see Supplementary Materials).

### 2.2. EPR Spectroscopy

**Figure 3:**
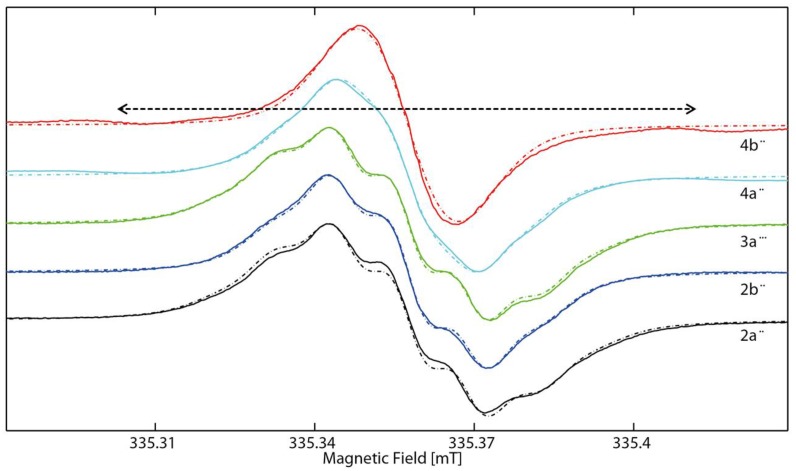
CW EPR spectra of **2a^••^**, **2b^••^**, **3a^•••^**, **4a^••^** and **4b^••^** (full lines) in degassed CD_2_Cl_2_/MeOD (2:1) at 293 K and simulations thereof (dashed lines) obtained using EasySpin [60]. The dashed arrow indicates the splitting of the *meta*-^13^C satellites in **4a^••^** and **4b^••^**, amounting to ~0.1 mT (for details, see text below).

Figure 3 shows the main absorption line of the cw X-band EPR spectra for compounds **2a^••^**, **2b^••^**, **3a^•••^**, **4a^••^** and **4b^••^**. This line is caused by molecules, which do not contain any ^13^C nuclei, while those molecules that contain one ^13^C nucleus give rise to satellite lines, which will be discussed below.

For all molecules, narrow main absorption lines with peak to peak line widths of ≤0.03 mT were found at isotropic *g*-values of 2.00344–2.00351. Furthermore, in all cases except for **4b^••^**, it was possible to resolve ^1^H hyperfine coupling to the *α*-protons of the carboxylate substituents. For molecules **2a^••^**, **2b^••^** and **3a^•••^**, these were taken into account in the simulations by introducing isotropic hyperfine coupling constants of *a*(*α*-*H*) = 0.3 MHz and the appropriate number of equivalent hydrogen nuclei, i.e., six *α*-protons for the methyl carboxylate compounds **2a^••^** and **3a^•••^** and four *α*-protons for the ethyl substituted compound **2b^••^**. In contrast, the main lines of **4a^••^** and **4b^••^** are found to be narrower than for the other compounds. In addition, the satellite lines stemming from hyperfine coupling to ^13^C nuclei are already visible at both sides of the central lines for **4a^••^** and **4b^••^**. The reason for that finding is that biradicals **4a^••^** and **4b^••^** exhibit isotropic exchange coupling. The exchange coupling constant *J* amounts to 75 MHz (details see below) and clearly exceeds the isotropic hyperfine coupling constants *a*(*α*-*H*) of the *α*-protons of the carboxylate substituents as well as the coupling constants of the satellite lines (details see below). This means that the condition for strong coupling (*|J|* >> |Δ*ω*|) is fulfilled and the two spin centers have to be treated as a spin pair, which is delocalized over both trityl rings. Consequently, the observed coupling constant is reduced by 50% and the number of equivalent nuclei is twice as high as in the localized case. Indeed, it was possible to simulate the spectrum of **4a^••^** by introducing 12 equivalent *α*-protons with an apparent isotropic coupling constant of *a*(*α*-*H*) = 0.15 MHz, i.e., half the value observed before for **2a^••^**, **2b^••^** and **3a^•••^**. Similarly, the spectrum of **4b^••^** could be simulated using the same line widths that have also been employed for the other radicals, eight equivalent nuclei, and a coupling constant of 0.13 MHz. Table 1 summarizes the essential simulation parameters of all compounds.

**Figure 4:**
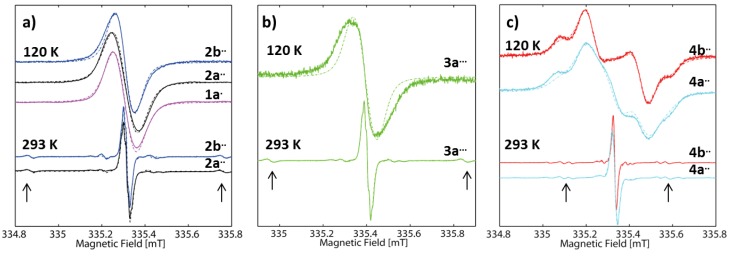
CW EPR spectra of: (**a**) **2a^••^** and **2b^••^**; (**b**) **3a^•••^**; and (**c**) **4a^••^** and **4b^••^** (full lines) in degassed CD_2_Cl_2_/MeOD (2:1) at 293 K and 120 K using an extended magnetic field axis. The color code is the same as in Figure 3. The dashed lines are simulations of the spectra obtained using EasySpin [60]. The arrows indicate the position of the satellite caused by the *o*-^13^C nuclei. In (**a**), the frozen solution spectrum of monotrityl **1a^•^** is given as reference spectrum.

Aside from the main absorption lines, a series of satellite lines stemming from ^13^C nuclei have been observed for each molecule. This is shown in Figure 4, where the spectra in Figure 3 are shown using an extended magnetic field axis. Additionally, the spectra of compounds **2a^••^**, **2b^••^**, **3a^•••^**, **4a^••^** and **4b^••^** recorded at 120 K in frozen solution are also shown in Figure 4 along with the frozen solution spectrum of **1a^•^**, which is used as reference.

**Figure 5:**
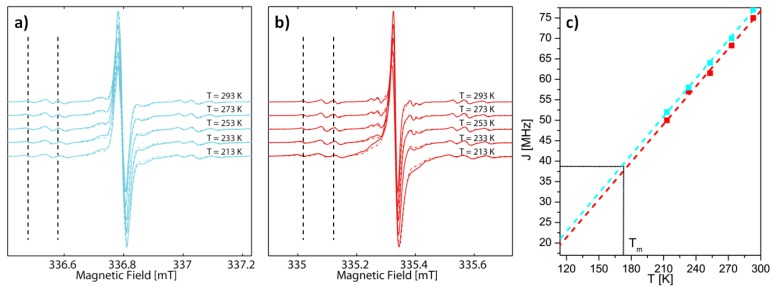
CW X-band EPR spectra of: (**a**) **4a^••^;** and (**b**) **4b^••^** in liquid solution at different temperatures. The vertical dashed lines are meant to visualize the temperature-dependent shift of the satellite lines. The full lines are the experimental and the underlying dotted ones the simulated spectra. The dependence of the magnitude of *J* on the temperature is shown for **4a^••^** (cyan) and **4b^••^** (red) (**c**). The lower bound of the *T* axis is equal to *T_g_* of the solvent system, *T_m_* is indicated in the graph.

It is worth noting that the observed ^13^C hyperfine coupling constants in the spectra obtained at 293 K are equal in all molecules investigated herein and amount to 31.25, 25.00, 6.20 and 8.40 MHz for the *ipso*, *ortho*, *meta* and *para* ^13^C nuclei, respectively. The assignment of the hyperfine coupling constants agrees with the assignment made by Kuzhelev et al. [61] and is also supported by DFT calculations (see Section 2.3). Despite having equal HFCCs, the satellite lines appear at half (*meta* and *para* satellites) or approximately half (*ipso* and *ortho* satellites) the distance from the main absorption lines in molecules **4a^••^** and **4b^••^** as compared to the other compounds. The reason for this is the occurrence of an isotropic exchange coupling, which was already mentioned before for the ^1^H hyperfine coupling. Since the *meta* and *para* satellites appear at half the distance observed in the compounds where no exchange coupling occurs, this indicates strong coupling. On the contrary, the position of the other satellite lines is not at exactly half the distance and furthermore, the number of satellite lines is increased as compared to the spectra of the compounds without exchange coupling. This is indicative of intermediate coupling regime. Indeed, it was possible to reproduce the position and the number of all appearing satellite lines by using an isotropic exchange coupling constant of *J*(**4a^••^**) = 77 MHz and *J*(**4b^••^**) = 75 MHz (a detailed theoretical treatment of the isotropic exchange interaction is given in the Supplementary Materials). Exchange coupling constants are often affected by conformational equilibria and therefore might change when the temperature is changed [62]. Since EPR based distance measurements are usually conducted in frozen solution, the values of *J* obtained at room temperature might be misleading when interpreting the data obtained in frozen solution. Therefore, *J* was recorded at various temperatures as shown in Figure 5.

As can be seen in Figure 5, the position of the satellites shifts more towards the central line by decreasing the temperature. This indicates that the exchange coupling constants decrease with temperature. Accessing values at temperatures below 213 K was not possible, as the EPR lines became considerably broader due to an increased viscosity of the solutions at such low temperatures. Therefore, it was no longer possible to identify the position of the satellite lines under these conditions. Importantly, the magnitude of the decrease as well as the absolute values of *J*, are similar for both compounds, although the values for **4a^••^** are slightly higher. Temperature dependences of similar magnitude have been reported before for exchange-coupled systems [54]. Furthermore, a recent study on mixed trityl-nitroxide biradical [63] and bis-(copper-porphyrine) [64] compounds also showed an approximately linear dependence of the exchange coupling constant *J* on the temperature *T* over a wide range of temperatures. Figure 5c allows estimating *J* in frozen solution by extrapolating the experimental data for *J*(*T*) to temperatures where conformational changes of the molecules are expected to be frozen out [54]. The solvent system itself is behaving like a solid below the glass transition temperatures *T_g_*, which can be estimated to amount to 2/3 of the melting point *T_m_* of the solvent system (MeOD/d_2_-DCM, 1:2 *v*/*v*) used for the EPR samples [65]. In between *T_g_* and *T_m_*, the solvent is expected to behave as a viscous, super cooled liquid. Here, the melting point is estimated to be similar to the melting points of the individual components of the solvent, amounting to 176 and 175 K, respectively. This assumption appears to be reasonable as the solvent mixture forms a transparent glass, indicating that the components remain completely mixed in the frozen state, which implies that there is no melting point depression due to removal of mixing entropy upon freezing. Thus, with an approximate melting point *T_m_* of ~175 K the glass transition temperature *T_g_* can be estimated to amount to ~115 K. The extrapolation to the estimated *T_g_* as shown in Figure 5c) yields values of *J*(**4a^••^**) = 22 MHz and *J*(**4b^••^**) = 20 MHz in frozen solution. Given the bulkiness of the trityl groups it might be possible that the conformational changes causing the decrease of *J* are already strongly hindered at higher temperatures and are thus preserved under rapid cooling conditions. Therefore, Figure 5c also indicates *T_m_* of the solvent system and gives an estimated upper bound of ~40 MHz for *J* in **4a^••^** and **4b^••^**.

**Figure 6:**
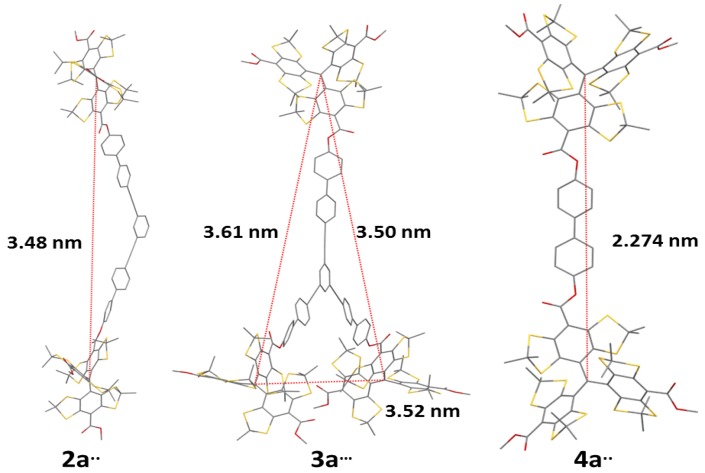
DFT optimized structures and inter-spin distances for compounds **2a^••^**, **3a^•••^** and **4a^••^**.

With the estimated values of *J*, it is possible to interpret the data obtained at 120 K shown in Figure 4, starting with the frozen solution spectra of **1a^•^**, **2a^••^** and **2b^••^**. A frozen solution of **1a^•^** gives rise to an almost isotropic line without any discernible hyperfine splitting at X-band MW frequency and a peak-to-peak line width of 2.36 MHz (Gaussian contribution) and 1.00 MHz (Lorentzian contribution, the simulation parameters for the frozen solution spectra are listed in Table 2). To account for the slight anisotropy, a small *g* anisotropy was introduced, in agreement with observations made previously on similar systems [66]. Compound **2a^••^** in comparison to **1a^•^** has a slightly broader spectrum, despite the similar methyl substitution pattern. It is possible to obtain a good simulation of this spectrum by using line width parameters identical to those of **1a^•^** but adding a dipolar electron–electron interaction with a coupling constant of *D* = 1.2 MHz, corresponding to a spin–spin distance of 3.51 nm (Equation (5)), in excellent agreement with the values obtained by DFT calculations (see Section 2.3) given in Figure 6. However, the spectrum of compound **2b^••^** is narrower than the spectrum of compounds **2a^••^** and even **1a^•^**, despite the presence of similar dipolar electron–electron interaction in **2b^••^** as in **2a^••^**. This indicates that the hyperfine coupling appears to have a larger effect on the line width than the dipolar coupling in compounds with inter-spin distances of about 3.5 nm.

In Figure 4b, the spectrum of **3a^•••^** is considered. Again, an almost isotropic line is observed, which is the broadest of all spectra discussed so far. The presence of three spins in **3a^•••^** was accounted for in EasySpin by including three dipolar electron–electron interaction tensors, which are tilted by 60° with respect to each other, as the geometry of the molecule implies. This led to a notable increase in the width of the simulated spectrum as compared to the simulation performed for **2a^••^**, in agreement with experimental observations. This is reasonable, as the situation of one electron coupling to two others is comparable to situations where hyperfine coupling to several nuclei occurs: the more coupling nuclei are present, the broader the EPR spectrum. However, the broadening in the simulation is much less pronounced than the experimentally observed broadening. Thus, the increased dipolar interaction in **3a^•••^** as compared to **2a^••^** appears not to be the primary cause of line broadening in the case at hand, and other terms in the spin Hamiltonian like *g*- or hyperfine-strain appear to be dominant. 

Therefore, the data on **1a^•^**, **2a^••^**, **2b^••^** and **3a^•••^** indicate that it is in principle possible to obtain distance information by cw EPR spectroscopy even for distances exceeding 3 nm. However, line width alterations due to hyperfine coupling appear to be more important than broadening due to dipolar coupling. Therefore, trustworthy information is only obtainable in model cases like the one discussed herein for such large distances.

The corresponding spectra for biradicals **4a^••^** and **4b^••^** are shown in Figure 4c. These are clearly different from the spectra discussed so far and resemble the shape of a Pake pattern. To obtain a good simulation of the spectra, it was necessary to introduce a dipolar coupling constant of 7.0(1) MHz (all other parameters remain unchanged). Here, occurrence of strong coupling is expected, as *J* clearly exceeds the line width of ~3 MHz of the trityl spectrum in frozen solution. Therefore, Equation (8) is used to obtain the interspin distance instead of Equation (5), yielding a value of 2.23(1) nm. This value is in nice agreement with the value obtained by DFT calculations (see Figure 6). Finally, the spectra of both **4a^••^** and **4b^••^** show contributions stemming from mono-radical impurities. To obtain a good simulation for **4a^••^**, it was necessary to include a contribution of about 15% monoradical for **4a^••^** (which is also visible in the liquid solution spectra in the satellite regions). Similarly, the spectrum of **4b^••^** could be analyzed by taking into account a 5% contribution of monoradical. The low monoradical contributions are also seen in the mass spectra.

### 2.3. DFT Calculations

To obtain an estimate for the inter-spin distances, DFT structure optimizations have been performed for the methyl compounds **2a^••^**, **3a^•••^** and **4a^••^**. The obtained structures are shown in Figure 6. It is worth noting that slight differences in the interspin distances in **2a^••^** and **3a^•••^** occur despite having similar diamagnetic bridges. This reflects the many degrees of freedom of the diamagnetic backbone, which presumably leads to a flat potential energy surface with the occurrence of many local minima, each having slightly different interspin separations. The occurrence of such conformers with slightly different end-to-end distances in a molecule has been described for such phenylene ethynylene systems [51,67,68]. However, the limited resolution of the cw EPR spectra discussed herein does not allow commenting on the width of the interspin distance distribution and requires the use of pulsed EPR methods.

Furthermore, the hyperfine coupling constants of the ^13^C nuclei have been calculated to allow assignment of the ^13^C satellite lines to the atoms. The obtained hyperfine couplings are listed in Table 3 and are in qualitative agreement with the values given by Kuzhelev et al. [61] and with the values used for the simulations.

## 3. Materials and Methods 

### 3.1. General Procedures

Commercially available chemicals and solvents were used without further purification. All air-sensitive reactions were performed using standard Schlenk techniques under an argon atmosphere. Work-up and chromatography solvents were either used in “p.a.” and “hplc grade” quality or purified by distillation (DCM, cyclohexane, ethyl acetate ester). Thin layer chromatography (TLC) was conducted on 250 µm Merck silica plates (60 F254) containing a fluorescent indicator with a wavelength of 254 nm, which was visualized by a UV lamp (UVP, Cambridge, UK). Silica column chromatography was performed manually using E. Merck silica gel (60 Å pore size, 40–63 µm particle size, 230–400 mesh). Reversed phase middle pressure liquid chromatography (MPLC) was carried out using a Sepacore^®^ X10 system by Büchi (Essen, Germany) in combination with Büchi FlashPure EcoFlex C18 20 g cartridges. Reversed phase high pressure liquid chromatography (HPLC) was performed using a PLATINblue^®^ system by KNAUER (Berlin, Germany) in combination with a KNAUER Eurospher II column (100-5 C18P; 5 µm; 4,0 × 250 mm with integrated pre-column—485 nm). Gel permeation chromatography (GPC) was carried out in THF at room temperature using a Shimadzu recycling GPC system in combination with a four-column set from PSS Polymer Standard Service GmbH (polysterol, 8 mm × 300 mm, porosity 102 Å, 103 Å, 105 Å, with pre-column). All solvents were removed under reduced pressure by a rotary evaporator. Products were dried under reduced pressure using an oil-sealed two-stage mechanical pump.

### 3.2. Nuclear Magnetic Resonance Spectroscopy (NMR)

^1^H-NMR and ^13^C-NMR spectra were recorded on Bruker DPX 300, DPX 400 and DMX 500 instruments (Bruker BioSpin, Rheinstetten, Germany) with 300.1, 400.1, and 500.1 MHz for ^1^H, and 75.5, 100.6, and 125.8 MHz for ^13^C. Chemical shifts are given in parts per million (ppm) referenced to residual ^1^H or ^13^C signals of the employed deuterated solvents (chloroform-*d*: 7.26 ppm, dimethyl sulfoxide-*d*6: 2.50 ppm). Spectra were recorded at room temperature.

### 3.3. Mass Spectometry (MS)

Electrospray Ionization Mass Spectrometry (ESI-MS) spectra were recorded on a Bruker Daltonics micrOTOF-Q time of flight spectrometer (BrukerBioSpin, Rheinstetten, Germany). Matrix Assisted Laser Desorption Ionization (MALDI-MS) spectra were recorded on a Bruker Daltonics autoflex TOF/TOF time of flight spectrometer (BrukerBioSpin, Rheinstetten, Germany), using DCTB as matrix material.

### 3.4. EPR Sample Preparation

All EPR samples have been prepared in an argon atmosphere at a concentration of 50 µM using a 1:2 mixture of deuterated methanol and deuterated dichloromethane. The solvents were degassed using repeated freeze–pump–thaw cycles prior to use. The sample solutions were filled into 1-mm-diameter X-band EPR quartz tubes, which were slid into gas-tight Young tubes.

### 3.5. EPR Measurements

All cw EPR experiments were conducted at X-band frequency on a Bruker EMXmicro EPR spectrometer (Bruker BioSoin, Rheinstetten, Germany) with the EMX standard resonator (4119HS). Temperature adjustment was achieved using the ER 4131VT temperature control system and a flow of cold nitrogen gas.

### 3.6. DFT Calculations

DFT calculations have been performed using the unrestricted Kohn–Sham formalism with the BP86 functional, an SVP basis set, and D3 dispersion correction as implemented in ORCA [69,70,71,72,73].

## 4. Conclusions

Several trityl-based model systems have been prepared and characterized by MALDI-MS and cw EPR. It was observed that ethyl ester derivatives of Finland Trityl readily undergo trans-esterification reactions when exposed to methanol. In addition, trityl radical compounds **4a^••^** and **4b^••^** demonstrated to be sensitive towards the formation of sulfoxide derivatives in oxygenated solutions under elevated pressure (>5 bar), which imposes restrictions to the available purification methods. High-resolution cw EPR spectra at room temperature were obtained for all five trityl compounds, and it was possible to explain the obtained line shapes of the main absorption line by accounting for hyperfine coupling to the protons on the carboxylate substituents. Furthermore, it was possible to observe and analyze the satellite lines stemming from the ^13^C atoms in the phenyl rings of the trityl moieties. The most interesting case was encountered for compounds **4a^••^** and **4b^••^**, where temperature dependent exchange interaction between the two electrons occurred. It was possible to extract the exchange coupling constants *J* as a function of temperature, which allowed extrapolating the data to the melting point of the solvent and thus yielded an estimate of *J* in frozen solution. Additionally, the spectra in frozen solution were investigated. While the data obtained for **1a^•^**, **2a^••^**, **2b^••^** and **3a^•••^** suggests that it might be possible to obtain distance information using cw EPR spectroscopy for inter-spin distances of up to 3.5 nm, the results also indicated that other factors have larger effects on the width of the EPR spectrum. Thus, a reliable extraction of distance information is possible only in the most favorable cases. Compounds **4a^••^** and **4b^••^** on the other hand gave rise to EPR spectra, which resemble Pake patterns. Taking into account the occurrence of strong coupling, it was possible to obtain the inter-spin distance in very good agreement with DFT predictions for both model systems.

## Figures and Tables

**Table 1:** Simulation Parameters for the room temperature EPR spectra of **2a^••^**, **2b^••^**, **3a^•••^**, **4a^••^**, and **4b^••^**. The coupling to the ^13^C nuclei and exchange coupling constants are discussed in the text.

Compound	2a^••^	2b^••^	3a^•••^	4a^••^	4b^••^
g_iso_	2.00348	2.00344	2.00344	2.00351	2.00346
*a_α_*_-*H*_^a^ [MHz]	0.3	0.3	0.3	0.15	0.13
*n_α_*_-*H*_^b^	6	4	6	12	8
Lwpp ^c^ [MHz]	0.22/0.15	0.22/0.17	0.22/0.17	0.17/0.15	0.22/0.15

**^a^** The apparent hyperfine coupling constants of **4a^••^** and **4b^•••^** are reduced by a factor of 50% by the occurrence of strong exchange coupling. **^b^** Number of equivalent *α*-protons. **^c^** Peak-to-peak line width. The first number refers to the Gaussian contribution, the second one to the Lorentzian contribution.

**Table 2:** Simulation parameters for the frozen solution EPR spectra (T = 120 K) of **1a^•^**–**4b^••^**.

Compound	1a^•^	2a^••^	2b^••^	3a^•••^	4a^••^	4b^••^
*g* ^a^	2.00355 2.00355 2.00339	2.00354 2.00354 2.00338	2.00353 2.00353 2.00337	2.00351 2.00351 2.00335	2.00355 2.00355 2.00339	2.00351 2.00351 2.00335
*D* ^b^ [MHz]	-	1.2	1.2	1.2 (×2)	7.0	7.0
Lwpp ^c^ [MHz]	2.36/1.00	2.36/1.00	1.57/1.00	2.36/1.00	2.36/1.00	1.26/1.26

^a^ Values correspond to *g_x_*, *g_y_*, and *g_z_*. ^b^ The parallel component of the *D* tensor was assumed to lie in the xy plane. In compound **3a^•••^**, each electron couples to two other electrons instead of one, which is indicated in the entry for *D*. For compounds **2a^••^**, **2b^••^** and **3a^•••^**, Equation (5) holds, whereas Equation (8) has to be used for compounds **4a^••^** and **4b^••^**. ^c^ Peak-to-peak line width. The first number refers to the Gaussian contribution, the second one to the Lorentzian contribution.

**Table 3:** Hyperfine coupling constants (in MHz) of the carbon atoms on the phenyl rings of trityl compounds.

Source	*i*-^13^C	*o*-^13^C	*m*-^13^C	*p*-^13^C
Kuzhelev ^a^	31.1	25.2	6.7	9.2
This work, exp	31.3	25.0	6.2	8.4
This work, DFT ^b^	−37.3	34.6	−10.6	15.8

^a^ Kuzhelev’s publication [61] indicates a slight dependence of the hyperfine coupling on the substitution pattern, the values cited here correspond to the compound labeled “FD” by Kuzhelev. ^b^ DFT provides the sign of the hyperfine coupling constants, which cannot be obtained from the cw EPR spectrum.

## Supplementary Materials

The following are available online, detailed synthesis procedure descriptions, analytical data, xyz coordinates obtained by DFT calculations.
