# Supplementary material for: Synthesis of Nanometer Sized Bis- and Tris-trityl Model Compounds with Different Extent of Spin–Spin Coupling
Source: Molecules. 2018 Mar 17;23(3):682. doi: 10.3390/molecules23030682 (PMC6017437; doi:10.3390/molecules23030682)
Supplement: Supplementary file 1 [file molecules-23-00682-s001.pdf]

Synthesis of Nanometer Sized Bis- and Tris-trityl Model Compounds with Different Extent of Spin-Spin Coupling

*J. J. Jassoy[‡], A. Meyer[‡], S. Spicher, C. Wuebben, O. Schiemann**

Supporting Information

Content

1. Syntheses 2

2. NMR, Mass Spectrometry and Chromatography Data 16

3. Wavefunctions and energies in the different coupling regimes 35

4. XYZ Coordinates of **2a**$^{\bullet\bullet}$, **3a**$^{\bullet\bullet\bullet}$ and **4a**$^{\bullet\bullet}$ obtained by DFT 45

5. References 74

1. Syntheses

1.1 Synthesis of 4-{4-[2-(3-{2-[4-(4-Hydroxyphenyl)phenyl]ethynyl} phenyl)ethynyl]phenyl}phenol **9***.*

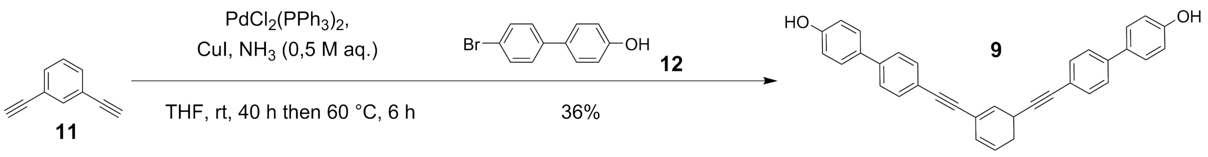

The synthesis of **9** was carried out following a modified procedure by Valera *et al.*[1]

Under an argon atmosphere, 4-Hydroxy-4'-iodobiphenyl (2.00 g, 6.78 mmol) was dissolved in dry tetrahydrofuran (THF, 20 ml) before $PdCl_2(PPh_3)_2$ (79.1 mg, 110 µmol) and CuI (21.5 mg, 110 µmol) were added. The resulting yellow solution was degassed via three freeze-pump-thaw cycles. 1,3-Diethynylbenzene (450 µl, 3.39 mmol) was added dropwise as a degassed solution in THF (5 ml). The reaction mixture was degassed once more before dropwise addition of an aqueous ammonia solution (0.5 M, 25 ml) which had been saturated with argon for 10 minutes. The mixture was stirred at room temperature for 40 hours and afterwards heated for 6 hours at 60 °C. The two phases were separated and the aqueous layer was extracted with ethyl acetate ester (EE) (2×20 ml). The combined organic layers were washed with 10% HCl (40 ml), water (40 ml) and brine (20 ml) before being dried over $MgSO_4$. After filtration, all solvents were removed under reduced pressure. The crude product was purified via silica column chromatography using chloroform followed by 1-100% EE/chloroform as eluents. The product compound **9** was obtained as a pale brown solid (570 mg, 1.23 mmol, 36%).

^{1}H-NMR (400 MHz, DMSO-d_6, rt) δ [ppm] = 9.66 (s, 2H), 7.81-7.47 (m, 16H), 6.87 (d, J = 8.6 Hz, 4H).

1.2 Synthesis of 4-(4-{2-[3,5-Bis({2-[4-(4-hydroxyphenyl)phenyl] ethynyl}) phenyl]ethynyl}phenyl)phenol **10**.

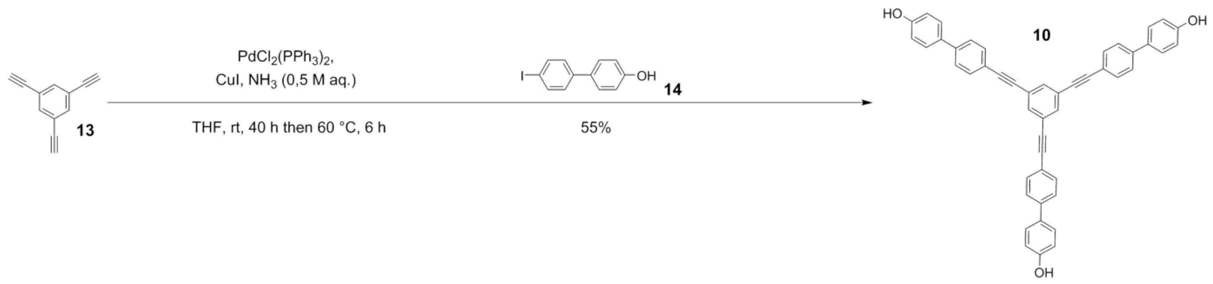

The synthesis of **10** was carried out following a modified procedure by Valera *et al*.[1]

Under an argon atmosphere, 4-Hydroxy-4'-iodobiphenyl (1.48 g, 4.99 mmol) was dissolved in dry tetrahydrofuran (THF, 75 ml) before $PdCl_2(PPh_3)_2$ (117 mg, 170 µmol) and CuI (21.3 mg, 100 µmol) were added. The resulting yellow solution was degassed via three freeze-pump-thaw cycles. 1,3,5-Triethynylbenzene (250 mg, 1.67 mmol) was added portionwise as a solid. The reaction mixture was degassed once more before dropwise addition of an aqueous ammonia solution (0.5 M, 75 ml) which had been saturated with argon for 10 minutes. The mixture was stirred at room temperature for 40 hours and afterwards heated for 6 hours at 60 °C. The two phases were separated and the aqueous layer was extracted with EE (2×50 ml). The combined organic layers were washed with 10% HCl (80 ml), water (80 ml) and brine (40 ml) before being dried over $MgSO_4$. After filtration, all solvents were removed under reduced pressure. The crude product was purified via silica column chromatography using chloroform followed by 1-100% methanol/chloroform as eluents. All solvents were removed under reduced pressure and the remaining solid was washed in dichloromethane (DCM) for 15 hours and then filtered. The collected solid was dried under reduced pressure. Product compound **10** was obtained as a pale brown solid (602 mg, 920 µmol, 55%).

^{1}H-NMR (400 MHz, DMSO-d$_6$, rt) δ [ppm] = 9.66 (s, 3H), 7.77-7.56(m, 21H), 6.87 (d, J = 8.6 Hz, 6H).

MS (MALDI+) *m/z*: 654.2 (100; $[C_{48}H_{30}O_3]^{\bullet +}$; $[M]^{\bullet +}$), 904.4 (5; $[M+DCTB]^{\bullet +}$). DCTB = *trans*-2-[3-(4-*tert*-Butylphenyl)-2-methyl-2-propen ylidene] malononitrile, matrix substance with M = 250.34 g/mol.

1.2 Synthesis of Bis-(8-ethoxycarbonyl-2,2,6,6-tetramethylbenzo[1,2-d;4,5-d´]-bis[1,3]dithiol-4-yl)mono(8-carboxyl-2,2,6,6-tetramethylbenzo [1,2-d;4,5-d´]-bis[1,3]dithiol-4-yl)methyl radical $\mathbf{5^{\bullet}}$*.*

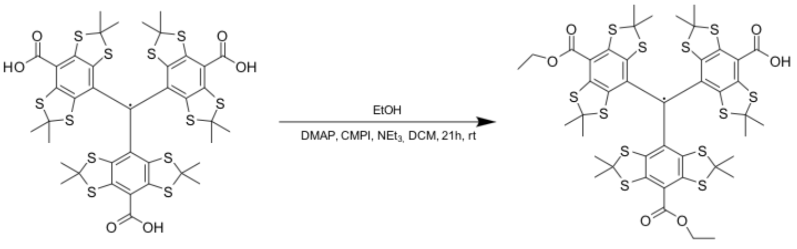

$\mathbf{1^{\bullet}}$ $\mathbf{5^{\bullet}}$

Compound $\mathbf{5^{\bullet}}$ was synthesized following a modified procedure by Saigo *et al*.[2]

Compound $\mathbf{1^{\bullet}}$ (1.16 g, 1.16 mmol) as well as the reactants 2-chloromethylpyridinium iodide (CMPI, 780 mg, 2.78 mmol) and 4-dimethyl- aminopyridine (DMAP, 113 mg, 930 µmol) were dried under reduced pressure for two hours before starting the reaction. Dry dichloromethane (15 ml) and triethylamine (Et_3N, 772 µL, 5.56 mmol) were degassed together via three freeze-pump-thaw cycles.

Under an argon atmosphere, compound $\mathbf{1^{\bullet}}$ was dissolved in the degassed mixture of DCM and Et_3N and stirred at room temperature for two hours to achieve complete deprotonation of the carboxylic acid groups. After addition of EtOH (135 µl, 2.32 mmol), the green reaction mixture was cooled down to 0 °C. CMPI and DMAP were added which resulted in a dark

red color. The mixture was stirred at room temperature for 21 hours and then quenched with aqueous HCl (30 ml, 0.3 M). The organic layer was separated and the aqueous layer was extracted with DCM (3×20 ml). The combined organic layers were washed with brine (30 ml) and all solvents were removed under reduced pressure at 30 °C to give the crude product. Purification was performed by silica column chromatography using chloroform followed by 1-100 % methanol/chloroform as eluents. MALDI-MS showed a mixture of methyl and ethyl ester substitution patterns for the trityl radical (see Figure S4) which are a result of trans-esterification reactions with the methanol containing eluent during the preceding silica column chromatography step. The mixture was separated by reversed phase high pressure liquid chromatography (HPLC) using a water gradient in acetonitrile (see Figure S5) and product compound **5**$^{\bullet}$ was obtained as a dark brownish red solid (84.9 mg, 80.5 µmol, 7%).

MS (MALDI+) *m/z*: 1055.1 (100; $[C_{44}H_{47}O_6S_{12}]^{\bullet+}$;$[M]^{\bullet+}$), 1041.1 (4; $[C_{43}H_{45}O_6S_{12}]^{\bullet+}$; (Mono-methyl-mono-ethyl ester trityl radical)), 983.1 (4; $[C_{41}H_{43}O_4S_{12}]^{\bullet+}$; (onefold decarboxylated dimethyl ester trityl radical)).

Additionally, the dimethyl ester derivative **6**$^{\bullet}$ was isolated as a greenish brown solid (158 mg, 154 µmol, 25%).

MS (MALDI+) *m/z*: 1027.1 (100; $[C_{42}H_{43}O_6S_{12}]^{\bullet+}$; $[M]^{\bullet+}$).

1.3 Synthesis of Bis-(8-methoxycarbonyl-2,2,6,6-tetramethylbenzo[1,2-d;4,5-d´]-bis[1,3]dithiol-4-yl)mono(8-carboxyl-2,2,6,6-tetramethylbenzo [1,2-d;4,5-d´]-bis[1,3]dithiol-4-yl)methyl radical **6•**.

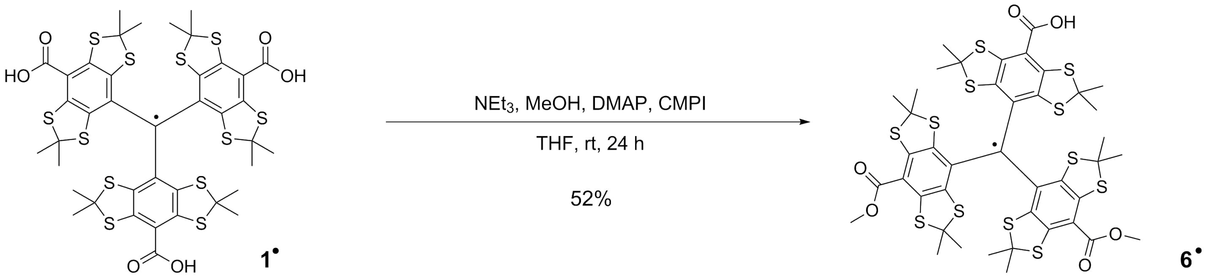

Compound **6•** was synthesized following a modified procedure by Saigo *et al*.[2]

Compound **1•** (562 mg, 562 µmol) as well as the reactants 2-chloromethylpyridinium iodide (CMPI, 351 mg, 1.38 mmol) and 4-dimethyl- aminopyridine (DMAP, 55.4 mg, 450 µmol) were dried under reduced pressure for two hours before starting the reaction.

Under an argon atmosphere, compound **1•** was dissolved in a mixture of dry tetrahydrofuran (THF, 7 ml) and triethylamine (Et_3N, 390 µL, 2.81 mmol) and stirred at room temperature for three hours to achieve complete deprotonation of the carboxylic acid groups. After addition of MeOH (45.6 µl, 1.12 mmol), the green reaction mixture was cooled down to 0 °C. CMPI and DMAP were added which resulted in a dark red color. The mixture was stirred at room temperature for 24 hours and then quenched with aqueous HCl (24 ml, 0.1 M). The organic layer was separated and the aqueous layer was extracted with DCM (3×10 ml). The combined organic layers were washed with brine (10 ml) and all solvents were removed under reduced pressure at 30 °C to give the crude product. Purification was performed by two subsequential silica column chromatography using 1) 50:50% v/v to 66:33% v/v DCM/cyclohexane (CH), then DCM, then 50:50% v/v DCM/EE, then EE, then 50:50% v/v EE/MeOH to 33:66% v/v EE/MeOH, then MeOH as eluents during the first

column and 2) EE as eluent for the second column. Product compound **6•** was obtained as a greenish brown solid (300 mg, 292 µmol, 52%). The compound´s purity was additionally assessed by HPLC (see Figure S9).

MS (MALDI+) *m/z*: 1027.0 (100; $[C_{42}H_{43}O_6S_{12}]^{\bullet +}$; $[M]^{\bullet +}$).

1.4 Synthesis of Bis-(8-acetoxymethoxycarbonyl-2,2,6,6-tetramethyl benzo[1,2-d;4,5-d´]-bis[1,3]dithiol-4-yl)mono(8-carboxyl-2,2,6,6-tetra methylbenzo[1,2-d;4,5-d´]-bis[1,3]dithiol-4-yl)methyl radical ***7•****.*

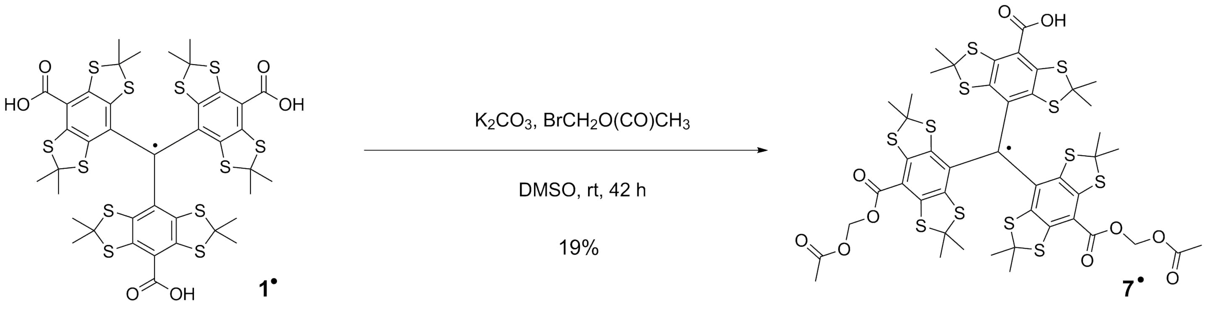

Compound **7•** was synthesized following a modified procedure by Liu *et al*.[3]

Under an atmosphere of argon, compound **1•** (779 mg, 779 µmol) was dissolved in dimethyl sulfoxide (30 ml) and potassium carbonate (926 mg, 5.45 mmol) was added. After stirring for 2 h at room temperature, bromomethyl acetate (200 µl, 1.95 mmol) was injected into the reaction mixture which then was stirred at room temperature for another 42 h. The reaction was diluted with water (60 ml) and DCM (60 ml), the phases were separated and the aqueous layer was extracted with DCM (5×20 mL). The combined organic layers were washed with brine (40 mL) and concentrated under reduced pressure. The resulting suspension was treated with aqueous HCl (30 ml, 0.3 M) and then extracted with DCM (3×40 ml) yet again. After concentration of the combined organic layers under reduced pressure, a brown solid was obtained. The crude product

was purified by column chromatography using chloroform followed by 5-100% v/v acetonitrile/chloroform and then 3-100% v/v methanol/acetonitrile as eluents. Product compound **7**$^{\bullet}$ was obtained as a dark brown solid (170 mg, 149 µmol, 19%).

MS (MALDI+) *m/z*: 1143.0 (100; $[C_{46}H_{47}O_{10}S_{12}]^{\bullet+}$; $[M]^{\bullet+}$).

1.5 Synthesis of bis-trityl compound **2a**$^{\bullet\bullet}$*.*

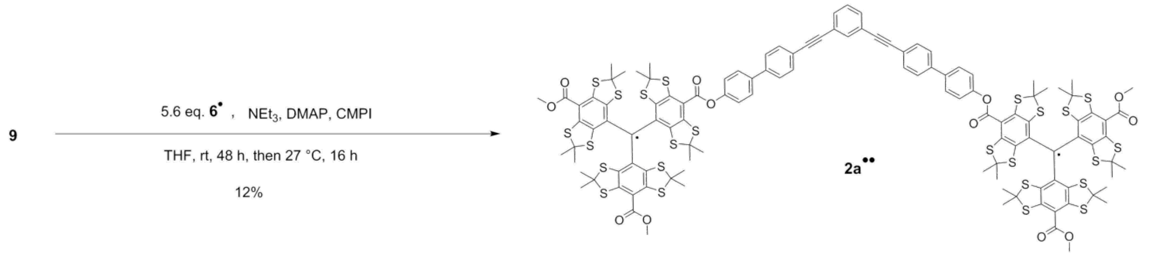

Compound **2a**$^{\bullet\bullet}$ was synthesized following a modified procedure by Saigo *et al*.[2]

Compound **6**$^{\bullet}$ (195 mg, 190 µmol) as well as the reactants 2-chloromethylpyridinium iodide (CMPI, 130 mg, 512 µmol) and 4-dimethyl-aminopyridine (DMAP, 20.4 mg, 167 µmol) were dried under reduced pressure for two hours before starting the reaction.

Under an argon atmosphere, compound **6**$^{\bullet}$ was dissolved in a mixture of dry THF (6 ml) with triethylamine (143 µL, 1.05 mmol), and stirred at room temperature for two hours to achieve complete deprotonation of the carboxylic acid groups. After addition of compound **9** (15.7 mg, 34.0 µmol), the reaction mixture was cooled down to 0 °C before CMPI and DMAP were added. The mixture was stirred at room temperature for 48 h and then warmed to 27 °C for another 16 h. The reaction was quenched with aqueous HCl (20 ml, 0.2 M) and diluted with 80 ml of DCM. The organic layer was separated and the aqueous layer was extracted with DCM (3×20 ml). The combined organic layers were washed with

brine (40 ml) and all solvents were removed under reduced pressure at 30 °C to give the crude product. MS-MALDI(+) analysis of the crude product indicated that the reaction had predominantly yielded a by-product (*m/z* = 2081, see Figure S11), while the expected target compound signal was not visible at all. The ^{1}H-NMR spectrum (Figure S12) of the isolated by-product showed strong paramagnetic contributions indicating that the by-product is a trityl radical containing compound. The by-product synthesis was presumably caused by an ethylene glycol contamination (from THF bottle packaging components or other), high resolution MS-APCI (atmospheric pressure chemical ionisation) gave a molecular formula which would be consistent with this hypothesis (see Figure S13 and Figure S14). A first purification step was performed by silica column chromatography using chloroform followed by 10% v/v acetonitrile in chloroform and finally 50% v/v methanol in chloroform as eluents. This step gave three fractions in the following order of mention: esterification reagents (fraction A), product and by-products (fraction B) and clean unreacted trityl **6$^{\bullet}$** (fraction C), the latter being thus easily regained for further usage. Fraction B was put to a second silica column chromatography procedure using a slowly increasing gradient of acetonitrile in chloroform. A third purification step consisted in a reversed phase middle pressure liquid chromatography (MPLC, see Figure S15) run using a water gradient in acetonitrile as eluent. After the collection of four impurity fractions, the column was rinsed with THF to collect the product containing fraction. The latter was purified by a final silica gel chromatography column using 1% v/v acetonitrile in chloroform as eluent. Product compound **2a$^{\bullet\bullet}$** was obtained as a brown solid (10.4 mg, 4.19 µmol, 12%). The compound´s purity was additionally assessed by MPLC (see Figure S17).

MS (MALDI+) *m/z*: 2482.8 (100; $[C_{118}H_{105}O_{12}S_{24}]^{\bullet\bullet+}$; $[M+H]^{\bullet\bullet+}$).

1.6 Synthesis of bis-trityl compound ***2b••**.*

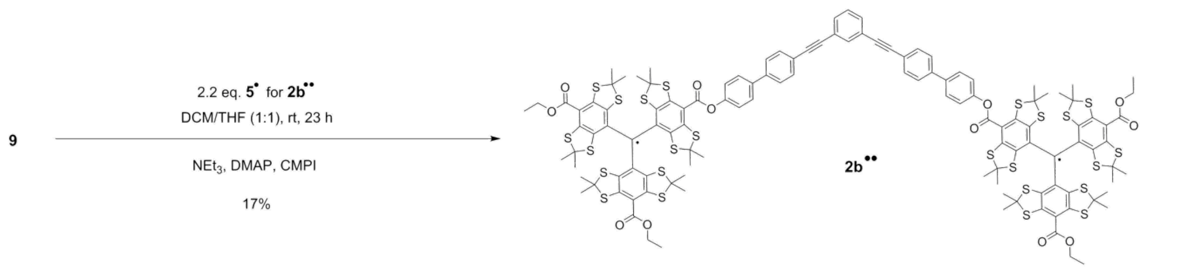

Compound **2b••** was synthesized following a modified procedure by Saigo *et al*.[2]

Compound **5•** (84.9 mg, 80.4 µmol) as well as the reactants 2-chloromethylpyridinium iodide (CMPI, 24.6 mg, 96.4 µmol) and 4-dimethyl- aminopyridine (DMAP, 3.93 mg, 32.1 µmol) were dried under reduced pressure for two hours before starting the reaction. Dry dichloromethane (DCM, 2 ml), dry tetrahydrofuran (THF, 2 ml) and triethylamine (26.7 µL, 192 µmol) were degassed together via three freeze-pump-thaw cycles.
Under an argon atmosphere, compound **5•** was dissolved in the degassed mixture of DCM, THF and triethylamine, and stirred at room temperature for two hours to achieve complete deprotonation of the carboxylic acid groups. After addition of compound **9** (16.7 mg, 36.2 µmol) the reaction mixture was cooled down to 0 °C before CMPI and DMAP were added. The mixture was stirred at room temperature for 23 hours and then quenched with aqueous HCl (15 ml, 0.3 M). The organic layer was separated and the aqueous layer was extracted with DCM (3×15 ml). The combined organic layers were washed with brine (20 ml) and all solvents were removed under reduced pressure at 30 °C to give the crude product. Purification was performed by silica column chromatography using chloroform followed by 1.5% v/v methanol in chloroform as eluents. A subsequent purification by size exclusion gel permeation chromatography (GPC, see Figure S20) was followed by a second silica gel chromatography column

using 1% v/v acetonitrile in chloroform to give product compound $\mathbf{2b}^{\bullet\bullet}$ as a brown solid (16.0 mg, 6.30 µmol, 17%). The compound´s purity was additionally assessed by MPLC (see Figure S22).

MS (MALDI+) *m/z*: 2536.1 (100; $[C_{122}H_{112}O_{12}S_{24}]^{\bullet\bullet+}$; $[M]^{\bullet\bullet+}$), 2522.1 (60; $[C_{121}H_{110}O_{12}S_{24}]^{\bullet\bullet+}$; $\mathbf{2b}^{\bullet\bullet}$ with 1 methyl ester and 3 ethyl esters), 1498.2 (33; $[C_{78}H_{67}O_{7}S_{12}]^{\bullet+}$; bridging molecule **9** with one trityl $\mathbf{5}^{\bullet}$), 2508.1 (12; $[C_{120}H_{108}O_{12}S_{24}]^{\bullet\bullet+}$; $\mathbf{2b}^{\bullet\bullet}$ with 2 methyl esters and 2 ethyl esters).

1.7 Synthesis of tris-trityl compound $\mathbf{3a}^{\bullet\bullet\bullet}$*.*

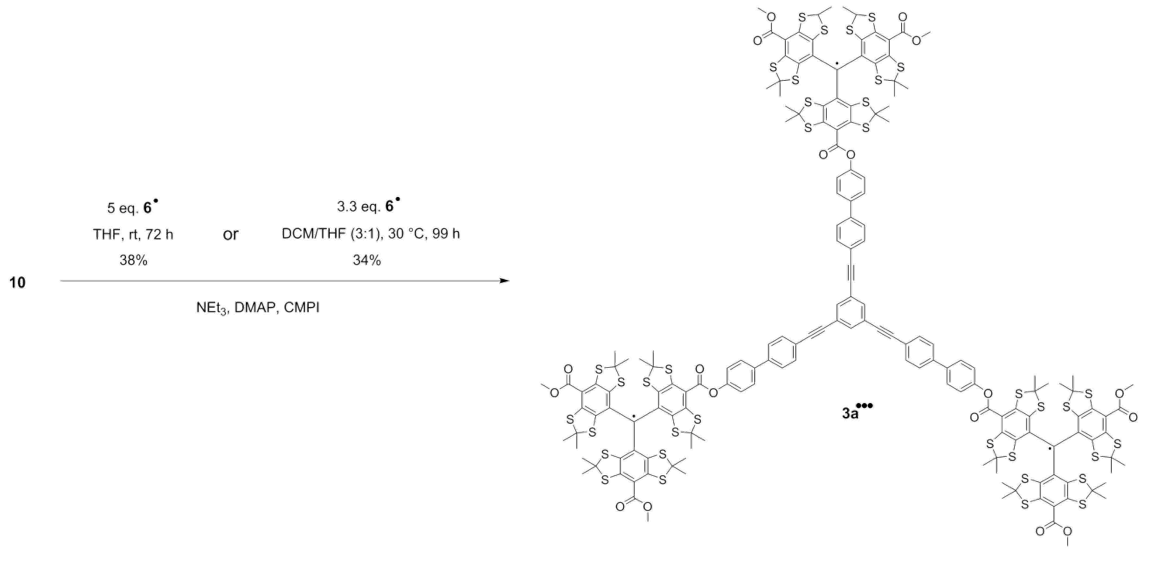

Compound $\mathbf{3a}^{\bullet\bullet\bullet}$ was synthesized following a modified procedure by Saigo *et al*.[2]

Method A: using THF as solvent.

Compound $\mathbf{6}^{\bullet}$ (104 mg, 101 µmol) as well as the reactants 2-chloromethylpyridinium iodide (CMPI, 63.2 mg, 248 µmol) and 4-dimethyl- aminopyridine (DMAP, 9.90 mg, 81.0 µmol) were dried under reduced pressure for two hours before starting the reaction.

Under an argon atmosphere, compound $\mathbf{6}^{\bullet}$ was dissolved in a mixture of dry THF (6 ml) with triethylamine (75.9 µL, 555 µmol), and stirred at

room temperature for two hours to achieve complete deprotonation of the carboxylic acid groups. After addition of compound **10** (13.2 mg, 20.0 µmol), the reaction mixture was cooled down to 0 °C before CMPI and DMAP were added. The mixture was stirred at room temperature for 72 h. The reaction was quenched with aqueous HCl (20 ml, 0.2 M) and diluted with 80 ml of DCM. The organic layer was separated and the aqueous layer was extracted with DCM (3×20 ml). The combined organic layers were washed with brine (40 ml) and all solvents were removed under reduced pressure at 30 °C to give the crude product. Purification was performed by reversed phase MPLC using a water gradient in acetonitrile as eluent (see Figure S23). After the collection of unreacted educt compound **6**$^{\bullet}$ and an impurity fraction, the column was rinsed with THF to collect the product compound in three fractions, one of which contained isolated target compound. Product compound **3a**$^{\bullet\bullet\bullet}$ was obtained as a brown solid (28.0 mg, 7.60 µmol, 38%). The compound´s purity was additionally assessed by MPLC (see Figure S25).

MS (MALDI+) *m/z*: 3684.8 (100; $[C_{174}H_{154}O_{18}S_{36}]^{\bullet\bullet\bullet+}$; $[M+H]^{\bullet\bullet\bullet+}$).

Method B: using DCM/THF (3/1) as solvent.

Compound **6**$^{\bullet}$ (47.2 mg, 45.9 µmol) as well as the reactants 2-chloromethylpyridinium iodide (CMPI, 14.1 mg, 55.1 µmol) and 4-dimethyl-aminopyridine (DMAP, 2.24 mg, 18.4 µmol) were dried under reduced pressure for two hours before starting the reaction. Dry dichloromethane (DCM, 6 ml), dry tetrahydrofuran (THF, 2 ml) and triethylamine (38.2 µL, 275 µmol) were degassed together via three freeze-pump-thaw cycles.

Under an argon atmosphere, compound **6**$^{\bullet}$ was dissolved in the degassed mixture of DCM, THF and triethylamine, and stirred at room temperature for two hours to achieve complete deprotonation of the carboxylic acid groups. After addition of compound **10** (9.01 mg, 13.8 µmol) the reaction

mixture was cooled down to 0 °C before CMPI and DMAP were added. The mixture was stirred for 99 hours at 30°C and then quenched with aqueous HCl (10 ml, 0.3 M). The organic layer was separated and the aqueous layer was extracted with DCM (3×10 ml). The combined organic layers were washed with brine (15 ml) and all solvents were removed under reduced pressure at 30 °C to give the crude product. Purification was performed by silica column chromatography using chloroform followed by 0.5% v/v methanol in chloroform as eluent. A second purification step using silica column chromatography with 1% v/v acetonitrile in chloroform gave compound **3a**$^{\bullet\bullet\bullet}$ as a brown solid (17.4 mg, 4.72 µmol, 34%).

MS (MALDI+) *m/z*: 3686.2 (100; $[C_{174}H_{154}O_{18}S_{36}]^{\bullet\bullet\bullet+}$; $[M+H]^{\bullet\bullet\bullet+}$), 2674.4 (100; $[C_{132}H_{112}O_{13}S_{24}]^{\bullet\bullet+}$; bridging molecule **10** with two trityls **6**$^{\bullet}$), 3936.5 and 2924.7 are the respective matrix (DCTB) aggregate signals of the formerly declared detections.

1.8 Synthesis of bis-trityl compound **4a**$^{\bullet\bullet}$*.*

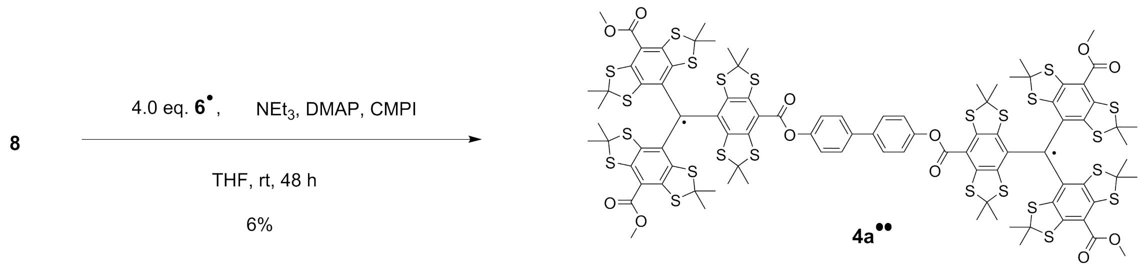

Compound **4a**$^{\bullet\bullet}$ was synthesized following a modified procedure by Saigo *et al.*[2]

Compound **6**$^{\bullet}$ (93.0 mg, 90.4 µmol) as well as the reactants 2-chloromethylpyridinium iodide (CMPI, 56.6 mg, 222 µmol) and 4-dimethyl- aminopyridine (DMAP, 8.84 mg, 72.3 µmol) were dried under reduced pressure for two hours before starting the reaction.

Under an argon atmosphere, compound **6**$^{\bullet}$ was dissolved in a mixture of

dry THF (6 ml) with triethylamine (67.8 µL, 497 µmol), and stirred at room temperature for two hours to achieve complete deprotonation of the carboxylic acid groups. After addition of compound **8** (4.20 mg, 22.6 µmol), the reaction mixture was cooled down to 0 °C before CMPI and DMAP were added. The mixture was stirred at room temperature for 48 h. The reaction was quenched with aqueous HCl (15 ml, 0.2 M) and diluted with 60 ml of DCM. The organic layer was separated and the aqueous layer was extracted with DCM (3×20 ml). The combined organic layers were washed with brine (40 ml) and all solvents were removed under reduced pressure at 30 °C to give the crude product. Purification was performed by reversed phase MPLC using a water gradient in acetonitrile as eluent (see Figure S28). After the collection of unreacted educt compound **6$^{\bullet}$** and an impurity fraction, the main product fraction (37 mg) was eluted at 10% v/v water in acetonitrile. MS-MALDI(+) showed that this fraction contained target compound **4a$^{\bullet\bullet}$** as well as sulfoxide derivatives thereof (Δ *m/z* = 16, see Figure S29). The column was rinsed with THF to collect small amounts of non-oxidized product compound in a mixture with two impurities. This mixture was put to silica gel column chromatography using 1% v/v acetonitrile in chloroform as eluent. Compound **4a$^{\bullet\bullet}$** was obtained as a brown solid (3.00 mg, 1.36 µmol, 6%). The compound´s purity was additionally assessed by MPLC (see Figure S31).

MS (MALDI+) *m/z*: 2205.8 (100; $[C_{118}H_{106}O_{12}S_{24}]^{\bullet\bullet+}$; $[M+H]^{\bullet\bullet+}$), 1195.2 (11; $[C_{54}H_{51}O_7S_{12}]^{\bullet+}$; bridging molecule **8** with one trityl **6$^{\bullet}$**).

1.9 Synthesis of bis-trityl compound ***4b••***.

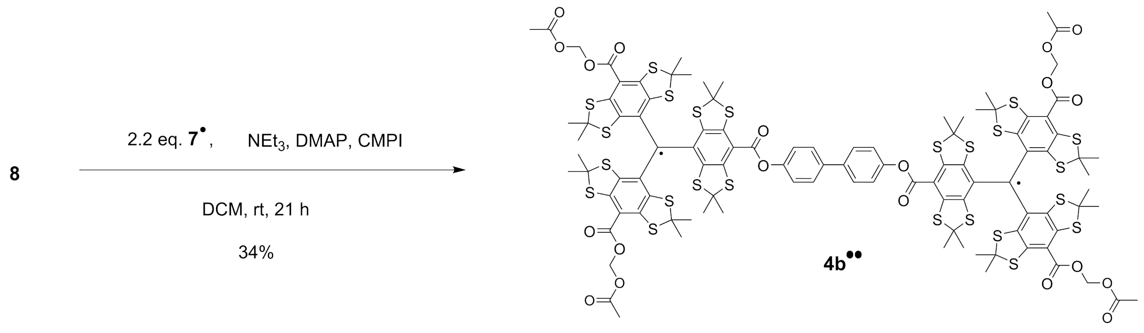

Compound **4b••** was synthesized following a modified procedure by Saigo *et al*.[2]

Compound **7•** (33.0 mg, 28.8 µmol) as well as the reactants 2-chloromethylpyridinium iodide (CMPI, 8.84 mg, 34.6 µmol) and 4-dimethyl- aminopyridine (DMAP, 1.41 mg, 11.5 µmol) were dried under reduced pressure for two hours before starting the reaction. Dry dichloromethane (DCM, 3 ml) and triethylamine (9.80 µL, 69.4 µmol) were degassed together via three freeze-pump-thaw cycles.
Under an argon atmosphere, compound **7•** was dissolved in the degassed mixture of DCM and triethylamine, and stirred at room temperature for two hours to achieve complete deprotonation of the carboxylic acid groups. After addition of 4,4′-Dihydroxybiphenyl (2.42 mg, 13.0 µmol), the brown reaction mixture was cooled down to 0 °C before CMPI and DMAP were added. The mixture was stirred at room temperature for 21 hours and then quenched with aqueous HCl (10 ml, 0.3 M). The organic layer was separated and the aqueous layer was extracted with DCM (3×10 ml). The combined organic layers were washed with brine (15 ml) and all solvents were removed under reduced pressure at 30 °C to give the crude product. Purification was performed by silica column chromatography using chloroform followed by 1.5% v/v methanol in chloroform as eluents. Purification attempts using the GPC yielded sulfoxide derivatives of the target compound (see Figures S35 and S36), just as observed for the MPLC purification of compound **4a••** (see section

1.8). Product compound **4b**$^{\bullet\bullet}$ was obtained as a brown solid (10.6 mg, 4.35 µmol, 34%). The compound´s purity was additionally assessed by MPLC (see Figure S34).

MS (MALDI+) *m/z*: 2436.1 (100; $[C_{104}H_{100}O_{20}S_{24}]^{\bullet\bullet+}$; $[M]^{\bullet\bullet+}$), 1311.1 (11; $[C_{58}H_{55}O_{11}S_{12}]^{\bullet+}$; bridging molecule **8** with one trityl **7**$^{\bullet}$).
HRMS (ESI) *m/z*: $[M+Na]^{\bullet\bullet+}$ calcd. for $[C_{104}H_{100}O_{20}S_{24}Na]^{\bullet\bullet+}$: 2458.9997, found: 2458.9993.

2. NMR, Mass Spectrometry and Chromatography Data

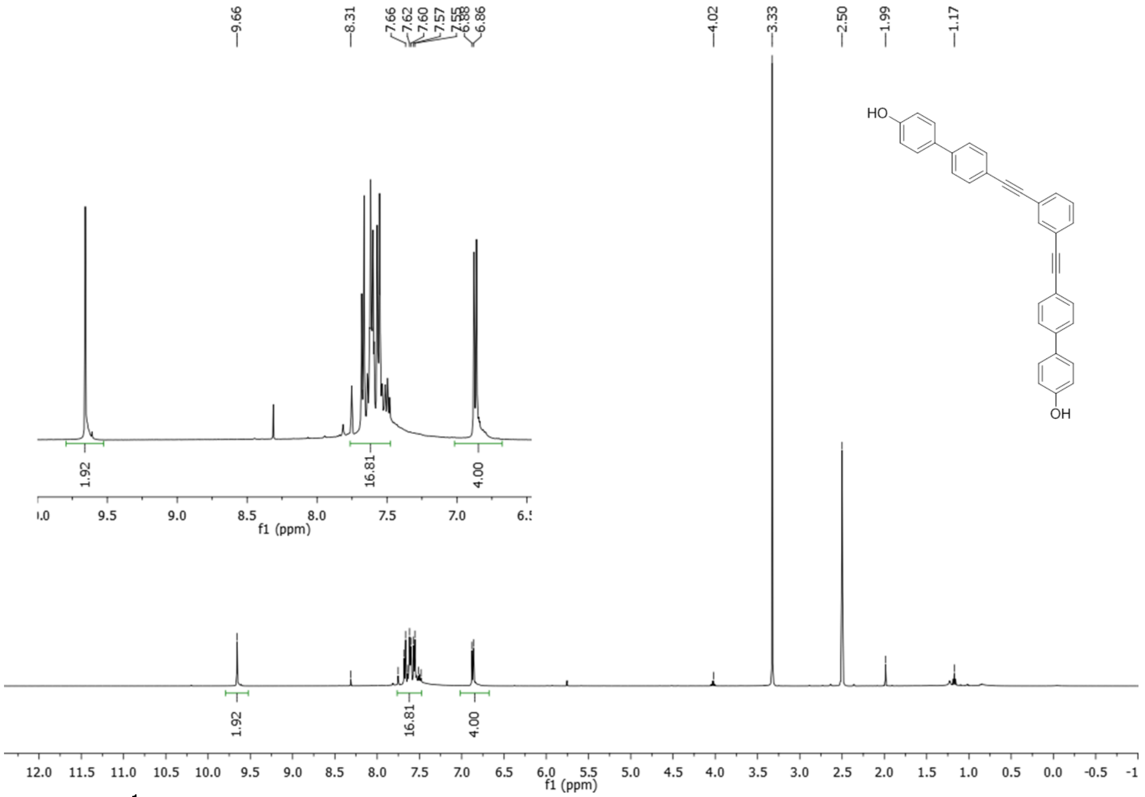

Figure S1: ^{1}H-NMR spectrum of compound **9** in deuterated DMSO.

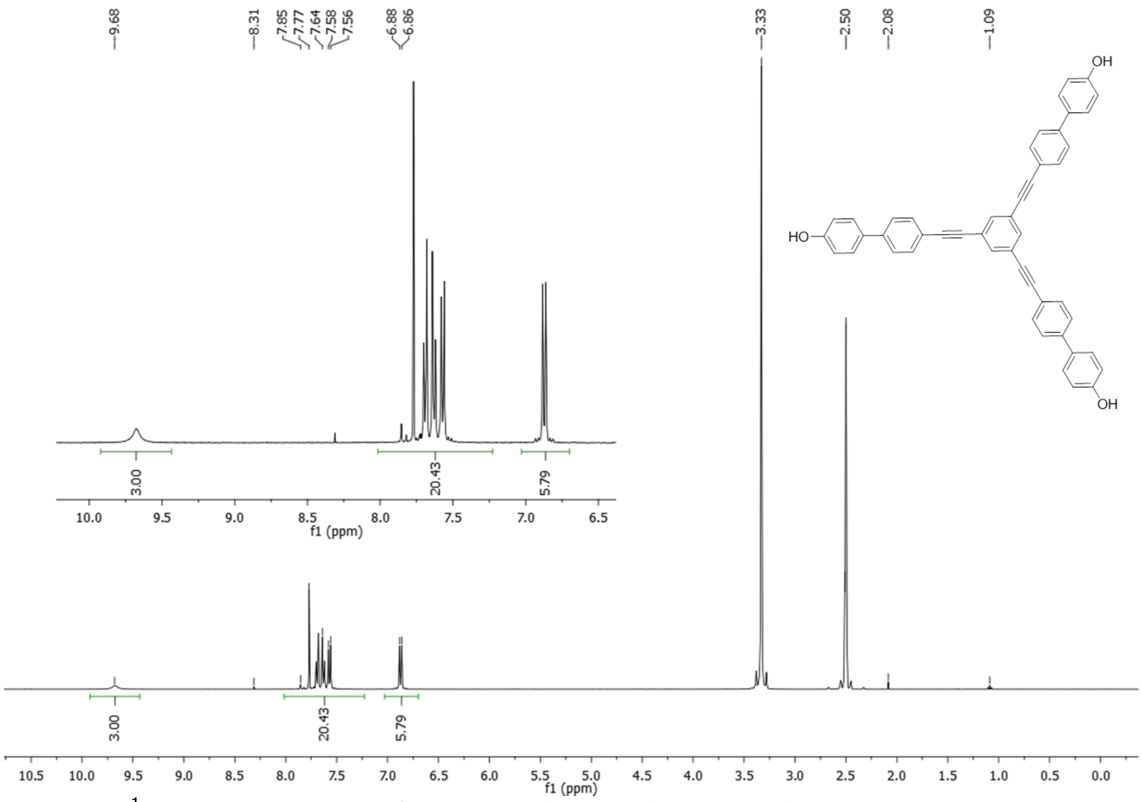

Figure S2: ^{1}H-NMR spectrum of compound **10** in deuterated DMSO.

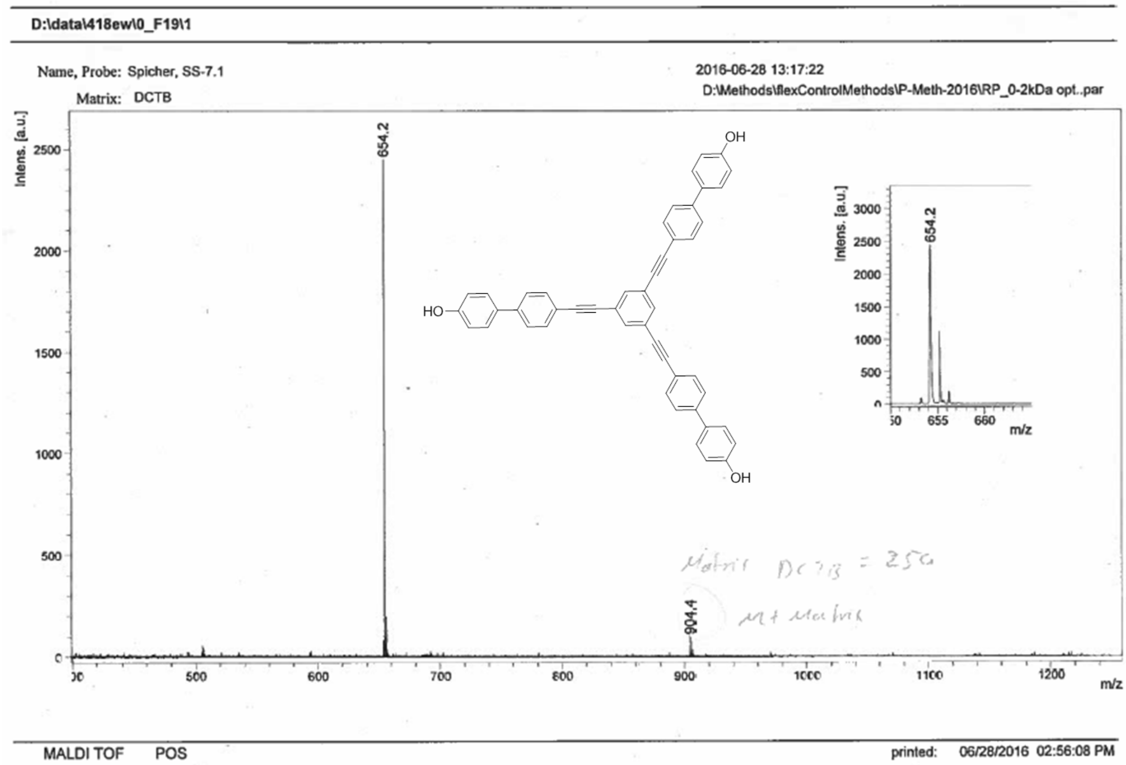

Figure S3: MS-MALDI(+) spectrum of compound **10.**

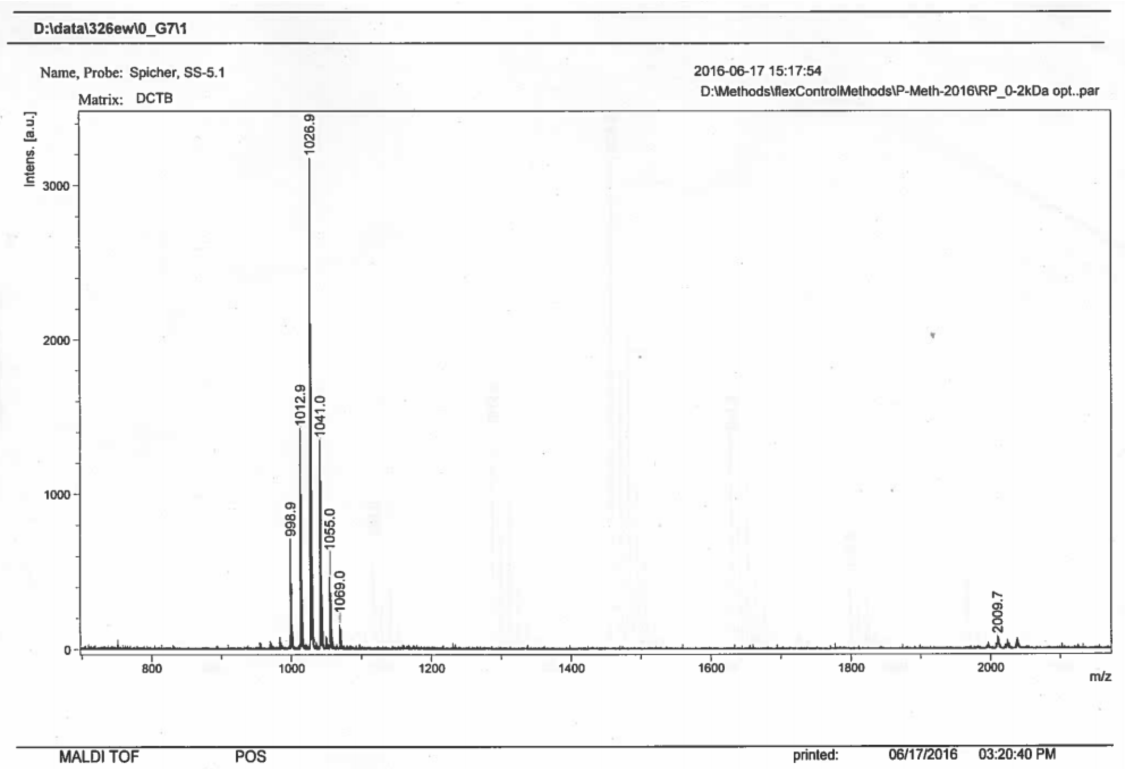

Figure S4: MS-MALDI(+) spectrum of the product mixture leading to compound **5•**.

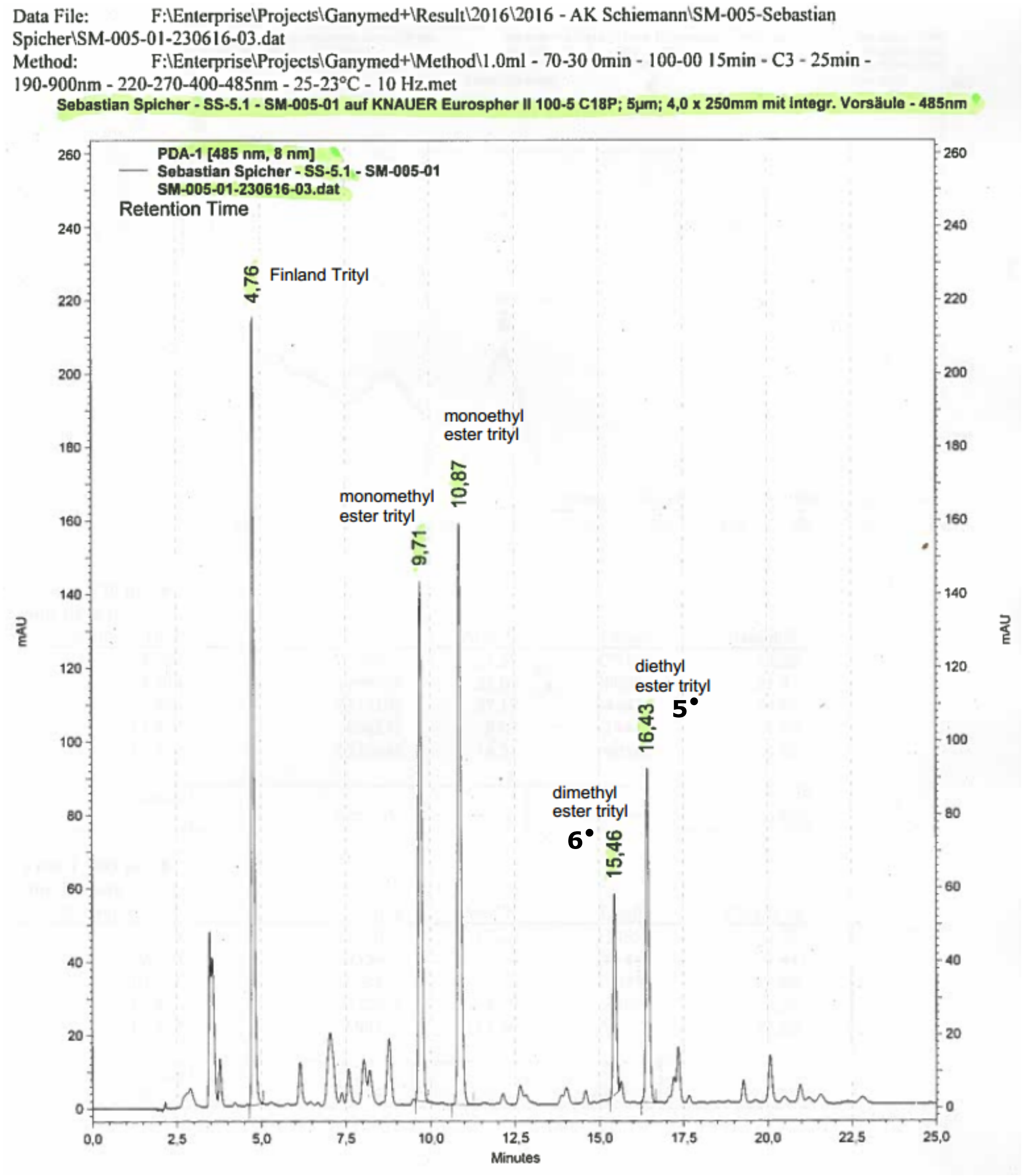

Figure S5: HPLC elugram for the product mixture leading to compounds **5•** and **6•**.

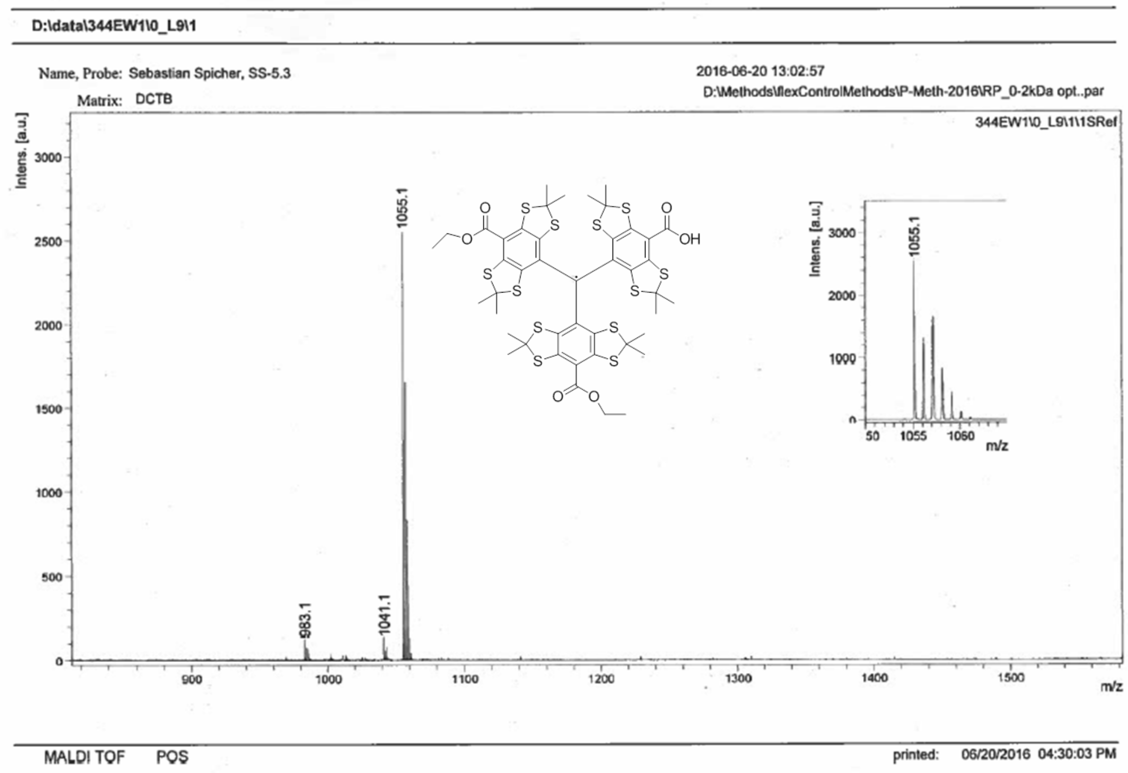

Figure S6: MS-MALDI(+) spectrum of compound **5•**.

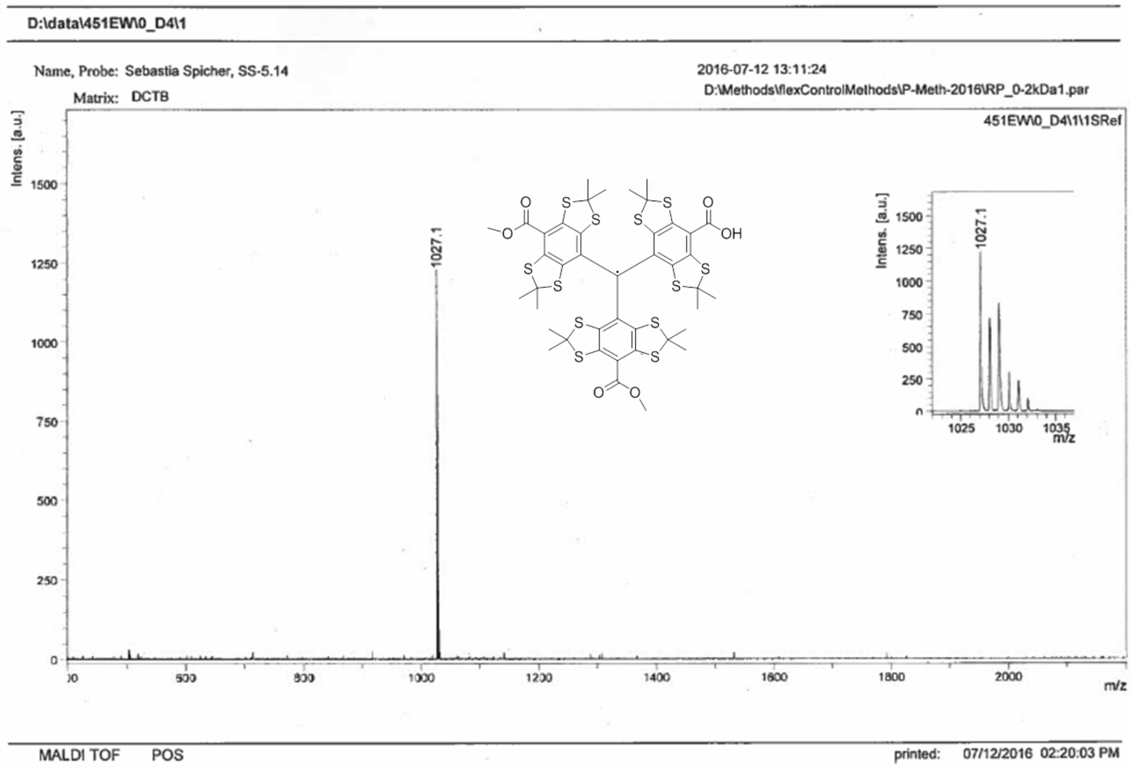

Figure S7: MS-MALDI(+) spectrum of compound **6•** separated from **5•**.

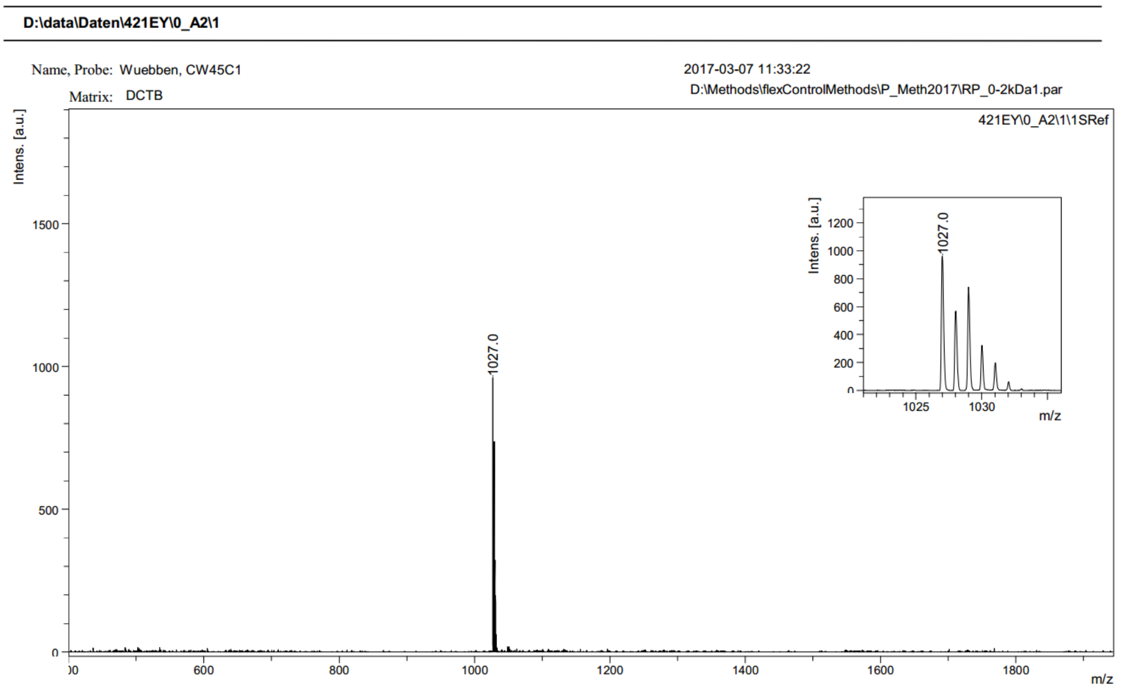

Figure S8: MS-MALDI(+) spectrum of compound **6•**.

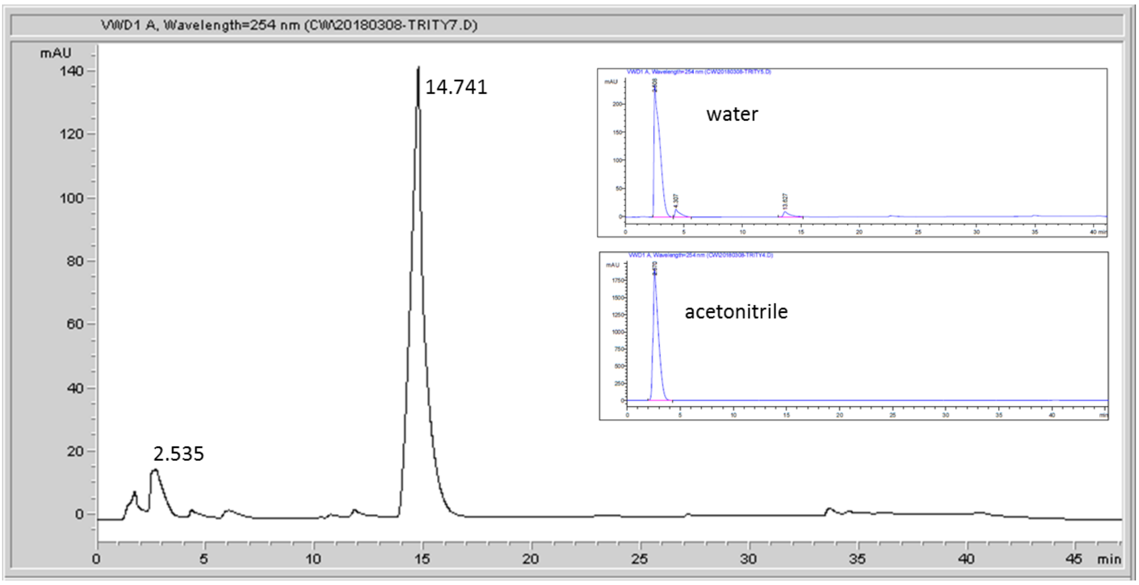

Figure S9: HPLC purity assesment for compound **6•**. The detections at min 1—4 are caused by the eluent components (see inlets).

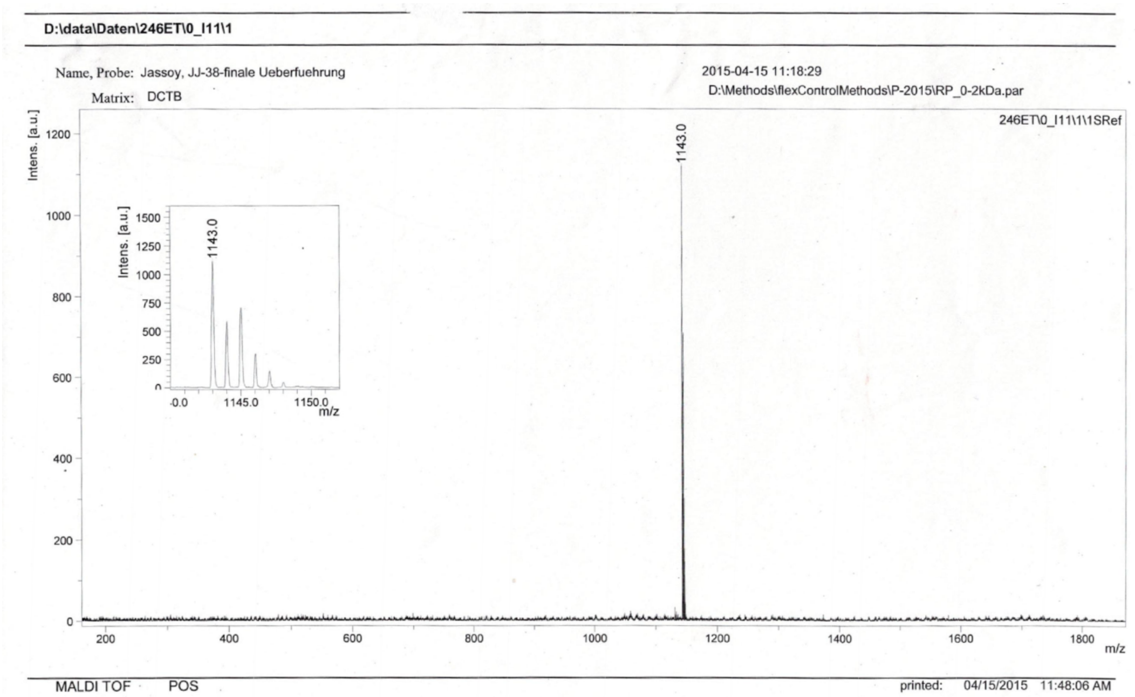

Figure S10: MS-MALDI(+) spectrum of compound **7•**.

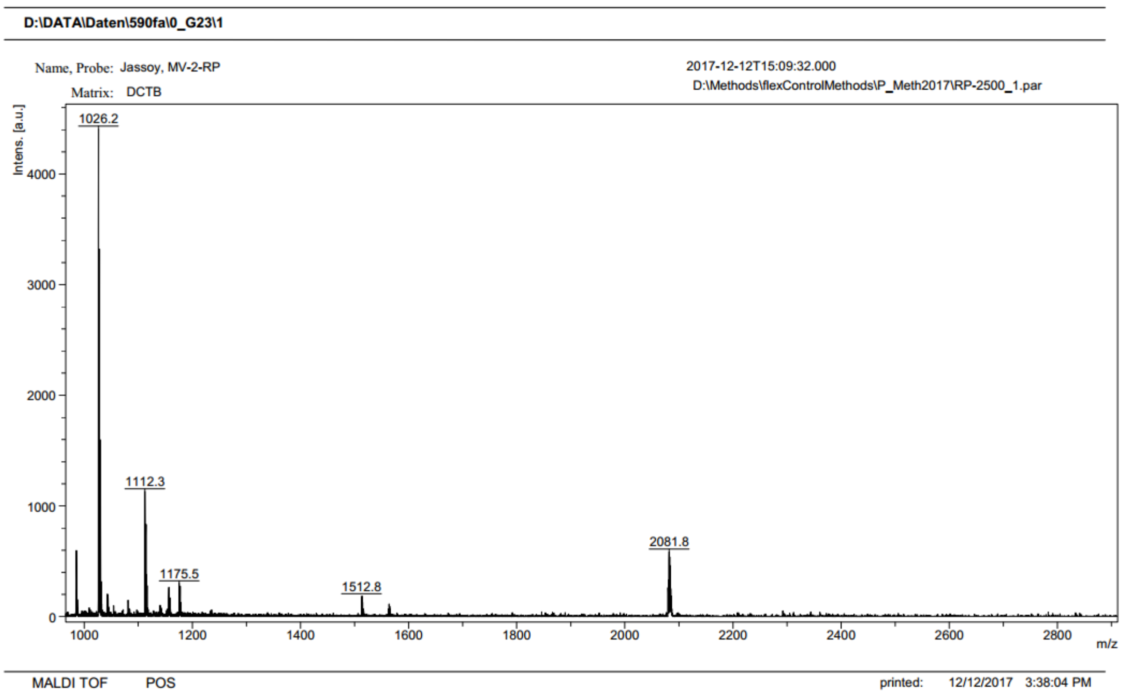

Figure S11: MS-MALDI(+) spectrum of crude product for the reaction to **2a••**.

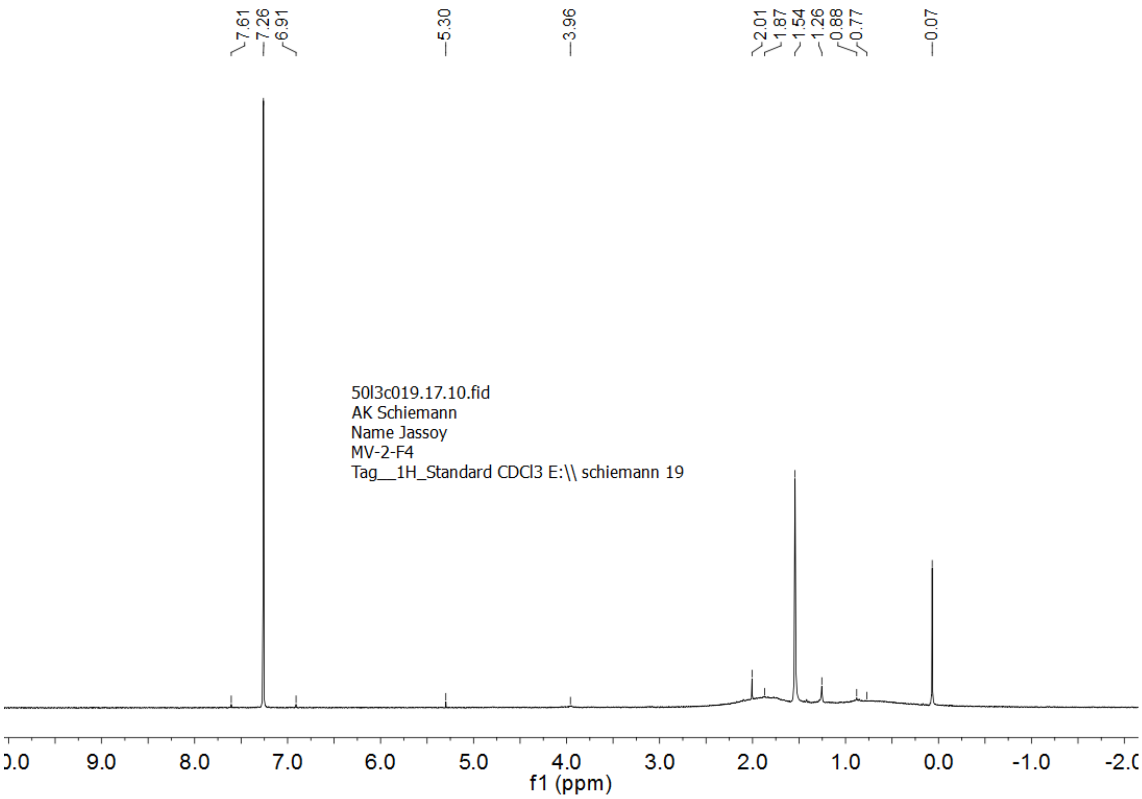

Figure S12: ^{1}H-NMR spectrum of isolated by-product (*m/z* = 2081) of the reaction towards **2a••** in $CDCl_3$.

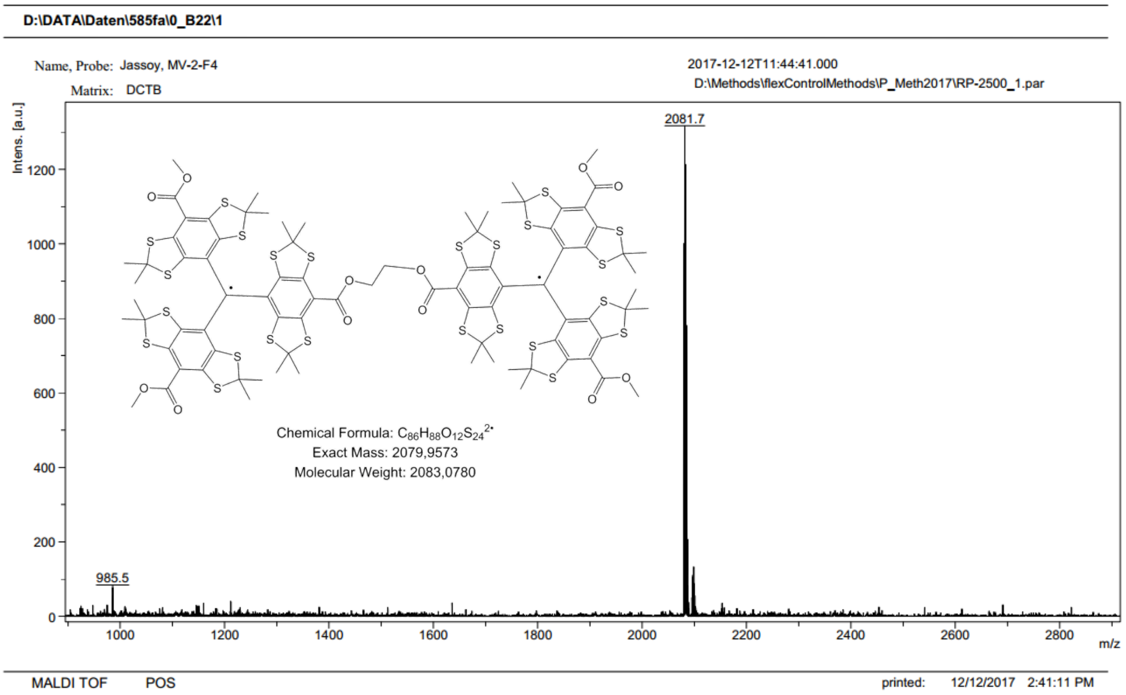

Figure S13: MS-MALDI(+) spectrum of isolated by-product (*m/z* = 2081) of the reaction to **2a••**.

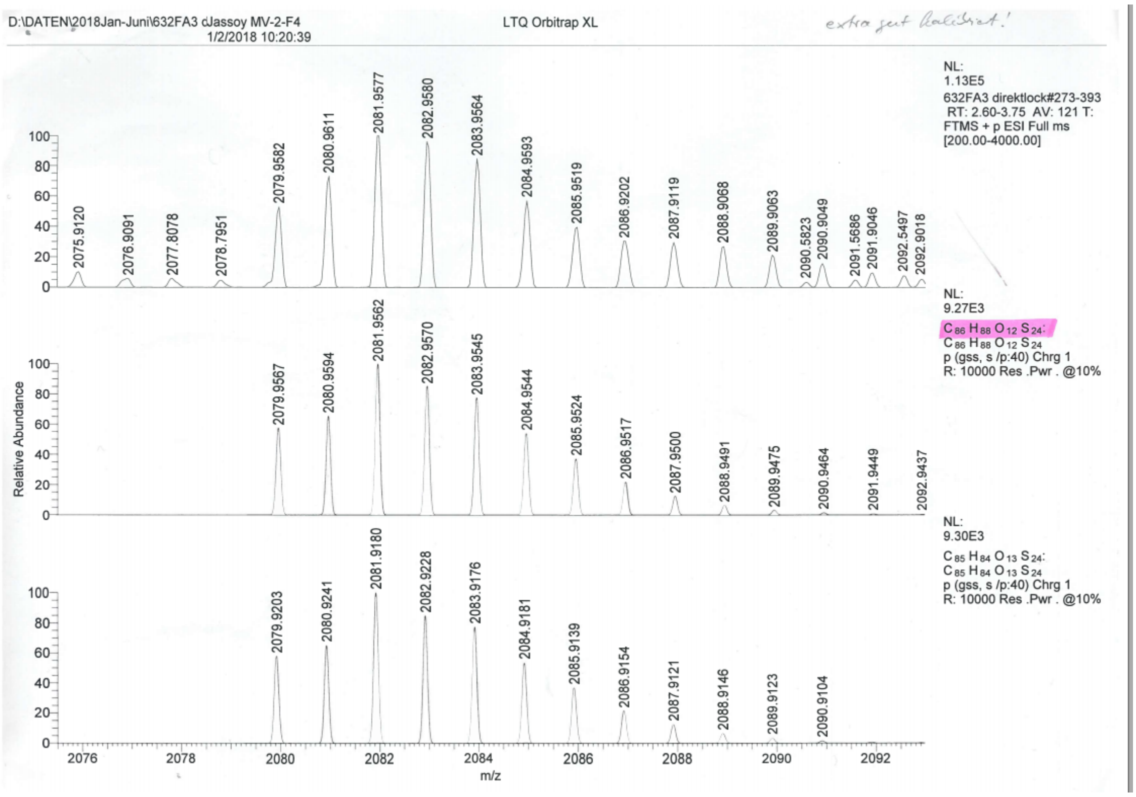

Figure S14: HRMS-APCI spectrum of isolated by-product (m/z = 2081) of the reaction to **2a**$^{\bullet\bullet}$.

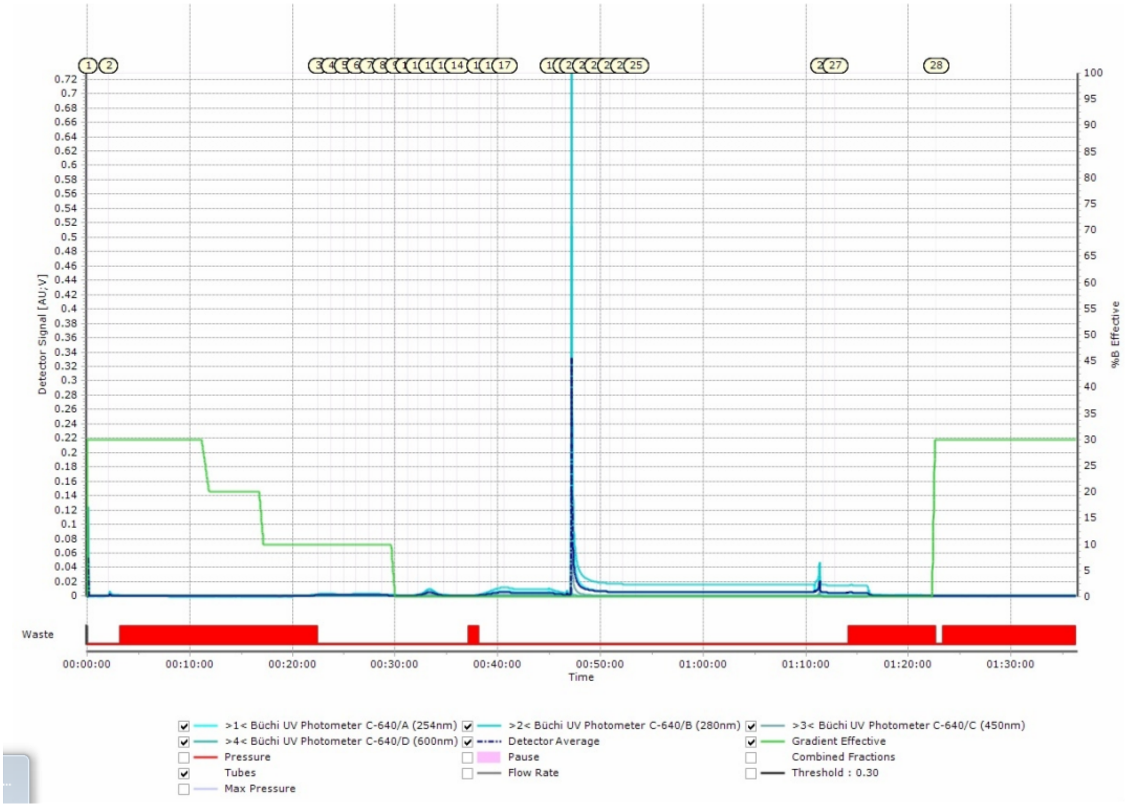

Figure S15: MPLC purification elugram for compound **2a**$^{\bullet\bullet}$. A gradient of acetonitrile in water followed by pure THF (t = 48 min) was used. Fraction 20 gave the product compound.

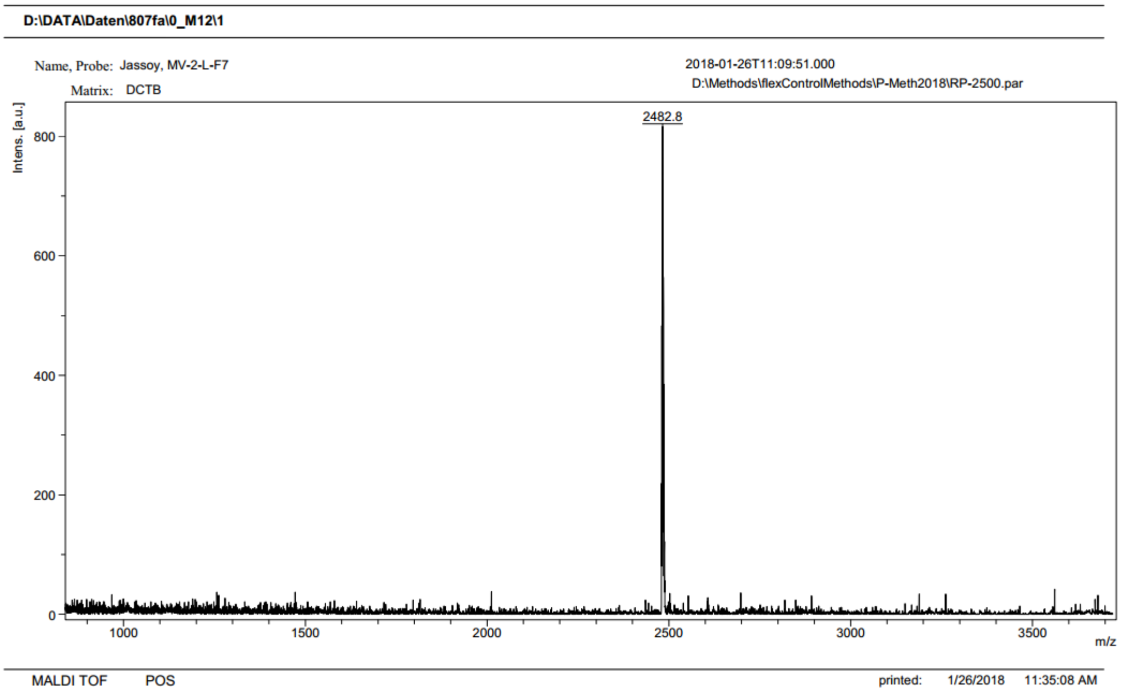

Figure S16: MS-MALDI(+) spectrum of compound **2a••**.

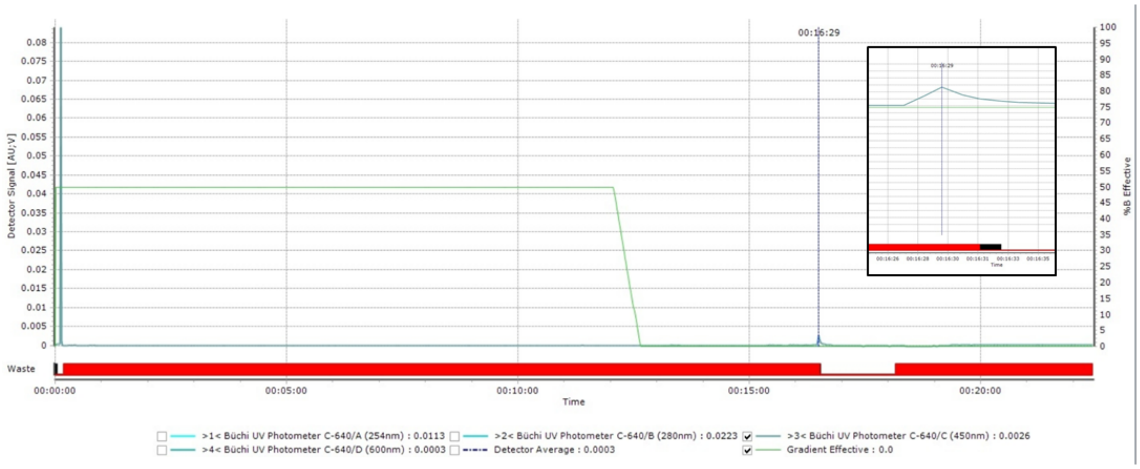

Figure S17: MPLC purity assessment for compound **2a••** (time~16:40 min). A gradient of acetonitrile in water followed by THF was applied. The conventional detection wavelength of 254 nm is omitted, because the solvent change towards THF caused misleading absorptions at this wavelength (see Figure S18). Instead, the detection at 450 nm is displayed, which gives a considerably weaker signal. The same applies for model compounds **2b••** and **3a•••** which also need to be eluted via solvent change towards THF.

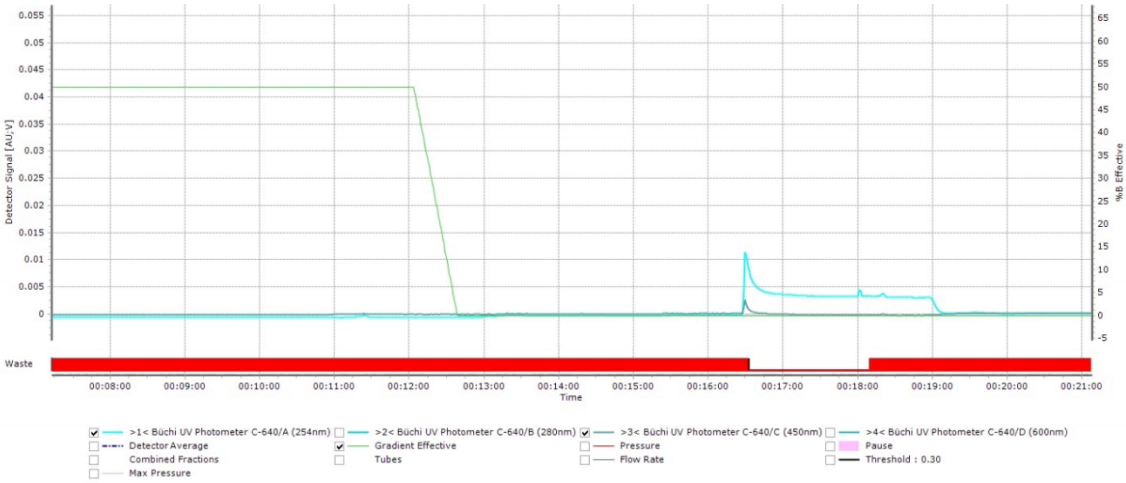

Figure S18: MPLC purity assessment for compound **2a**$^{\bullet\bullet}$ including the detection at 254 nm (strong) aside from 450 nm (weak). In order to check the effect of solvent change towards THF on the MPLC detection signals, a sample-free elution run was performed (see Figure S19).

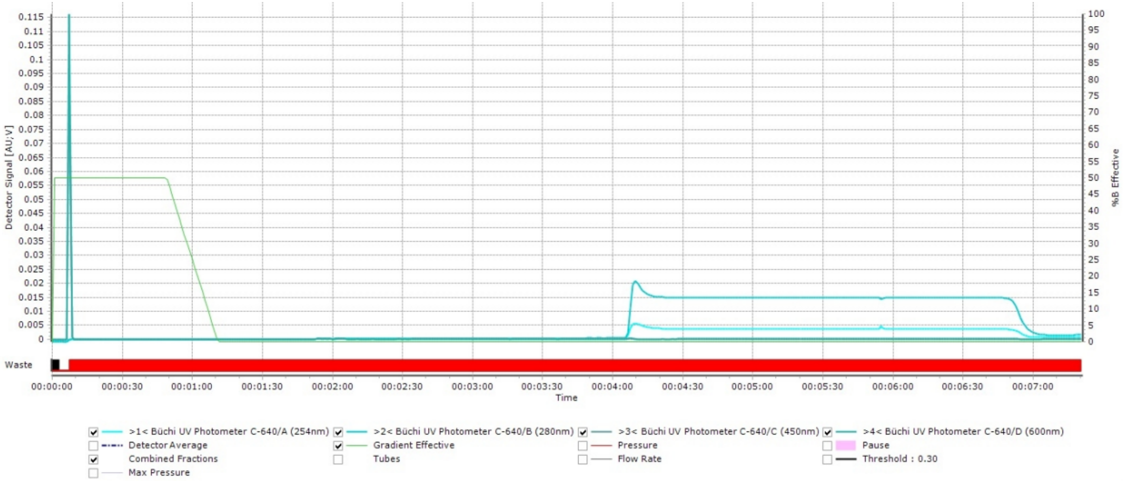

Figure S19: A sample-free elution run with eluent change from water/acetonitrile towards THF at time~3 min. The solvent change becomes manifest in the detection channels at 254 (cyan) and 280 nm (petrol).

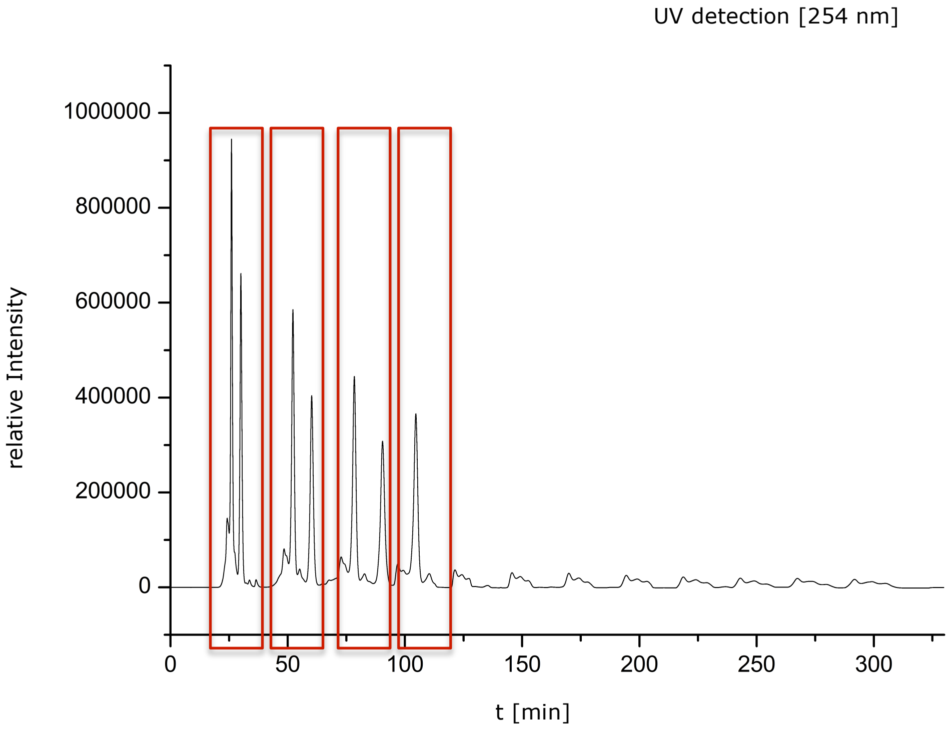

Figure S20: GPC elugram for compound **2b**$^{\bullet\bullet}$. Three fractions are separated by their differing hydrodynamic radii. The first collection is performed during the third GPC cycling round (red framed boxes).

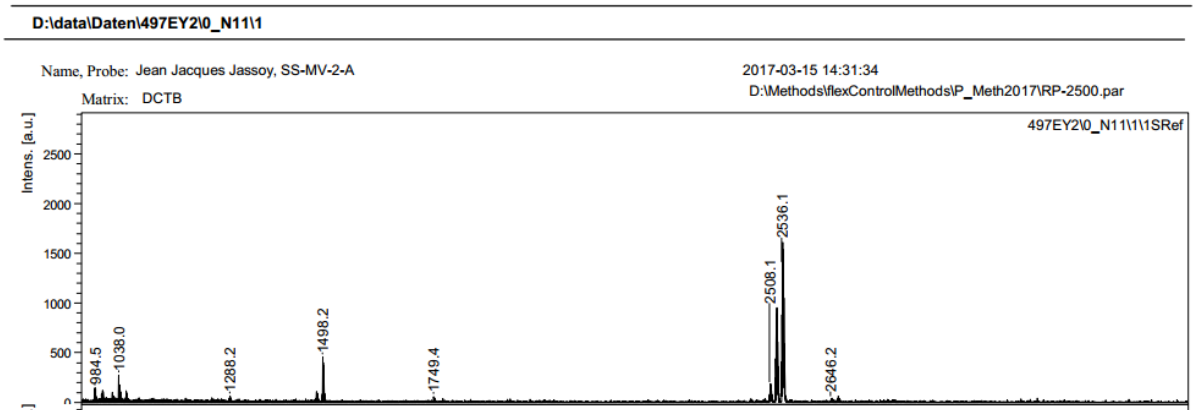

Figure S21: MS-MALDI(+) spectrum of compound **2b**$^{\bullet\bullet}$ after GPC and subsequent silica gel column chromatography.

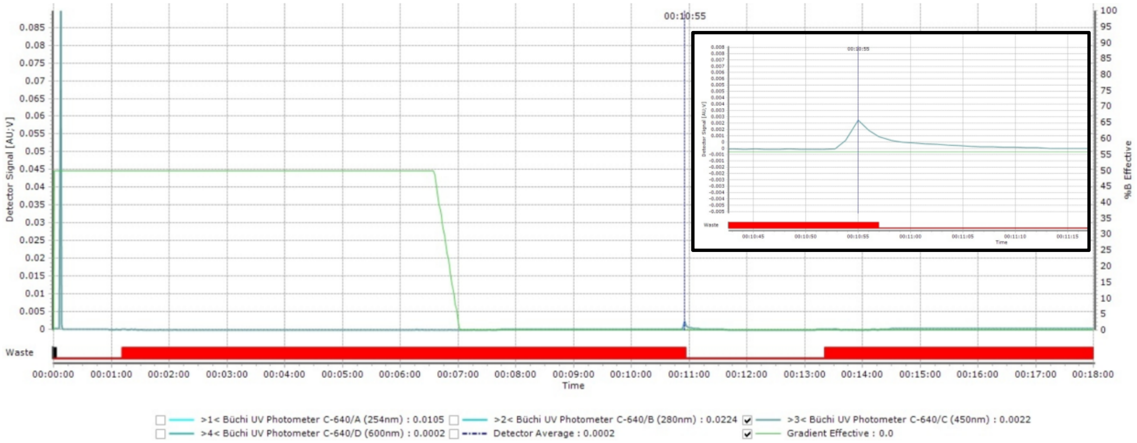

Figure S22: MPLC purity assessment for compound **2b••** (time~11 min). A gradient of acetonitrile in water followed by THF was applied.

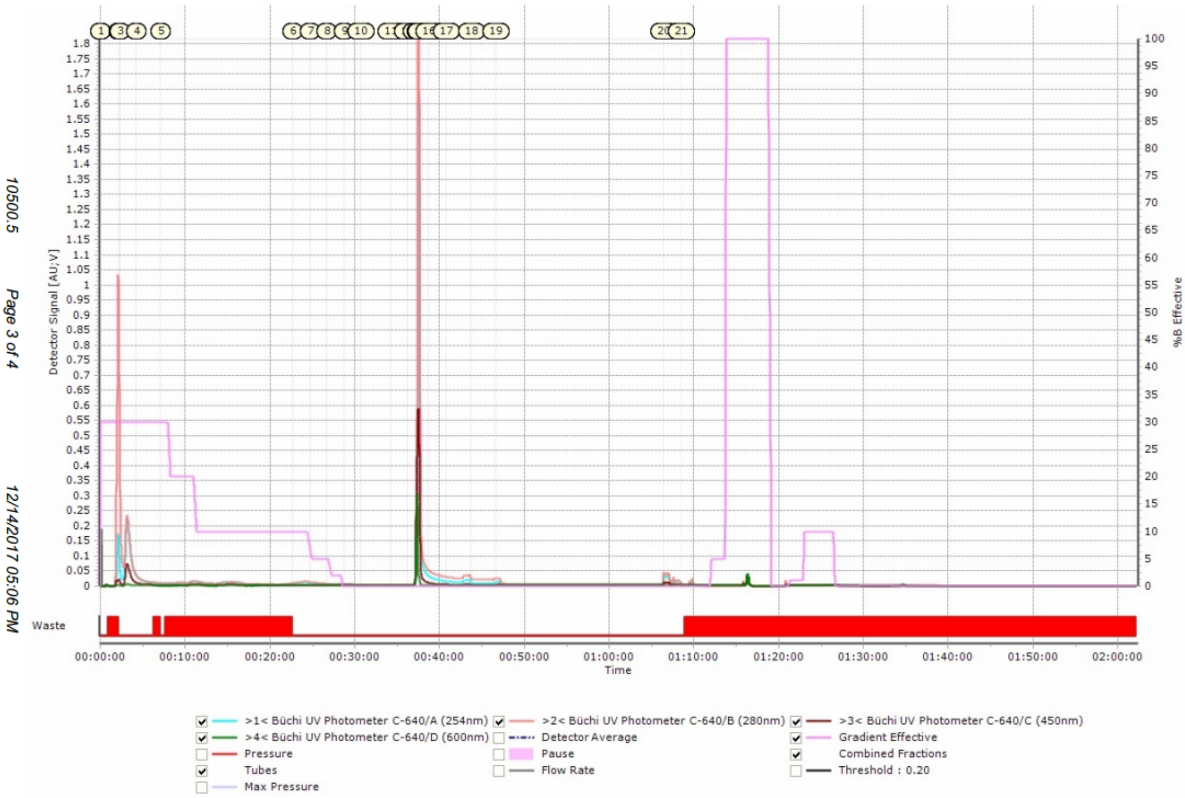

Figure S23: MPLC elugram for compound **3a•••**. Two main fractions are separated by their polarity against a reversed phase stationary phase. The target compound is collected with THF as eluent at time~37 minutes in the depictd elugram.

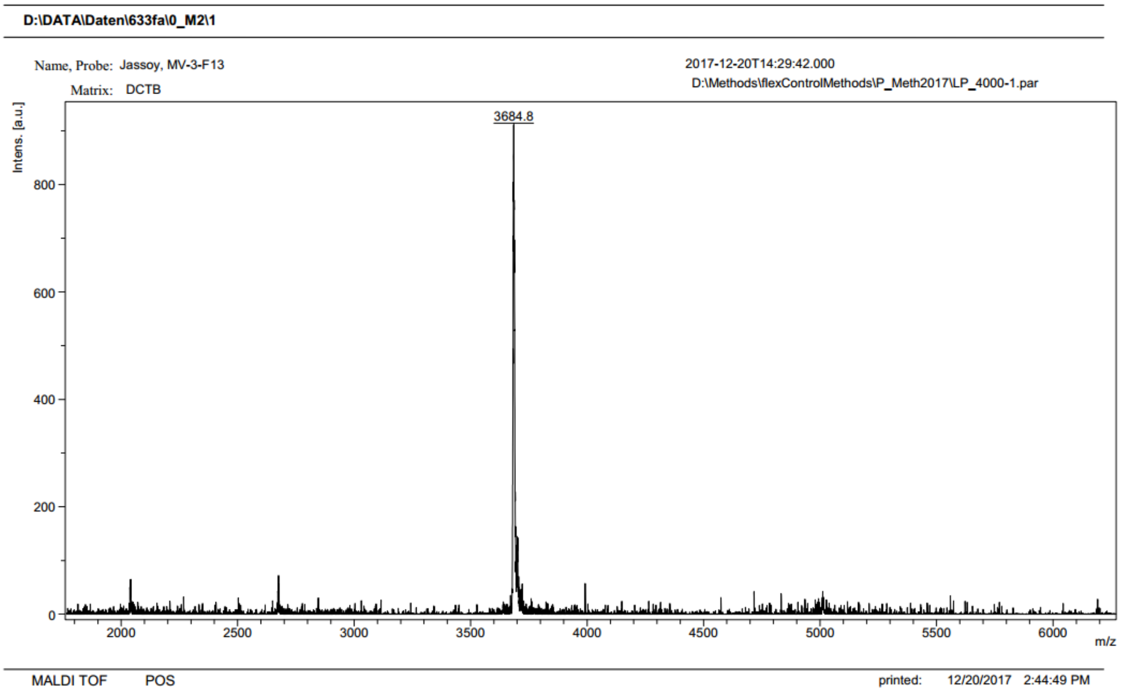

Figure S24: MS-MALDI(+) spectrum of compound **3a•••** for the reaction with THF as solvent.

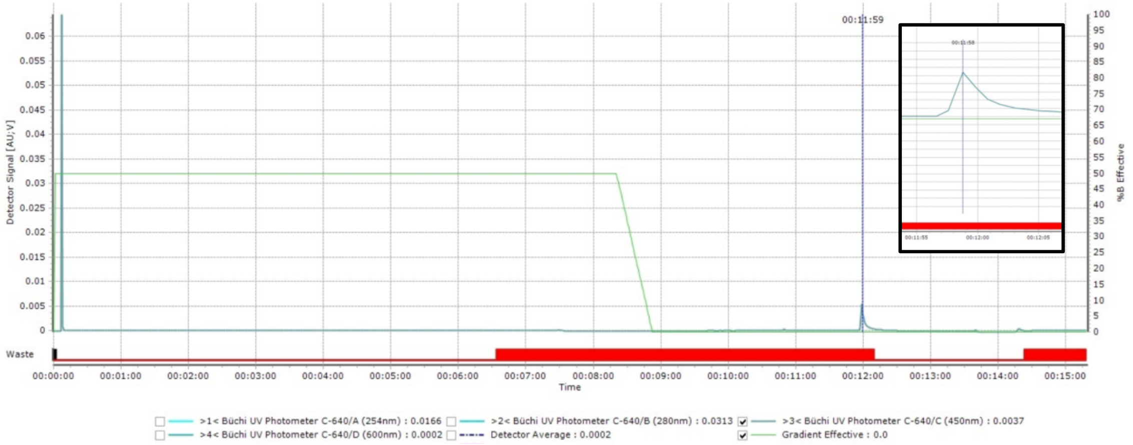

Figure S25: MPLC purity assessment for compound **3a•••** (time~12 min). A gradient of acetonitrile in water followed by THF was applied.

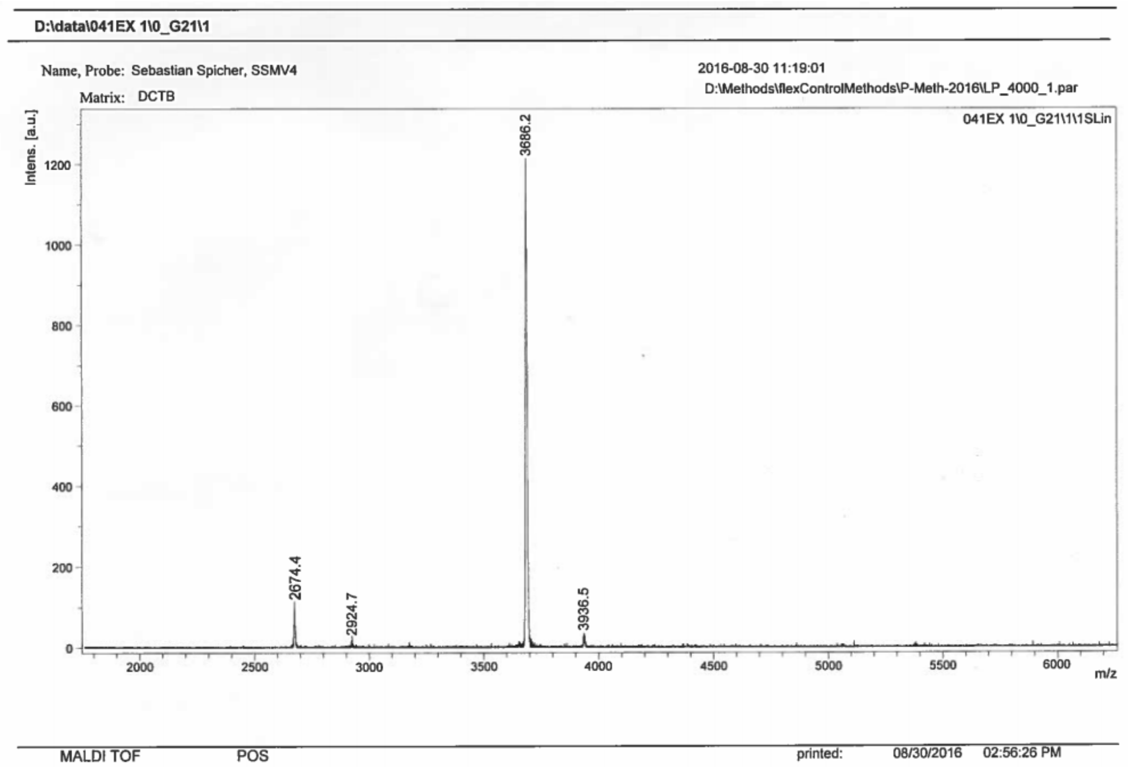

Figure S26: MS-MALDI(+) spectrum of compound **3a•••** for the reaction with THF/DCM (1/1) as solvent.

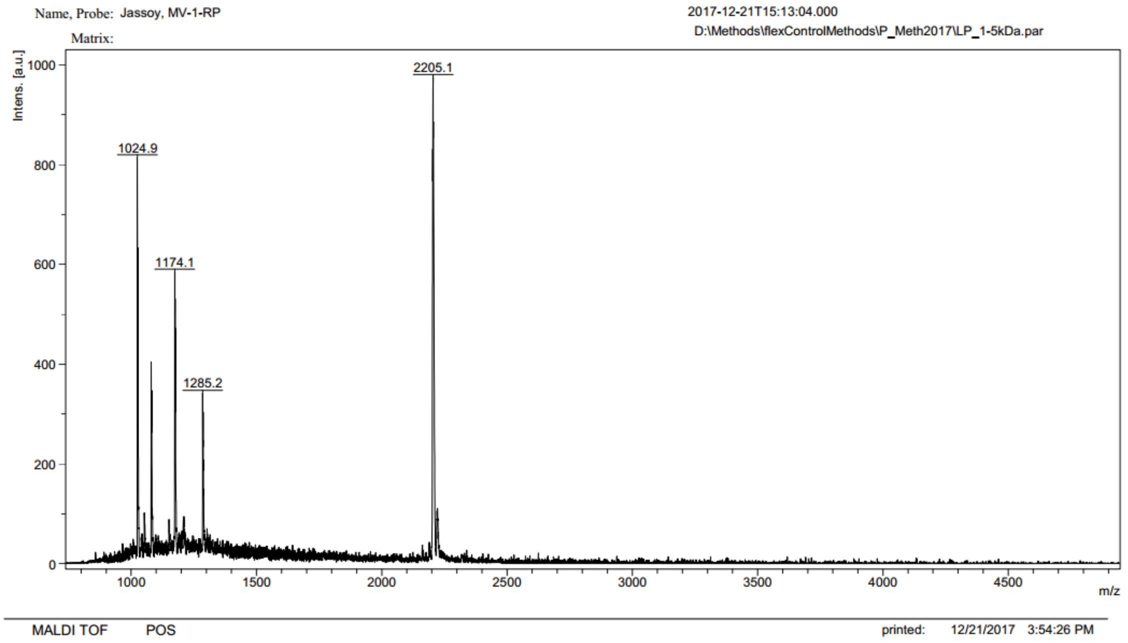

Figure S27: MS-MALDI(+) spectrum of the crude product for compound **4a••**.

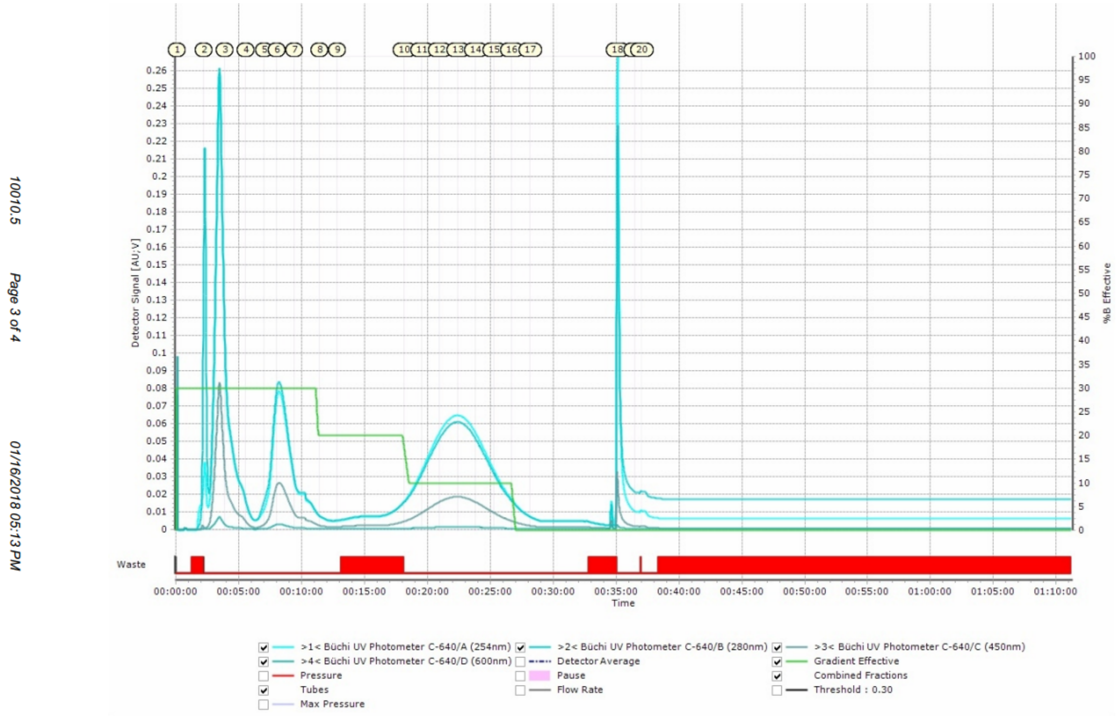

Figure S28: MPLC elugram for compound **4a••**. The mixed fraction of target compound and sulfoxide derivatives thereof is collected at time~17-29 minutes. The isolated target compound is collected at t ~35 minutes.

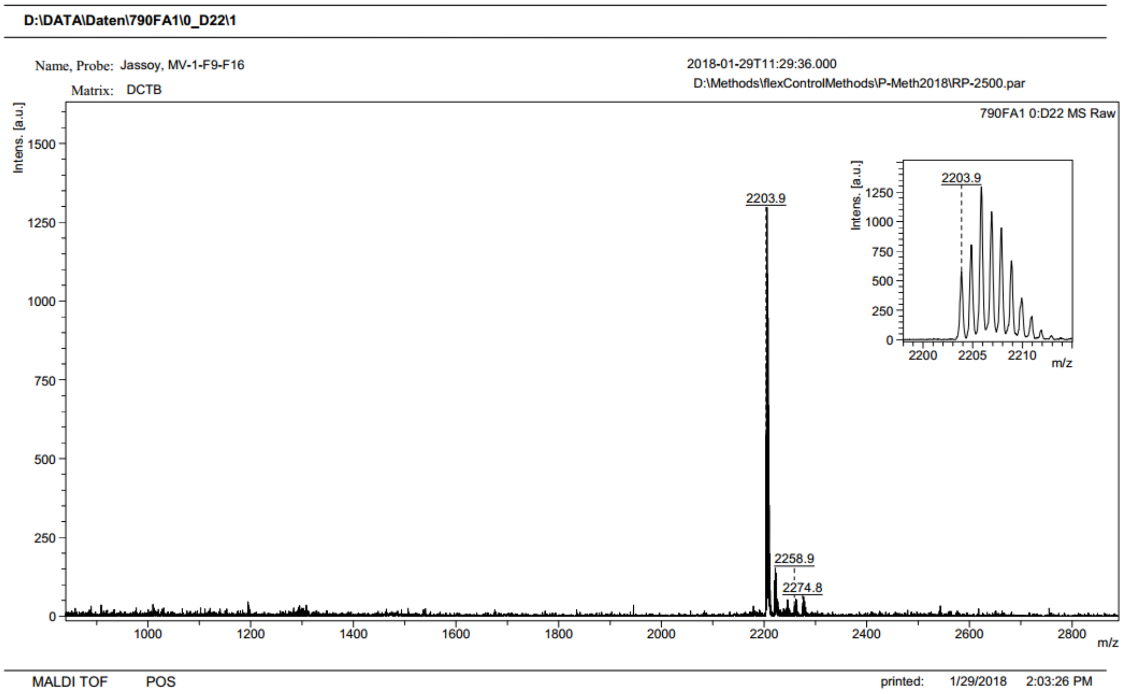

Figure S29: MS-MALDI(+) spectrum of compound **4a••** with sulfoxide derivative impurities. This sample is collected in fractions 9—16 during the MPLC procedure depicted in Figure S24.

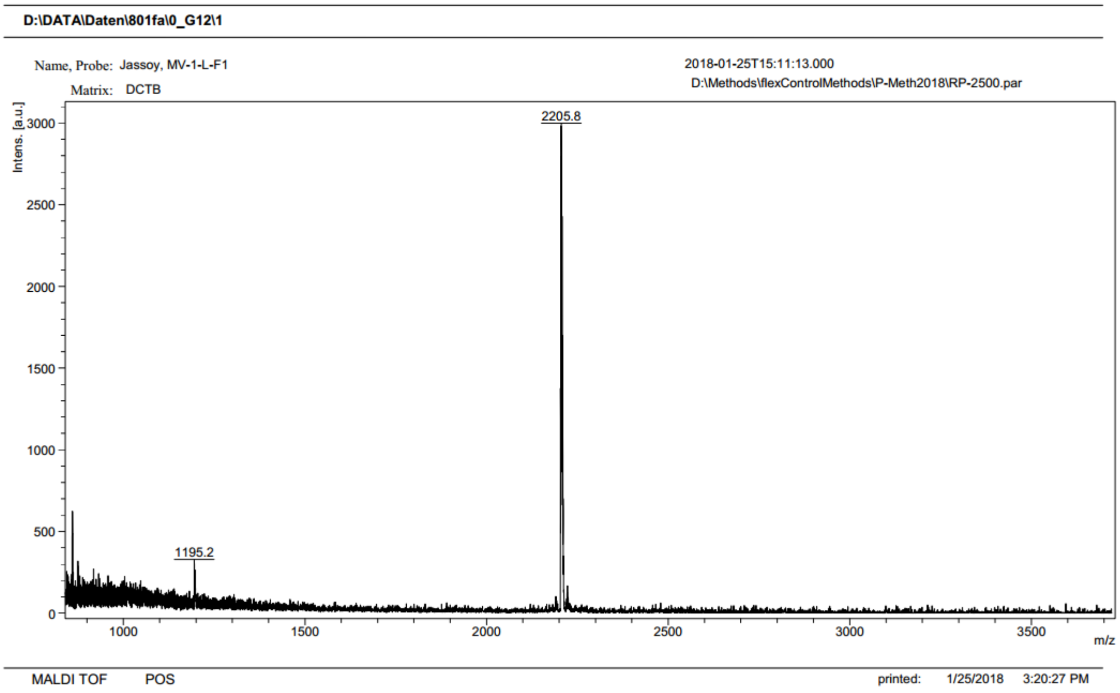

Figure S30: MS-MALDI(+) spectrum of compound **4a**$^{\bullet\bullet}$.

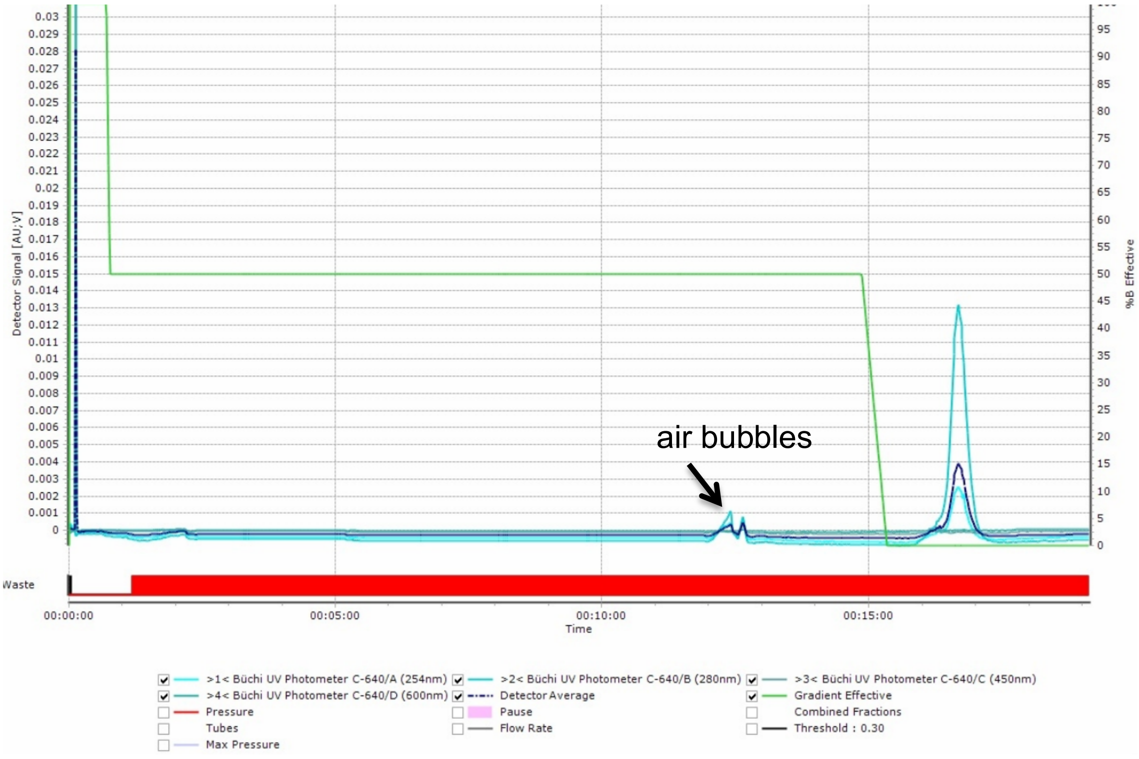

Figure S31: MPLC purity assessment for compound **4a**$^{\bullet\bullet}$ (time~17 min). A gradient of acetonitrile in water was applied.

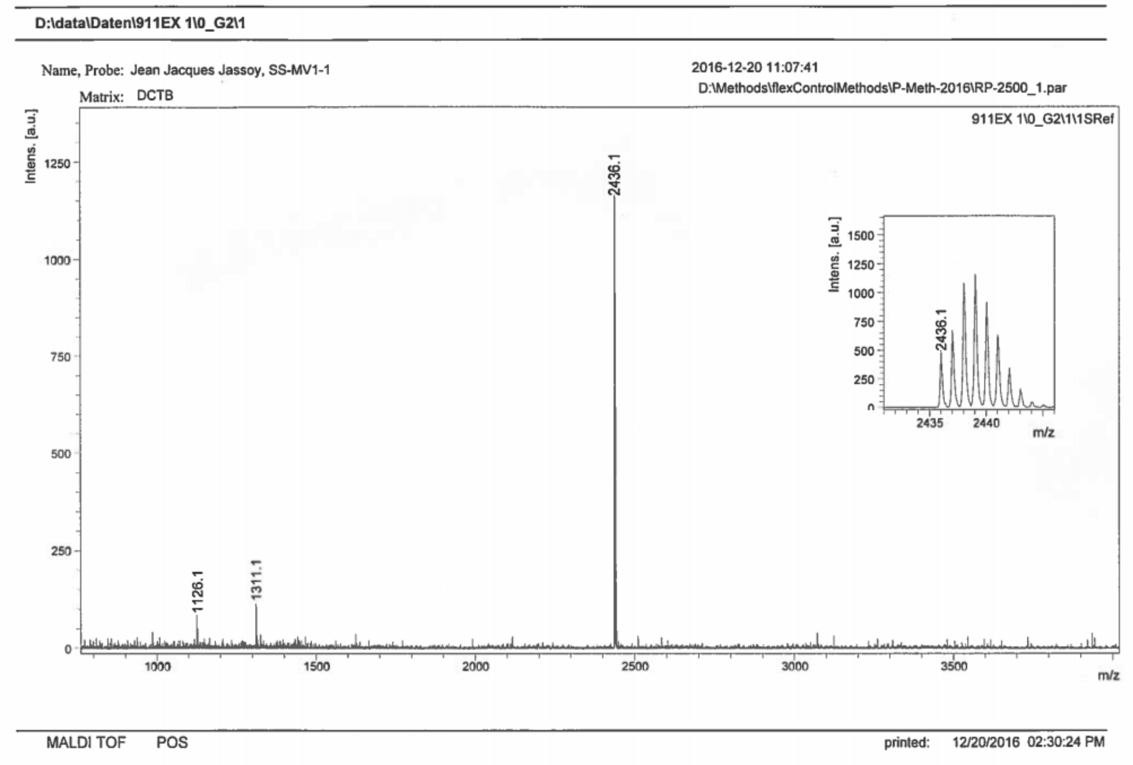

Figure S32: MS-MALDI(+) spectrum of compound **4b••**.

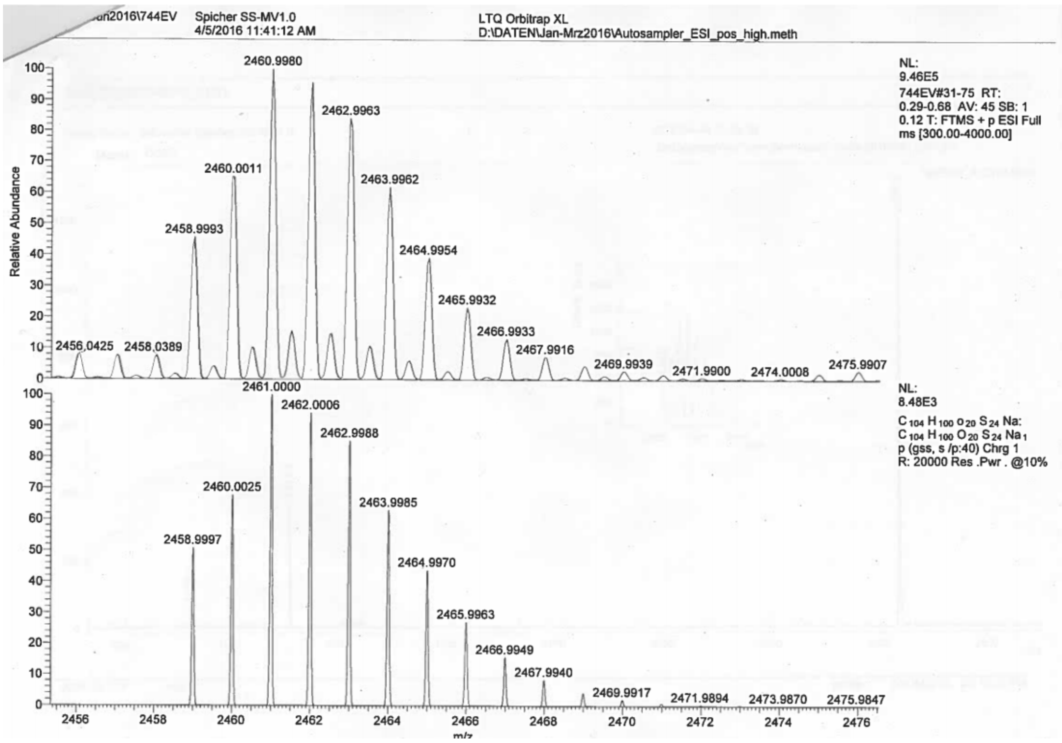

Figure S33: HRMS-ESI(+) spectrum of compound **4b••**.

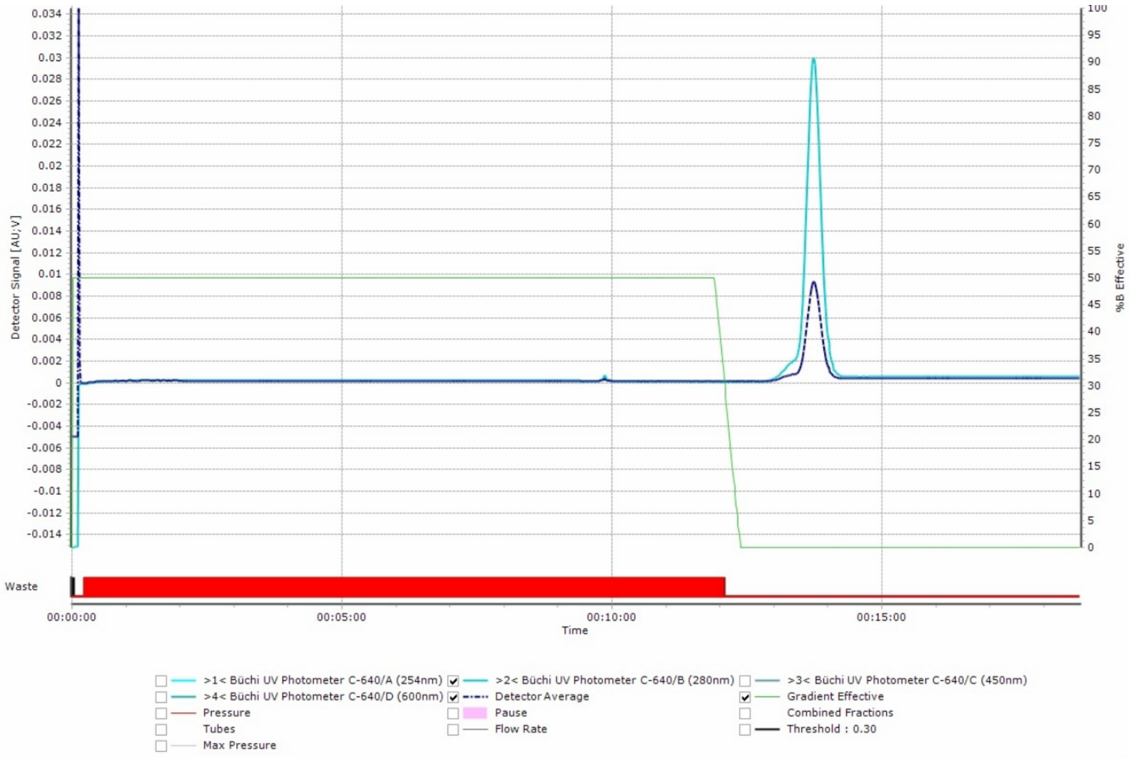

Figure S34: MPLC purity assessment for compound **4b••** (time~14 min). A gradient of acetonitrile in water was applied.

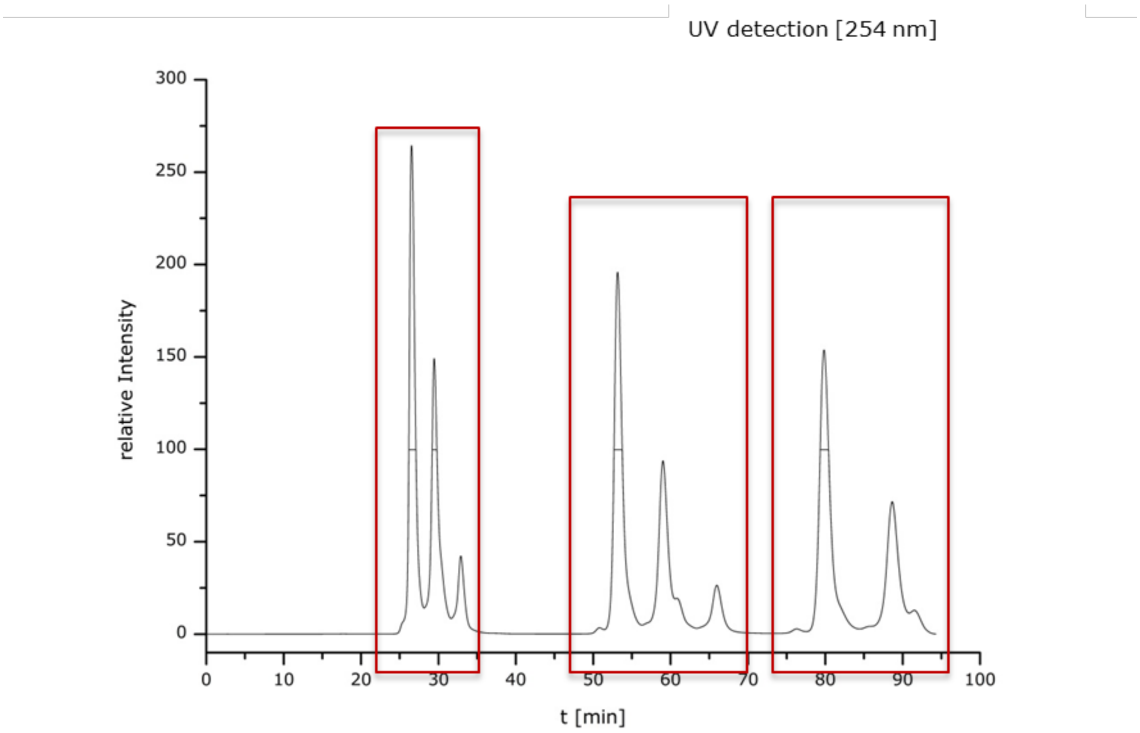

Figure S35: Exemplary GPC elugram for another synthesis of compound **4b••**. Three fractions are separated by their differing hydrodynamic radii. The first collection is performed during the third GPC cycling round (red framed boxes).

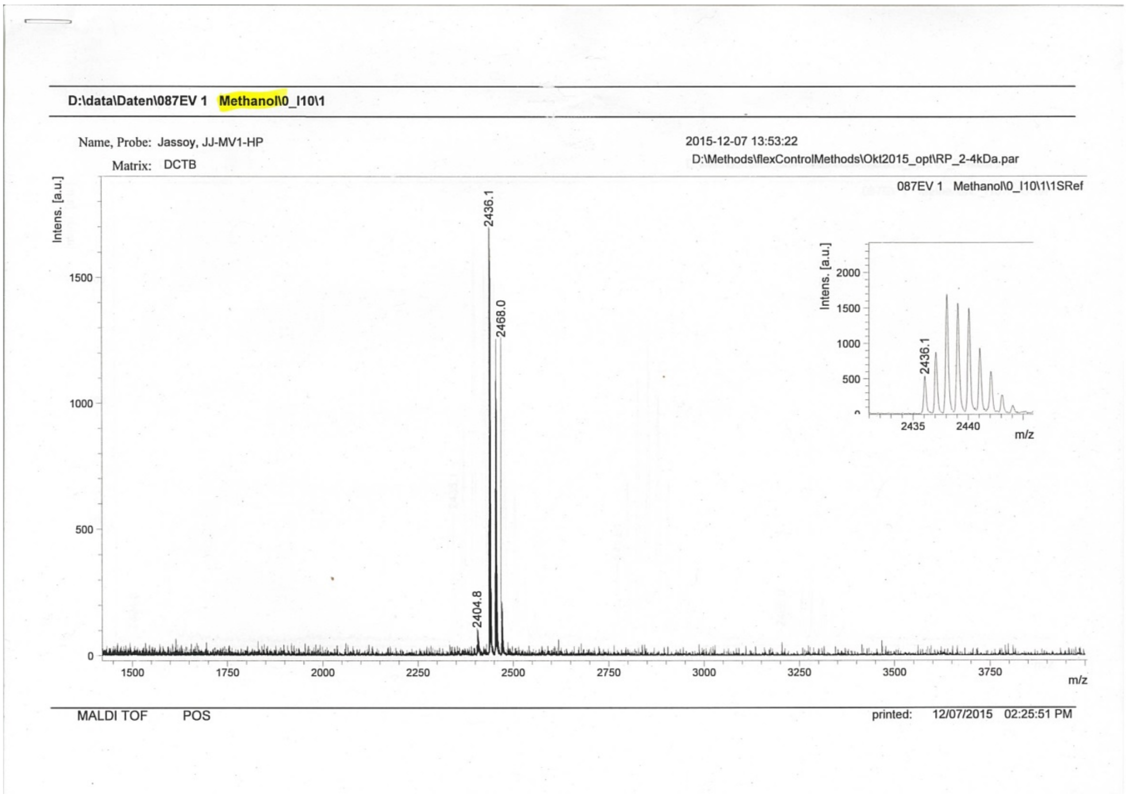

Figure S36: MS-MALDI(+) spectrum of compound **4b**$^{\bullet\bullet}$ with sulfoxide derivative impurities after GPC purification (see Figure S29 above).

3. Wavefunctions and energies in the different coupling regimes

3.1 Isotropic interactions

The isotropic case is of importance for the treatment of the exchange interaction observed in the room temperature cw EPR spectra. There, the exchange coupling constant *J* can be obtained by measuring the effective isotropic hyperfine coupling constant of the ^{13}C satellites. This means that the molecules of interest contain one ^{13}C atom in one of the two trityl units (Figure S37).

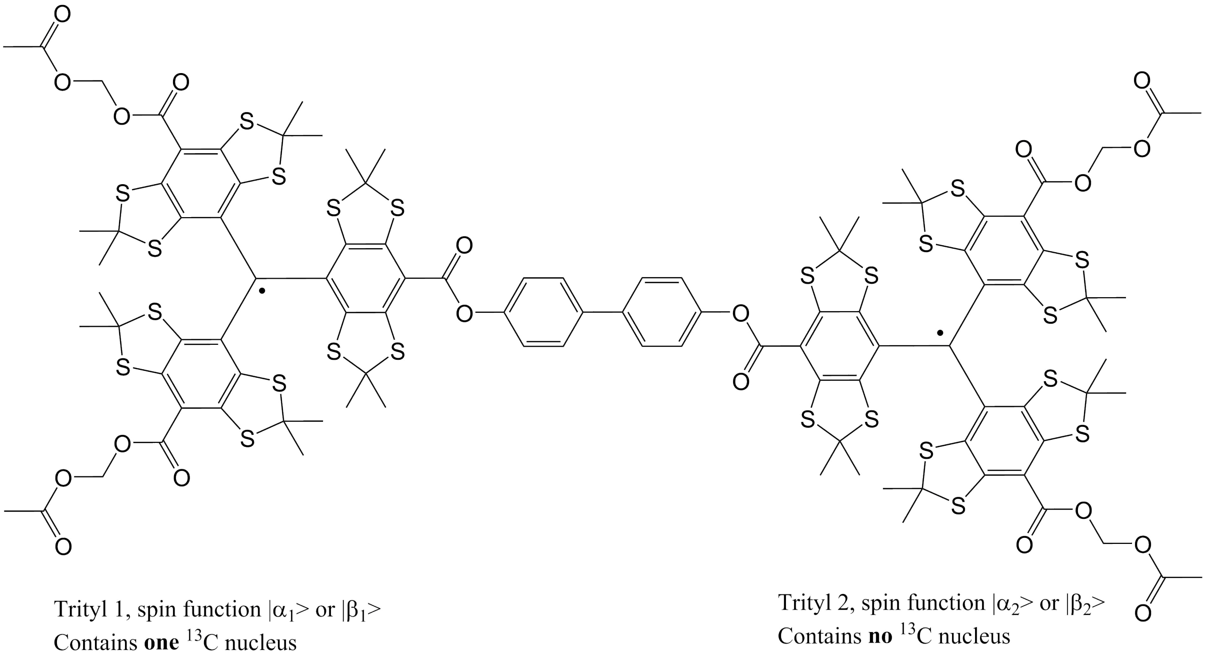

Figure S37: Structure of **4b••** containing one ^{13}C nucleus in one of the two trityl groups.

Thus, the spin system consists of two electron spins and one nuclear spin. As basis set, the product functions of the individual spins are considered

$$|1> = |\alpha_1\alpha_2\alpha_{{}^{13}_{1}C}> \quad \text{(S-1)}$$
$$|2> = |\alpha_1\beta_2\alpha_{{}^{13}_{1}C}> \quad \text{(S-2)}$$
$$|3> = |\beta_1\alpha_2\alpha_{{}^{13}_{1}C}> \quad \text{(S-3)}$$
$$|4> = |\beta_1\beta_2\alpha_{{}^{13}_{1}C}> \quad \text{(S-4)}$$
$$|5> = |\alpha_1\alpha_2\beta_{{}^{13}_{1}C}> \quad \text{(S-5)}$$
$$|6> = |\alpha_1\beta_2\beta_{{}^{13}_{1}C}> \quad \text{(S-6)}$$
$$|7> = |\beta_1\alpha_2\beta_{{}^{13}_{1}C}> \quad \text{(S-7)}$$
$$|8> = |\beta_1\beta_2\beta_{{}^{13}_{1}C}> \quad \text{(S-8)}$$

By definition, the nuclear spin is located on trityl 1 as depicted in Figure S37. The spin Hamiltonian operator in frequency units for this system can be derived from eq. 1 in the main text and reads as

$$H = \frac{\mu_B B_0}{h}(g_1 S_{1,z} + g_2 S_{2,z}) + a\left(S_{1,z}I_z + \frac{1}{2}\left(S_{1,+}I_- + S_{1,-}I_+\right)\right)$$
$$+J\left(S_{1,z}S_{2,z} + \frac{1}{2}\left(S_{1,+}S_{2,-} + S_{1,-}S_{2,+}\right)\right) \quad \text{(S-9)}$$

The terms in the operator represent the Zeeman effect for both spins, the

isotropic hyperfine interaction, and the isotropic exchange interaction. For the latter two interactions, secular and pseudosecular interactions are taken into account in eq. S-9. The spin Hamiltonian operator can be simplified further. First off all, the isotropic g-values of both spin centers are identical. Second, the pseudosecular term of the isotropic hyperfine interaction can be neglected, as it links functions |3> and |5> as well as |4> and |6>. The eigenvalues of these levels differ by the electron Zeeman energy which is by far the largest contribution to the spin Hamiltonian in molecules **4a••** and **4b••**. Thus, the truncated Hamiltonian operator reads as

$$H = \frac{\mu_B B_0 g}{h}(S_{1,z} + S_{2,z}) + aS_{1,z}I_z + J\left(S_{1,z}S_{2,z} + \frac{1}{2}\left(S_{1,+}S_{2,-} + S_{1,-}S_{2,+}\right)\right) \quad \text{(S-10)}$$

The corresponding Hamiltonian matrix $\overline{\overline{H}}$ for the functions |1> to |4> is given by

$$\overline{\overline{H}} = \begin{pmatrix} \frac{\mu_B B_0 g}{h} + \frac{J+a}{4} & & & \\ & -\frac{J-a}{4} & \frac{J}{2} & \\ & \frac{J}{2} & -\frac{J+a}{4} & \\ & & & -\frac{\mu_B B_0 g}{h} + \frac{J-a}{4} \end{pmatrix} \quad \text{(S-11)}$$

The matrix for the other functions is similar, only the signs of hyperfine terms have to be changed as these functions correspond to the other alignment of the nuclear spin. There is also no mixing between the first four functions (nuclear spin up) and the second four functions (nuclear spin down). Hence it is sufficient to discuss the functions and eigenvalues corresponding to one of the nuclear spin states. Importantly, functions |1> and |4> do not mix with any other function and are thus good wavefunctions regardless of the magnitude of J and the hyperfine coupling constant a. The eigenvalues of |1> and |4> can be read off directly from the matrix in eq. S-11. |2> and |3> are mixed to arrive at new wavefunctions |2'> and |3'>

$$|2'>=c_1|2>+c_2|3> \quad \text{(S-12)}$$

$$|3'>=c_2|2>-c_1|3> \quad \text{(S-13)}$$

with the orthonormality conditions

$$c_1^2+c_2^2=1 \quad \text{(S-14)}$$

and

$$<2'|3'>=<3'|2'>=0. \quad \text{(S-15)}$$

The submatrix of $\overline{\overline{H}}$ involving |2> and |3> (eq. S-11) can be used to calculate the eigenvalues and mixing coefficients of the new wavefunctions |2'> and |3'>. For the eigenvalues, the determinant is considered:

$$\begin{vmatrix} -\frac{J-a}{4}-E & \frac{J}{2} \\ \frac{J}{2} & -\frac{J+a}{4}-E \end{vmatrix}=0 \quad \text{(S-16)}$$

or

$$E^2+\frac{J}{2}E-\frac{3}{16}J^2-\frac{a^2}{16}=0 \quad \text{(S-17)}$$

yielding

$$E_{2',3'}=-\frac{J}{4}\pm\frac{1}{2}\sqrt{J^2+\frac{a^2}{4}}=-\frac{J}{4}\pm\frac{1}{2}\sqrt{J^2+\Delta\omega^2} \quad \text{(S-18)}$$

where *a/2* has been replaced by $\Delta\omega$ in the last step and the upper sign before the square root refers to function |2'>. To obtain the eigenfunctions |2'> and |3'> to these eigenvalues, the secular equations have to be solved:

$$\begin{aligned} \left(\frac{\Delta\omega}{2}-\frac{J}{4}-E\right)\cdot c_1 &+ \frac{J}{2}\cdot c_2 &= 0 \\ \frac{J}{2}\cdot c_1 &+ \left(-\frac{\Delta\omega}{2}-\frac{J}{4}-E\right)\cdot c_2 &= 0 \end{aligned} \quad \text{(S-19)}$$

yielding together with the normalization condition (eq. S-14)

$$c_1=\sqrt{\frac{J^2}{J^2+\left(\left(\sqrt{J^2+\Delta\omega^2}\mp\Delta\omega\right)\right)^2}} \quad \text{(S-20)}$$

$$c_2 = \sqrt{\frac{((\sqrt{J^2+\Delta\omega^2}\mp\Delta\omega))^2}{J^2+((\sqrt{J^2+\Delta\omega^2}\mp\Delta\omega))^2}} \quad \text{(S-21)}$$

Eqs. S-18, S-20, and S-21 show that the coefficients and energies of state |2′> and |3′> (upper and lower sign before $\Delta\omega$, respectively) depend in a complex manner on $\Delta\omega$ and J. In the extreme case of $|J|$ << $|\Delta\omega|$ the functions |2′> and |3′> are identical to |2> and |3>, respectively (i.e. $c_1 = 1$ and $c_2 = 0$ in the case of |2′> and vice versa for |3′>). The eigenvalues of the resulting functions are identical to the diagonal elements in the matrix $\bar{\bar{H}}$ given in eq. S-11. In this weak coupling case, the molecule behaves like a real biradical, meaning that it is possible to selectively address either one of the electron spins spectroscopically. As a consequence, transitions in which a single electron spin is flipped are allowed in this regime, i.e. transitions |4> → |3>, |4> → |2>, |3> → |1>, and |2> → 1> with the transition frequencies

$$v_{|4>\to|3>} = \frac{\mu_B B_0 g}{h} - \frac{a}{2} - \frac{J}{2} \approx \frac{\mu_B B_0 g}{h} - \frac{a}{2} \quad \text{(S-22)}$$

$$v_{|4>\to|2>} = \frac{\mu_B B_0 g}{h} - \frac{J}{2} \approx \frac{\mu_B B_0 g}{h} \quad \text{(S-23)}$$

$$v_{|3>\to|1>} = \frac{\mu_B B_0 g}{h} + \frac{a}{2} + \frac{J}{2} \approx \frac{\mu_B B_0 g}{h} + \frac{a}{2} \quad \text{(S-24)}$$

$$v_{|2>\to|1>} = \frac{\mu_B B_0 g}{h} + \frac{J}{2} \approx \frac{\mu_B B_0 g}{h} \quad \text{(S-25)}$$

where the approximate result given at the end of each equation is exact for the case J = 0. The case J = 0 is of interest to obtain the values for the isotropic hyperfine coupling constants a and is realized for radicals **1a$^\bullet$**, **2a/b$^{\bullet\bullet}$** and **3a$^{\bullet\bullet\bullet}$**. Taking compound **2a$^{\bullet\bullet}$** as an example, these transition frequencies correspond to the hyperfine doublet lines (eqs. S-22 and S-24) caused by the trityl group with a ^{13}C atom and to the central line caused by a trityl group without active nucleus (eqs S-23 and S-25), respectively. The other extreme corresponds to the strong coupling case where $|J|$ >> $|\Delta\omega|$. In this situation, $c_1 = \pm c_2 = \frac{1}{\sqrt{2}}$ leading to the triplet state

function |2′> (upper sign) and the singlet state function |3′> (lower sign). Their eigenvalues amount to

$$E_{2\prime} = \frac{J}{4} \tag{S-26}$$

$$E_{3\prime} = -\frac{3J}{4} \tag{S-27}$$

Importantly, transitions involving the singlet state function are forbidden in this regime. This leaves two allowed transitions between the triplet states with frequencies

$$\upsilon_{|4> \rightarrow |2'_{\mathrm{triplet}}>} = \frac{\mu_B B_0 g}{h} - \frac{a}{4} \tag{S-28}$$

$$\upsilon_{|2'_{\mathrm{triplet}}> \rightarrow |1>} = \frac{\mu_B B_0 g}{h} + \frac{a}{4} \tag{S-29}$$

Thus, in the strong coupling a pair of transitions is still observed, but with a splitting which is reduced by 50%. An example for this are the hyperfine coupling cosntants to the *a*-protons in **4a**$^{\bullet\bullet}$, which are lowered by 50% as compared to **2a**$^{\bullet\bullet}$ and **3a**$^{\bullet\bullet\bullet}$. The most complicated case is encountered when *J* is on the order of *a*. The transition frequencies in are then given by eqs. S-30 – S-33:

$$\upsilon_{|4> \rightarrow |3\prime>} = \frac{\mu_B B_0 g}{h} + \frac{a}{4} - \frac{J}{2} - \frac{1}{2}\sqrt{J^2 + \frac{a^2}{4}} \tag{S-30}$$

$$\upsilon_{|4> \rightarrow |2>} = \frac{\mu_B B_0 g}{h} + \frac{a}{4} - \frac{J}{2} + \frac{1}{2}\sqrt{J^2 + \frac{a^2}{4}} \tag{S-31}$$

$$\upsilon_{|3> \rightarrow |1>} = \frac{\mu_B B_0 g}{h} + \frac{a}{4} + \frac{J}{2} + \frac{1}{2}\sqrt{J^2 + \frac{a^2}{4}} \tag{S-32}$$

$$\upsilon_{|2> \rightarrow |1>} = \frac{\mu_B B_0 g}{h} + \frac{a}{4} + \frac{J}{2} - \frac{1}{2}\sqrt{J^2 + \frac{a^2}{4}} \tag{S-33}$$

These equations have to be used for the *ipso*- and *ortho*-satellite lines. Since cw EPR spectroscopy is usually conducted at constant MW frequency

υ_{MW} while the field values are swept it is sensible to rewrite the above equations to obtain the resonant field of all transitions:

$$B_{|4> \rightarrow |3'>} = -\frac{h}{\mu_B g}\left(\frac{a}{4} - \frac{J}{2} - \frac{1}{2}\sqrt{J^2 + \frac{a^2}{4}} - \upsilon_{\mathrm{MW}}\right) \quad \text{(S-34)}$$

$$B_{|4> \rightarrow |2>} = -\frac{h}{\mu_B g}\left(\frac{a}{4} - \frac{J}{2} + \frac{1}{2}\sqrt{J^2 + \frac{a^2}{4}} - \upsilon_{\mathrm{MW}}\right) \quad \text{(S-35)}$$

$$B_{|3> \rightarrow |1>} = -\frac{h}{\mu_B g}\left(\frac{a}{4} + \frac{J}{2} + \frac{1}{2}\sqrt{J^2 + \frac{a^2}{4}} - \upsilon_{\mathrm{MW}}\right) \quad \text{(S-36)}$$

$$B_{|2> \rightarrow |1>} = -\frac{h}{\mu_B g}\left(\frac{a}{4} + \frac{J}{2} - \frac{1}{2}\sqrt{J^2 + \frac{a^2}{4}} - \upsilon_{\mathrm{MW}}\right) \quad \text{(S-37)}$$

If the hyperfine coupling constants and the microwave frequency are known it is then possible to plot the resonant fields as a function of the isotropic exchange coupling constant J (Figure 2 in the main text). Comparison of the observed line positions with the calculated line positions then allows deriving the correct value of $|J|$, as shown in Figures 2 and 5 in the main text. As discussed, $|J|$ decreases from ~75 MHz at room temperature down to ~50 MHz at 213 K. If a melting point about 175 K is estimated for the solvent system one can extrapolate an average $|J|$ value of around 40 and 37.5 MHz for frozen solutions of **4a**$^{\bullet\bullet}$ and **4b**$^{\bullet\bullet}$, respectively. Note that it is not possible to extract the sign of J from the cw EPR spectra. However, the positive sign appears to be the right choice. With the definition of the spin Hamiltonian operator given in eq. 1 in the main text this means that the spin system is antiferromagnetically coupled, which is also in agreement with the attachment of the spin centers in para positions of the diamagnetic bridging unit [4,5].

3.3 Dipolar, spectral broadening caused by one or two spins

As mentioned in the main text, the EPR spectrum of bistrityl **2a**$^{\bullet\bullet}$ was found to be slightly broader than the EPR spectrum of the analogous monotrityl compound **1a**$^{\bullet}$. Furthermore, the spectrum of tristrityl **3a**$^{\bullet\bullet\bullet}$

was found to be further broadened. The interaction with two electron spins instead of just one was mentioned as the origin of this additional broadening, which could be seen as manifestation of multi-spin effects [6—8] in cw EPR spectroscopy. Moreover, the situation was compared to the scenario of an electron spin coupling to either one or two nuclear spins. This interpretation is a different angle on multi-spin effects and is discussed here for better understanding. To that end, Figure S38 is considered, in which the orientations of the interspin vectors with respect to the external magnetic field are depicted for three different molecular orientations. Figure S38 also gives the expected coupling of spin **A** with the two other spins in multiples of the dipolar coupling constant *D*. These values can now be used to draw energy level schemes similar to those drawn in textbooks when discussing isotropic hyperfine interactions. This is shown in Figure S39 for a two-spin system **AB** and a three-spin system **ABC**.

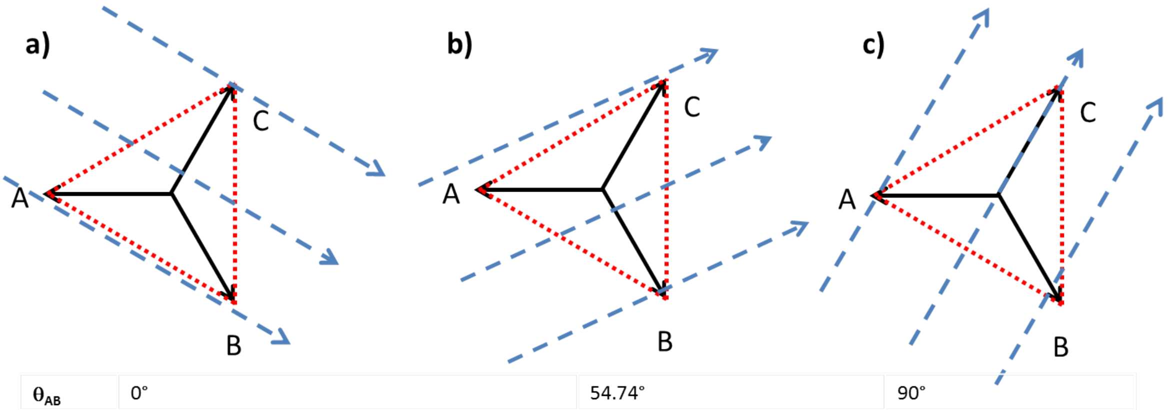

θ_{AB}	0°	54.74°	90°
ν_{AB}/D	-2	0	1
θ_{AC}	60°	5.26°	30°
ν_{AC}/D	0.25	-1.98	-1.25

Figure S38: Molecular triangle with spin centers **A**, **B**, and **C** in a magnetic field B_0 (blue dashed arrows) with different spatial alignment. The vectors joining the three spin centers are given by red dotted lines. **a)** B_0 parallel to vector **AB**. **b)** B_0 forming an angle of 54.74° (the magic angle) with vector **AB**. **c)** B_0 perpendicular to **AB**.

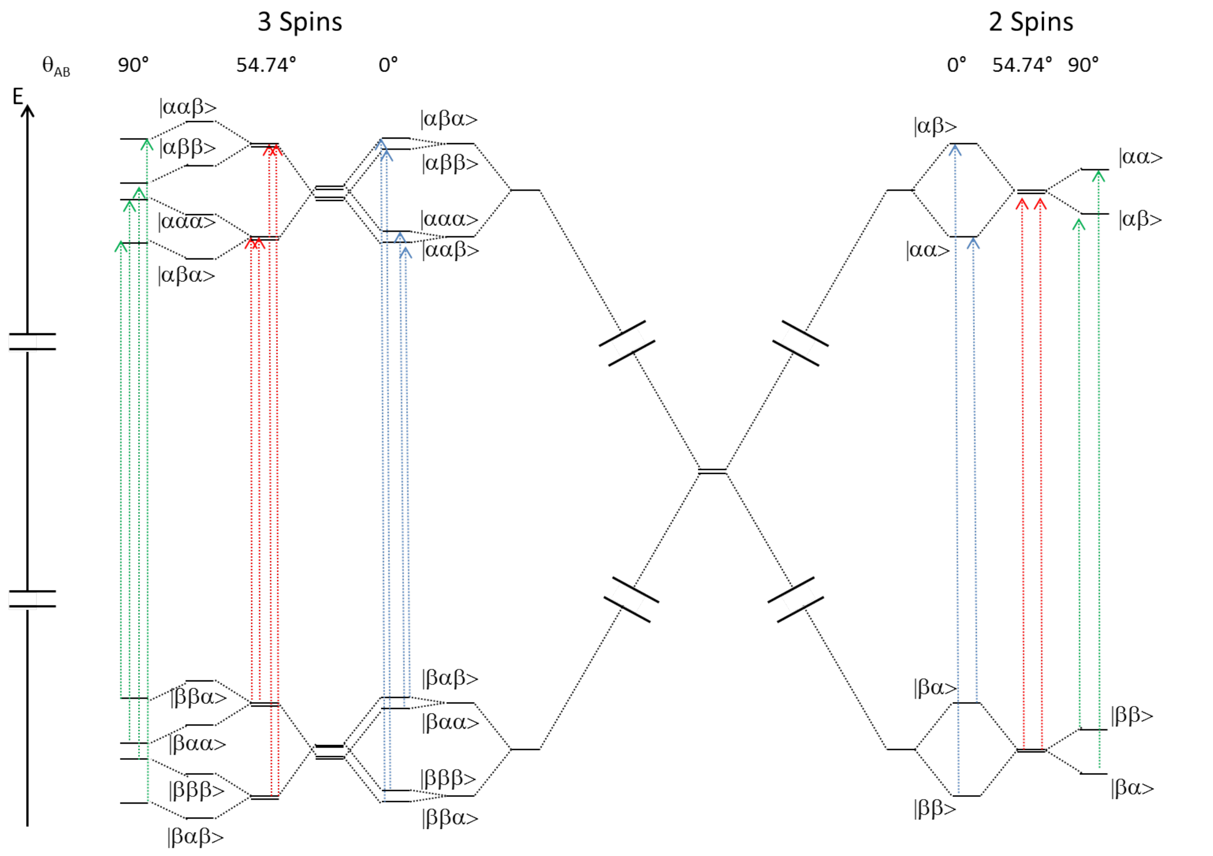

Figure S39: Energy level diagram for one observed spin **A** coupling to either one spin (right-hand side) or two spins (left hand side). The energy levels are given for the three different orientations given in Figure S38. The allowed transitions between the spin states (horizontal, black lines) for the three orientations are given by dotted arrows in different colors. Degenerate states are indicated as double lines. The spin functions for the magic angle are omitted to prevent crowding of the Figure.

As can be seen by comparing the three-spin to the two-spin example depicted in Figure S39, the amount of transitions is larger in the three spin example, as there are also more different spin states. Each spin state is shifted from the position obtained by the Zeeman-splitting of spin **A** by the electron-electron dipole coupling. For the three-spin system, these shifts are just the sum of the individual couplings to spin **B** and **C**. This leads to a broadening of the cw EPR spectrum of a three-spin system as compared to a two-spin system. In addition, also the multi-spin effects observed in pulsed measurements can be related to the scheme shown in Figure S39.

This is illustrated in Figure S40, which shows spectra obtained by numerical simulation of the couplings obtained for either a two-spin

system or a three-spin system in a rigid, triangular system as shown in Figure 39. Note that the spectrum of the three-spin system has no obvious resemblance to a Pake pattern. Another important point to note is the high intensity of the spectrum at high absolute values above and below the frequencies at the singularities of the two spin system. This is a consequence of the occurrence of sum- and difference frequencies and in agreement with the experimental observations made on **2a••** and **3a•••**.

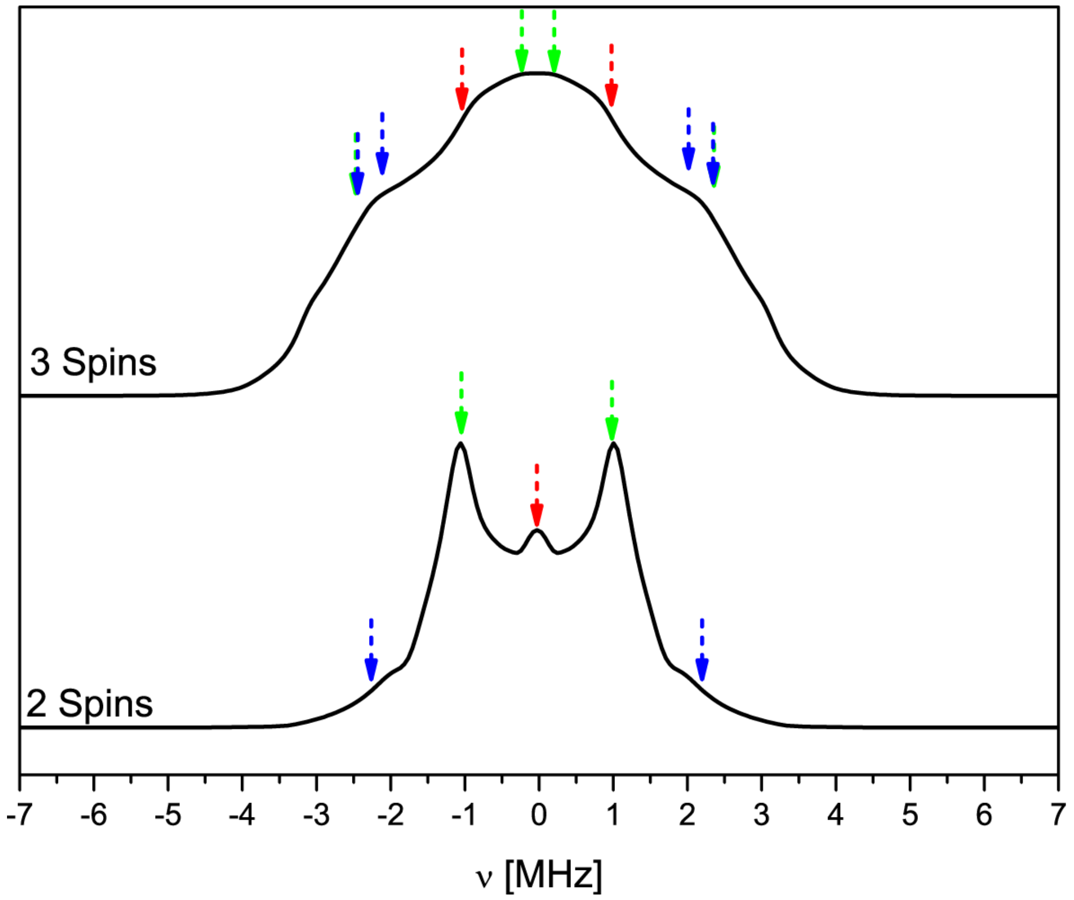

Figure S40: Numerically simulated EPR spectra of the dipolar interaction for a two-spin or a three-spin system. The frequencies of the transitions discussed in Figure S39 are indicated by colored arrows, the color code is identical to the one used in Figure S39. Note that two of the green and blue transitions are degenerate for the three-spin system.

4. XYZ Coordinates of $2a^{\bullet\bullet}$, $3a^{\bullet\bullet\bullet}$ and $4a^{\bullet\bullet}$ obtained by DFT

$2a^{\bullet\bullet}$

258 atoms

C	-5.23392976062524	-1.41760520225763	1.12207487672178
C	-3.85847268002417	-1.63316046961419	1.25796892415919
C	-2.96016056568061	-1.31178980321741	0.21224230989570
C	-3.49701200975522	-0.74934180347421	-0.97003124571933
C	-4.86991913962475	-0.51979250841598	-1.12110437844940
C	-5.73456269680670	-0.86443612439222	-0.06812187601860
H	-5.94105724407515	-1.68556369490956	1.91941756572173
H	-3.47533437329036	-2.09346947349035	2.18057124215531
H	-2.81913818021371	-0.45749714463419	-1.78579239326890
H	-5.26901903660096	-0.08320573049941	-2.04357647718979
C	-1.50639324593470	-1.56593039165640	0.34407545282198
C	-0.72767473042456	-1.93713093734851	-0.77922069967721
C	-0.84946690288550	-1.45463518732154	1.59355376788567
C	0.64048401327909	-2.18799705806210	-0.66470385695068
H	-1.21660035274002	-2.06199915197684	-1.75690013175428
C	0.51876489714434	-1.70343763569057	1.72033580844680
H	-1.42115169002248	-1.13800170471212	2.47857406284846
C	1.29493564273582	-2.07797199698244	0.59127548150152
H	1.22459915229331	-2.48876581994614	-1.54627495813093
H	1.01246489230944	-1.60105918572946	2.69773546902093
O	-7.10438339167892	-0.63409778380056	-0.10407665183555
O	13.50391251511754	-5.99815414451250	-10.99624365411417
C	-7.86020374942344	-0.90193447839191	-1.22192653324262
C	14.73146009365745	-5.61359807007342	-11.46510843818355
O	15.46304662744816	-4.81623021813921	-10.89516551241559
O	-7.38622499913243	-1.25957178125156	-2.28904139190000
C	15.06258953262947	-6.29775296922658	-12.73018202101777

C	14.29674015784989	-7.36863345812008	-13.27797175208084
C	16.26920945478056	-5.90201350846487	-13.38475738030359
C	14.78099771352354	-8.05568064425008	-14.41542352079420
C	16.71936408218838	-6.57832033677102	-14.53929340080821
C	15.96035295256486	-7.64790560685851	-15.09507769415723
C	-9.29551790449040	-0.68173479260065	-0.97149127544814
C	-9.87168298274441	-0.60000175869543	0.32965352501106
C	-10.14362774680083	-0.57341350865016	-2.11063834965901
C	-11.26070956242380	-0.39081370616842	0.47132973999855
C	-11.53695443558342	-0.40809295983413	-1.95299813928231
C	-12.11576434315464	-0.30678513768001	-0.66007569459701
C	-13.55181574029470	-0.10299849706071	-0.48929240429930
C	16.31563934592929	-8.25331417083592	-16.37446383172790
C	15.28003058930519	-8.42640046433506	-17.38289075844522
C	14.29040795032702	-7.42887101444633	-17.60497406655946
C	15.23703705633332	-9.61146410901671	-18.16738739439077
C	13.30662038402329	-7.59942580141185	-18.60289059141938
C	14.21495853650622	-9.82125597285963	-19.12101629944977
C	13.24934212182805	-8.80292761666970	-19.36002191467027
C	17.70849585009481	-8.50521170978515	-16.69518433410788
C	18.27878375493387	-8.07172697721577	-17.93155665648813
C	18.57358854937322	-9.07852854061495	-15.71878310188517
C	19.68235952670987	-8.07534070417259	-18.10607999940702
C	19.97525123424860	-9.10758557472196	-15.89060454161266
C	20.54145868188052	-8.61271245445558	-17.10102738185284
C	-14.47707910214091	-0.81506564586525	-1.35931732629440
C	-14.27146863402181	-2.18858857617535	-1.66680630025960
C	-15.55119865392425	-0.13436435570428	-2.00207578043280
C	-15.02687441194325	-2.84862570245028	-2.66438542031219
C	-16.31387014140618	-0.77913432121431	-2.99993223425298

C	-16.07540826417762	-2.14788813781710	-3.32686754824687
C	-14.03500004510162	0.86983775175256	0.47846893471317
C	-15.14550711232520	0.56832425317070	1.31692641060113
C	-13.40910705843724	2.14299618448802	0.62637011511806
C	-15.63009440050018	1.50997170223929	2.25237358786348
C	-13.82459183959621	3.05635534760168	1.62186410407280
C	-14.95804726358740	2.75287314669462	2.43178327127793
S	20.28280144272690	-7.34989556384975	-19.60500457578285
S	14.24422034521337	-11.40155116785675	-19.92375941992596
S	12.18291367342136	-6.24850526010203	-18.82766983806411
S	20.88216002820460	-9.81557240755856	-14.54441635995850
S	18.22819381684311	-5.99283896456609	-15.25769377498476
S	13.94479369788941	-9.55045099369320	-14.85273064805949
S	-11.88647627471790	-0.27866143778686	2.12748736987617
S	-12.48582916353510	-0.30354799089273	-3.44813089111990
S	-17.52783911857039	0.22185339216513	-3.81383369806476
S	-17.02242397926432	1.00051369952976	3.21226351509703
S	-12.89450579907645	4.56222451909971	1.70054303636954
S	12.77995715911198	-7.99962184059262	-12.61503852168561
S	17.26660623564294	-4.54017860746930	-12.84602197389347
S	17.92176040649192	-9.92133764711568	-14.31285887003724
S	17.31708138653605	-7.43458977388739	-19.27043331515901
S	16.40978953510909	-10.91957476985253	-17.94701722458533
S	14.22758748620318	-5.90946128959636	-16.69593413047005
C	15.25162720476547	-12.22252845488122	-18.58537264689427
C	13.33335292286709	-5.00233462714853	-18.04313396442767
C	18.70246161480171	-6.40709125677799	-19.94408268052575
C	19.47146934898086	-9.72656569513334	-13.31789164444634
C	18.07518981054169	-4.30405812858664	-14.51390107715355
C	12.33407858792651	-9.00501266335472	-14.12840381265974

C	19.47738877583374	-8.36587911872667	-12.61888389002953
H	18.63394211754034	-8.30287066435931	-11.90041565098015
H	20.43079270945015	-8.22575255912089	-12.06738883104951
H	19.36255450821508	-7.54116324569970	-13.34958719534055
C	19.59402487462888	-10.89786225434140	-12.34230416155745
H	20.54064651579240	-10.81872497568436	-11.76957142807341
H	18.75926840341662	-10.87307805288063	-11.61165710240616
H	19.57844060727147	-11.86846099155532	-12.87392418386959
C	17.17945353784433	-3.43362251162920	-15.39934429025690
H	17.01634615498251	-2.44387400140463	-14.92492464636438
H	17.65189017131637	-3.28388672851962	-16.39216494652155
H	16.19588398652101	-3.91827401399947	-15.55850960580322
C	19.47024005234088	-3.71320518467220	-14.31227256382510
H	19.97468775183920	-3.57919341256124	-15.29068354454056
H	19.39595155524093	-2.71519026535037	-13.83359254265406
H	20.09411684950975	-4.36866877781015	-13.67520419029473
C	18.73969643461925	-5.06654216774381	-19.20519075337468
H	19.61083875610499	-4.46530722761778	-19.54116834946184
H	17.80906806206486	-4.49425467296751	-19.40447753774455
H	18.81443522180915	-5.22851883311952	-18.11121590747357
C	18.53116974278818	-6.25509225854126	-21.45507249797451
H	17.59251492393964	-5.70937952600977	-21.68380346416471
H	19.37158338306556	-5.66675963990322	-21.87687621162048
H	18.50247443085317	-7.24220642596148	-21.95417102741786
C	16.06443145885437	-13.36003501636174	-19.20189421456720
H	16.68643737901484	-13.84879549335029	-18.42429285855682
H	15.38702604486966	-14.13122808166243	-19.62230808808390
H	16.72697357009518	-12.98770286248495	-20.00671457858176
C	14.32884186430807	-12.68588659041948	-17.45664777434702
H	13.60058893038315	-13.43425699166531	-17.83383955639673

H	14.92520440555238	-13.14073008314480	-16.63885128085297
H	13.76895545705538	-11.82743488145263	-17.03607458053921
C	12.50207726550294	-3.87828662702697	-17.42704861443698
H	13.16388939469089	-3.13178320824552	-16.94225252381448
H	11.92681972717801	-3.35312496879257	-18.21691988680795
H	11.79295395232479	-4.26909499730408	-16.67178719299146
C	14.34345617933024	-4.50208727121143	-19.07807428203885
H	13.81825743467285	-4.01942111663094	-19.92824916748322
H	15.03261430531800	-3.76551289883581	-18.61409511145389
H	14.95275317114158	-5.34235228505601	-19.46572781458300
C	11.53434252363695	-10.23011651396073	-13.68500120737669
H	10.58549368048267	-9.91270613667377	-13.20683146229722
H	11.27110225581820	-10.85021790444817	-14.56598647291163
H	12.10850925171417	-10.84642395647811	-12.96719660731206
C	11.58175069046033	-8.12717670043646	-15.13015603484593
H	12.19412160799227	-7.25729896391259	-15.44077985529027
H	11.33822509770999	-8.71045007964168	-16.04206181609321
H	10.63794780952798	-7.75516707236376	-14.68050652664995
S	-8.97784251892774	-0.75750302547331	1.85531990790473
S	-9.55051727279625	-0.62806216104945	-3.77862423041576
S	-15.86006678186214	-1.04363903697712	1.37878553884107
S	-12.20943753821822	2.73618654652764	-0.52946277194941
S	-13.18168136620757	-3.19345963279527	-0.70522551800994
S	-15.95017947959792	1.55339144190858	-1.66007994238564
S	-14.58969595757600	-4.54187787461285	-2.94320860784192
C	-16.70998999084072	1.83343106883874	-3.32678675526157
C	-17.46253844641325	-0.43071851216738	2.08192357892026
C	-11.46557663813224	3.92835250766011	0.67522411689049
C	-11.04278608715876	0.25591573639061	-4.46904302188337
C	-10.47803530905144	-1.25072239126322	2.84343630093404

C	-12.95039704162989	-4.44836380249174	-2.04535151427329
C	-17.77582943262121	2.92290633448199	-3.22538719896590
H	-17.31507336114824	3.88517895729750	-2.92245823617043
H	-18.25673462780516	3.07868204972114	-4.21281963444787
H	-18.55593000796883	2.65201186568968	-2.48863562683695
C	-15.60277361614814	2.16693641013495	-4.33024853620321
H	-16.02775524592082	2.27643799453363	-5.34945991342210
H	-15.10072914990394	3.11442197827713	-4.04221009914980
H	-14.83663487649409	1.36620154084411	-4.34538353861312
C	-12.65788749761124	-5.81063526664118	-1.41507885962768
H	-12.56352131349471	-6.58326055831125	-2.20522833835979
H	-11.69448901479680	-5.77542380919758	-0.86684424139515
H	-13.46112612372301	-6.10960508255847	-0.71496534547555
C	-11.85679228478214	-3.98732115778922	-3.01050620811010
H	-10.88438699454302	-3.91396135058402	-2.48096688609663
H	-11.75706942182133	-4.70796705900590	-3.84856265992913
H	-12.09098701299985	-2.98822333177618	-3.42788492615236
C	-11.25673866910240	-0.18090863900479	-5.91780634006378
H	-11.38298087334245	-1.27849364698373	-5.98996173385919
H	-12.15799279031292	0.31128297196847	-6.33753260886608
H	-10.38982737754578	0.12000121858543	-6.54144531504077
C	-10.86315810299957	1.76792602134742	-4.31972228513877
H	-9.96651148656322	2.10615104755836	-4.87965134493510
H	-11.75433819763377	2.30061605602089	-4.71205230169498
H	-10.73833857447364	2.04376627340031	-3.25390383750692
C	-10.27074907925915	-0.84380194231906	4.30174062604247
H	-11.16948410654009	-1.09762356483532	4.90041290464600
H	-9.41209407161513	-1.39601167711693	4.73542936129674
H	-10.07873140755218	0.24268837782503	4.39082395230876
C	-10.74508659048890	-2.74701153336866	2.66971479183288

H	-9.88775599678585	-3.33928051740790	3.05204852304267
H	-11.66122112959825	-3.03723542054956	3.22388123055948
H	-10.89945174927326	-2.99550890923201	1.60109593792143
C	-10.45271866466515	3.20532500599019	1.56428142008479
H	-10.91235702032068	2.33151220121851	2.06724453653948
H	-9.60667126362083	2.83157966129360	0.95135907440507
H	-10.05787289209604	3.89804646081865	2.33677025408187
C	-10.85448245696259	5.10161287035715	-0.09175175880253
H	-10.43325674403952	5.84308712881803	0.61765397728864
H	-10.02270991798169	4.74523227186888	-0.73384941645849
H	-11.60964887169967	5.60321316348160	-0.72681410872014
C	-18.10203504407788	-1.53483018123431	2.92362963896794
H	-19.04463421562128	-1.16653346866318	3.37732055811335
H	-18.35766050821518	-2.40507904514168	2.28538994748657
H	-17.42387216672398	-1.86799207723395	3.73219689620815
C	-18.36627220339937	0.05865177092256	0.94922145014098
H	-17.85739944461227	0.83095278331401	0.33975021523371
H	-18.62423311684023	-0.78298473602054	0.27363011985846
H	-19.30092917573704	0.48754683482745	1.36615896760002
C	-15.47938552288619	3.65925170239733	3.47663342003944
O	-16.31844797539674	3.34518591878113	4.31310125317999
O	-14.94152557626929	4.90293526077723	3.40982981260591
C	-16.99603482890101	-2.78091551886166	-4.29554127009290
O	-17.97300849299419	-2.22825827055178	-4.78894142258283
O	-16.66940208639749	-4.06769565346934	-4.57274359536794
C	12.15987621539966	-8.93406504019035	-20.35027590775119
O	11.16585873600408	-8.21816440694872	-20.38840696580881
O	12.38328297701595	-9.93141873259942	-21.24264915854629
C	21.98441361789840	-8.68845375777286	-17.41533304283035
O	22.48353941965279	-8.34528122299326	-18.48137259206873

O	22.71807399382212	-9.21532659795428	-16.40390293260561
C	24.12293340212618	-9.35160140362053	-16.64810515538992
H	24.54341035480372	-9.81199713346643	-15.73698944105064
H	24.30585231050344	-9.99398692145901	-17.53253069390187
H	24.58616937932165	-8.36169511318351	-16.83177394357785
C	11.34336785263489	-10.16076573260786	-22.20163507673976
H	11.67396388563064	-11.02478655685508	-22.80412472156873
H	10.38328253764779	-10.38366018959122	-21.69516160965805
H	11.20239169821099	-9.26855035074337	-22.84376770494889
C	-15.36488791340734	5.82720253834628	4.41924838898743
H	-14.82090817090768	6.76573153131152	4.21597049502570
H	-16.45962575590342	5.99038230923091	4.36439337704500
H	-15.11680939193559	5.44503008131414	5.42963574997992
C	-17.54001550609665	-4.75824283047319	-5.47692573461384
H	-17.11566835164555	-5.77114277223345	-5.58865820621800
H	-18.56872395672588	-4.80911740878848	-5.06760519503040
H	-17.57782510351431	-4.24077348620206	-6.45606759417491
C	2.68796916264417	-2.34263983204095	0.70842147893959
C	3.89400949222796	-2.58178896677417	0.79278552010760
C	5.28736394110010	-2.86790995205221	0.87492559283669
C	5.95624412768183	-2.88520039211193	2.12864779031944
C	6.02503050678203	-3.14770204346645	-0.30026155335443
C	7.32395993796276	-3.18218850553695	2.19168110670389
H	5.38626249610878	-2.66627599299010	3.04263490280129
C	7.40743584759336	-3.44572701854518	-0.23843897684537
H	5.51639698147008	-3.13634271565005	-1.27409827454593
C	8.05206649146294	-3.46300878637179	1.02792684046350
H	7.83299765386854	-3.19535574305437	3.16753856774138
H	9.12428587241398	-3.69984741631405	1.07959327286104
C	8.13347079326429	-3.72176043952798	-1.43298560289121

C	8.75045357002301	-3.95757811922388	-2.47352856365640
C	9.45047052108853	-4.22628194035239	-3.68283560118331
C	10.79639994707317	-4.68034948980684	-3.66752564144392
C	8.81791266582116	-4.04763315927669	-4.94238826051724
C	11.47245122685074	-4.94528589473617	-4.86036616645659
H	11.29724806077247	-4.83406932001120	-2.70078670115493
C	9.50417563040335	-4.31456560563779	-6.12849970398497
H	7.78139322832526	-3.68287215209990	-4.97131503615773
C	10.84524976897539	-4.77080499017579	-6.11843161040685
H	12.50308897917592	-5.32747966059648	-4.81947738774178
H	9.00029144495881	-4.13828274249489	-7.09061463506526
C	11.56473887788149	-5.05955227181268	-7.38063026579627
C	12.95517001941277	-4.83313118222555	-7.50269114114829
C	10.88153378976332	-5.57592959757122	-8.50841108835368
C	13.65383988750046	-5.10467953991967	-8.68725419384585
H	13.50828917581643	-4.40340745293438	-6.65427271971220
C	11.55819306526937	-5.85487175676067	-9.69808296477126
H	9.80532753843450	-5.79406713239319	-8.44103598767094
C	12.94333030221127	-5.62272120733656	-9.78557901201970
H	14.72600711075087	-4.89917039341822	-8.77041103859557
H	11.03896310695459	-6.27080281813976	-10.57299995193321

3a$^{\bullet\bullet\bullet}$

381 atoms

C	-5.32902266820964	-0.23827667334150	0.34147773321117
C	-3.93301872209046	-0.18790707129866	0.36343445892834
C	-3.19392094751477	0.11297662549502	-0.80624997844162
C	-3.91761034739594	0.36540602167508	-1.99421938528283
C	-5.31806546128750	0.32774459141165	-2.03686866036710

C	-6.02093426652229	0.01981655610867	-0.85691762156831
H	-5.90914154336351	-0.49141155520402	1.24027199501449
H	-3.40213492372711	-0.41843670868522	1.29910017346679
H	-3.37263820240310	0.63435974792351	-2.91140570140268
H	-5.86243684606003	0.55563383657113	-2.95874532901466
C	-1.71349537674648	0.16498589814960	-0.78781088146233
C	-0.95429120399317	-0.27461889705345	-1.89957317021359
C	-1.01064940398548	0.65895011714618	0.33792402398821
C	0.44094312599865	-0.22385762570434	-1.89196890458780
H	-1.47268448474944	-0.68890727972112	-2.77710967331615
C	0.38466782145966	0.72114138603453	0.35385667738926
H	-1.57511291992039	1.03196835367546	1.20556008627036
C	1.14175843586108	0.28036271677541	-0.76392570199027
H	1.01293306787311	-0.57912000859959	-2.76140373575261
H	0.91131899599308	1.12176897178109	1.23225029039630
O	-7.39787101713746	-0.09188440274799	-0.74660977586718
O	13.50072729855161	-8.01180751285911	-9.66097036517216
C	-8.31393596191783	0.25294601066087	-1.70898061997125
C	13.19228421136385	-8.39058148861771	-10.94185171328776
O	12.23626435405297	-7.95532035275910	-11.56829632005241
O	-8.04430617504682	0.85159095747739	-2.73954991957284
C	14.17274626096475	-9.35411307770860	-11.47595131168319
C	15.35442226373042	-9.75533465654711	-10.78617737981595
C	13.93752752192542	-9.84844886912960	-12.79628342213319
C	16.29349082828753	-10.58270602398982	-11.44413039155140
C	14.87900786103217	-10.68853009112343	-13.43033328510062
C	16.05461190068493	-11.10565687760876	-12.74356220165923
C	-9.67177276392091	-0.15625457493870	-1.30483031430537
C	-9.92775875136439	-1.16592029895699	-0.33247488407693
C	-10.77105238826625	0.48714984319535	-1.93881735359713

C	-11.25636615448266	-1.48779526357082	0.01546710930373
C	-12.09695980288234	0.13404652837221	-1.60286448862300
C	-12.35971013877699	-0.84553990885947	-0.60825090590027
C	-13.72556327576515	-1.16974603969418	-0.20667507331091
C	16.94307186476984	-12.12121609979181	-13.29940763873353
C	17.34420226607890	-13.24182659267943	-12.46274153382508
C	16.42932224793569	-13.86277059481787	-11.56692158720402
C	18.67389794562687	-13.74249380109191	-12.52911525208314
C	16.82468629960966	-14.97142952971403	-10.78751090478582
C	19.09784260738159	-14.80598766915606	-11.70021074913012
C	18.16620096265553	-15.44405589084855	-10.83416370718554
C	17.26096707910950	-12.10669074539571	-14.71565934583255
C	17.11779614862363	-13.27524042250253	-15.52508705579478
C	17.59662844881414	-10.87990139425451	-15.35717794959072
C	17.16883720079910	-13.17318019529791	-16.93501153395374
C	17.66403819176821	-10.76799336256816	-16.76377182218181
C	17.46256279940591	-11.92754174221454	-17.56693057104597
C	-14.76282792045432	-1.25671514017689	-1.22436818438096
C	-14.51347007120988	-1.92186256910826	-2.45629767797688
C	-16.01408371644289	-0.59758986384220	-1.05518197994462
C	-15.42265964257747	-1.84982759582712	-3.53732925073403
C	-16.93110474737243	-0.51508124184512	-2.12499763411846
C	-16.65623512349664	-1.15811848297452	-3.36932274396145
C	-14.04727893896594	-1.30499729612682	1.20583609942039
C	-14.89926737897663	-2.35266559129972	1.65822571551353
C	-13.51244735637456	-0.40932863155916	2.17932803976994
C	-15.22432373038732	-2.49065716173807	3.02664468667484
C	-13.74984049511668	-0.59634792196906	3.56016598937083
C	-14.63001318775570	-1.63060089157587	3.99410751726446
S	16.80814243498846	-14.65450630421678	-17.83528922875228

S	20.81555809827443	-15.22512997072071	-11.82809956645008
S	15.56673134176811	-15.70251768264961	-9.77733091548023
S	18.04108452365036	-9.14751569049007	-17.37009094388414
S	14.48483234062643	-11.21693154319882	-15.07354918418325
S	17.85334664912706	-10.80499074654004	-10.64411314231240
S	-11.47326747708777	-2.75195397740904	1.24151694682270
S	-13.37782927923118	1.01364847486156	-2.45894078163442
S	-18.38857045952442	0.44501901490170	-1.82384274334957
S	-16.30752390440906	-3.82542198661982	3.43470166528172
S	-12.95027770911549	0.56858023405648	4.62930018272176
S	15.80595604767462	-9.27304499598402	-9.14306739116294
S	12.47060477080377	-9.48967184655110	-13.72124325393635
S	18.09820890075150	-9.47423377716436	-14.41728626335575
S	16.77873751120485	-14.87753437841689	-14.85939591715224
S	19.89145586575843	-13.02548396200054	-13.59678481794891
S	14.74556658721174	-13.34100627978282	-11.38279374129725
C	21.31246156772050	-13.57204314735006	-12.53494876269891
C	14.17115665828877	-15.00597413144894	-10.80538724580569
C	15.94047086526589	-15.47776112569526	-16.39711895830832
C	17.67393926911317	-8.26864885325535	-15.75741619005348
C	12.66452738307812	-10.96011617777815	-14.85789901440919
C	17.15832492682377	-10.55216013277577	-8.94863673042795
C	16.18819030889658	-7.91287714943588	-15.68872171312190
H	15.95852174598796	-7.41872746990015	-14.72174808632690
H	15.92318094582978	-7.22286315808079	-16.51681614900381
H	15.55721265517597	-8.82062270129555	-15.76598061840222
C	18.58761982855290	-7.04746088132554	-15.64623216305273
H	18.37181206698756	-6.33625795356776	-16.46950030272763
H	18.39924348561905	-6.51656731591334	-14.69013218257024
H	19.65468774239412	-7.33799011741417	-15.69266673431789

C	12.04830734513309	-12.20008491516174	-14.20338667230263
H	10.97127897504502	-12.03045866200245	-13.99735358272303
H	12.15433591046278	-13.07724170728300	-14.87567559899954
H	12.56135079407685	-12.43643679383568	-13.25015013202347
C	12.03747086275260	-10.62677038218685	-16.21143854211526
H	12.15256973465045	-11.48096422140357	-16.91055231521014
H	10.95134005236569	-10.43514008825247	-16.09149907120191
H	12.50874011017019	-9.73149549275391	-16.66022052903161
C	14.47210204699959	-15.04550433211232	-16.37426155939160
H	13.97221791800740	-15.33172318254639	-17.32354794274811
H	13.94305630457667	-15.53196930984949	-15.52841441828018
H	14.39201211460051	-13.94779382520193	-16.24354244110004
C	16.11630763308340	-16.99156071686783	-16.51164382088094
H	15.61671236568610	-17.50365312127696	-15.66359417415220
H	15.64702824298506	-17.36047868955635	-17.44680450882371
H	17.18735434087184	-17.26930315852343	-16.51743029806518
C	22.55226267309396	-13.75790224044598	-13.40884501413193
H	22.85568779839243	-12.78777879175615	-13.85238305807901
H	23.40124141045138	-14.12495903754834	-12.79639488991616
H	22.36017077016418	-14.48020530778143	-14.22476888067513
C	21.52305824459988	-12.56714480254668	-11.40082366537087
H	22.33866678528785	-12.90832591357794	-10.72922375774784
H	21.78946768644568	-11.57363953721567	-11.81663118998376
H	20.59711959110255	-12.45229664983411	-10.80328490430960
C	12.93750167577019	-14.83345642019450	-9.92065897712918
H	12.10253280372571	-14.39728344754012	-10.50662251031708
H	12.59964880656594	-15.81888828572475	-9.54033306991016
H	13.15062058453123	-14.17278402116859	-9.05908086277758
C	13.91754576236491	-15.90663492682401	-12.01649920395524
H	13.64262445593562	-16.92910941728375	-11.68481896882575

H	13.09152731622498	-15.49374625282680	-12.63314102950410
H	14.82127676994253	-15.96920668304820	-12.65438453526588
C	18.24381243888660	-9.98245946574332	-8.03475275518130
H	17.82656801721336	-9.77947453667719	-7.02771997218991
H	19.06335693139754	-10.71991611893372	-7.91292768858253
H	18.66179183752240	-9.04368232707622	-8.44482860339541
C	16.56079628781519	-11.86014042807817	-8.42583174230067
H	15.77990227941495	-12.24630392533435	-9.11149665980228
H	17.35127239602224	-12.63452737277277	-8.34180801264378
H	16.10701956899874	-11.69898806121128	-7.42609950569531
S	-8.67880966847235	-2.11872730883293	0.49322518532638
S	-10.58868961830306	1.75975997179738	-3.15705155642363
S	-15.44546580162423	-3.64228834497066	0.58576342144344
S	-12.67924885579976	1.08394581606884	1.72613959164977
S	-13.13970057836936	-3.01660045726482	-2.64802185304683
S	-16.46835140473650	0.21002600038011	0.44967730996692
S	-14.91568333241426	-2.69948757680753	-5.00627102875168
C	-17.63177477577637	1.38221483397089	-0.39136969580347
C	-16.88053822293054	-4.09513929854069	1.66898194453928
C	-11.75957514908876	1.19327354667812	3.32874230727197
C	-12.28947779072344	2.46437937092541	-2.85046807561076
C	-9.82753450535150	-3.53293414849725	0.88449636146038
C	-13.12503780067928	-2.88188545427941	-4.49363779718703
C	-18.73990904078267	1.78670928403691	0.57976779589534
H	-18.31275055043486	2.32988289842626	1.44742053965076
H	-19.45697368746270	2.46748010420790	0.07616660208897
H	-19.29137500083138	0.90056659568637	0.94812566406181
C	-16.83869056681168	2.57843232755031	-0.92560288480979
H	-17.50869955636353	3.25963187341484	-1.49065252356048
H	-16.37938056048059	3.13653172173550	-0.08309136699183

H	-16.02464508769020	2.24028652220041	-1.59733833620838
C	-12.57314550340527	-4.17807530924554	-5.08982157204479
H	-12.59585205285318	-4.12749236746315	-6.19771813589158
H	-11.51607570310511	-4.31680597668560	-4.78359553705445
H	-13.16162431855095	-5.05479784480427	-4.75832357123561
C	-12.33391591386771	-1.64428939663168	-4.92163322934295
H	-11.27290444231376	-1.74291884661032	-4.61226378275462
H	-12.37799631032271	-1.52483412424568	-6.02446118610141
H	-12.74098013364102	-0.72826160487673	-4.44940946555506
C	-12.79362900767329	3.13775161601565	-4.12607705442416
H	-12.81679297655626	2.42640266706481	-4.97370401916277
H	-13.81561836514898	3.53840886194809	-3.96769106993108
H	-12.13760660327278	3.99111895209697	-4.39378990558357
C	-12.24524502412437	3.41292823360659	-1.65106149533692
H	-11.55633532494446	4.25832350908516	-1.85524012277646
H	-13.25809019646782	3.81479233967421	-1.44161493714462
H	-11.89316630426137	2.88224667626520	-0.74523012336732
C	-9.32442227282315	-4.25344255917340	2.13419764300232
H	-10.01143920816850	-5.08252901158672	2.39959730966267
H	-8.32471411419721	-4.69442255360669	1.94353611273419
H	-9.25173789747033	-3.56134259881063	2.99483830006459
C	-9.94689319259147	-4.45412108518794	-0.33082714261424
H	-8.95701669584629	-4.88745214867853	-0.58423422354339
H	-10.65933925396738	-5.27682598340771	-0.11657188398731
H	-10.32404721925615	-3.89574137460548	-1.20973324934878
C	-10.51877120073661	0.30031600945465	3.27728105785147
H	-10.78931193105756	-0.74497779280222	3.02842246671593
H	-9.82046981295450	0.65956131872581	2.49347209126231
H	-9.99977667875865	0.31275107061730	4.25855102934011
C	-11.43261238673636	2.65675655854060	3.62514126733141

H	-10.90889004284350	2.73901204113262	4.59956585316305
H	-10.75250496717696	3.05938136279707	2.84697179616514
H	-12.35011854822173	3.27467705390523	3.65832514906100
C	-17.20507649341259	-5.57742110912738	1.48330401277740
H	-18.04277131314141	-5.86922784029029	2.14933362020882
H	-17.52569947098887	-5.77139991008265	0.43915713330845
H	-16.32826585529540	-6.21213991120527	1.71391914280793
C	-18.06564623784670	-3.17936502343482	1.35873430843570
H	-17.78150073810306	-2.11225816317278	1.45123025637870
H	-18.41322665343495	-3.34398860760045	0.31739610125712
H	-18.90257270460586	-3.39129726042213	2.05621602687501
C	-14.95564877569192	-1.88278646898114	5.41358930681512
O	-15.54743500341821	-2.87439919774317	5.82484250721262
O	-14.53964674276480	-0.88571647030656	6.23389951187981
C	-17.72104061977788	-1.12745867186657	-4.39440545074992
O	-18.83101159294696	-0.63369642806299	-4.22933778217285
O	-17.36041177390106	-1.73842351747262	-5.55043895295394
C	18.52281038816336	-16.57264875026709	-9.94891750845833
O	17.84492921690671	-16.95444741908608	-9.00226865198044
O	19.68905696471362	-17.16671791911182	-10.30610691155365
C	17.61286078640682	-11.93513644223744	-19.03810147043229
O	17.54077083097878	-12.93804169104490	-19.73939995874234
O	17.87262339918048	-10.70588208442720	-19.54902472563901
C	18.06392821131530	-10.64042928603566	-20.96688906336503
H	18.25586450429722	-9.57775548565179	-21.19579075547557
H	18.92475562791527	-11.26698164844691	-21.27548802501301
H	17.16096412637435	-10.99787388138994	-21.50090882494748
C	20.13884730886222	-18.22911001696284	-9.45625088743466
H	21.10278138487951	-18.56359635836959	-9.87669898555434
H	20.27122856606269	-17.87071872385978	-8.41618004068071

H	19.40552317377760	-19.06012513528645	-9.45021629114163
C	-14.77130327716588	-1.08327934007982	7.63381874278080
H	-14.34041573780854	-0.20079505282026	8.13801267680812
H	-15.85680696817172	-1.16050400268557	7.84226227223381
H	-14.27956631575209	-2.01192653314185	7.98541749947946
C	-18.35861332809146	-1.78370826762896	-6.57700337768399
H	-17.89498752243172	-2.31865943101174	-7.42401317122061
H	-19.26108649684519	-2.31970358064471	-6.22193590507564
H	-18.65587030169016	-0.76015748529318	-6.87885844537079
C	2.56380879987949	0.34643231571767	-0.76331278160659
C	3.79425842734176	0.40492693115564	-0.78176020611775
C	5.21799652438154	0.47303957641119	-0.82532665691590
C	5.92223704281936	1.36364340534643	0.02039237533260
C	5.94043780432096	-0.34595392281636	-1.72645258495740
C	7.33474861319884	1.44937972289725	-0.03841772406395
H	5.36730359049191	2.00061675641314	0.72211263014666
C	7.35344136459290	-0.27758496903199	-1.79165731974153
H	5.39895238392606	-1.04134977600235	-2.38168585712843
C	8.04317138982187	0.62701131363648	-0.94843255175391
H	9.13916053059635	0.68834419100368	-0.99696584101024
C	8.06942699632414	-1.11746257379313	-2.69416948012529
C	8.67572594056286	-1.85555998943193	-3.47229019663073
C	9.36049911327727	-2.72145853504105	-4.37028779751435
C	8.68578622426195	-3.81360107629619	-4.97859421202851
C	10.73164501903775	-2.52344859135046	-4.68222378079060
C	9.35737536650692	-4.66600613133744	-5.85657740134906
H	7.62735767054840	-3.98842160946507	-4.73719683606982
C	11.39239919413160	-3.38136941605619	-5.56429341227074
H	11.26550282159474	-1.67518323703291	-4.22996346210260
C	10.72452586209469	-4.47247536501169	-6.17253649411547

H	8.81915246834660	-5.52309040521123	-6.28812189317060
H	12.44628705298808	-3.18610695725822	-5.81307088723787
C	11.42808108068256	-5.38202160266686	-7.10638789060330
C	12.80291557444874	-5.68063353374348	-6.94653011129634
C	10.74757005619800	-5.98629254605076	-8.18890454317562
C	13.46279948680472	-6.54343650744653	-7.82592039267649
H	13.35883038052892	-5.25249731297381	-6.09913281564371
C	11.38937813675124	-6.85497675381248	-9.08139366320610
H	9.68689236115006	-5.74945763595499	-8.36050586051343
C	12.75610795981694	-7.13033907552219	-8.89168331870123
H	14.52592867757056	-6.79041250314494	-7.69815874660707
H	10.84985031540054	-7.29358672758764	-9.92679797052812
C	8.03735107382259	2.35682627461483	0.80725159952969
C	8.64546818589208	3.14299365052427	1.53525335823610
C	9.35047460629891	4.05305322985545	2.37185006346649
C	10.73294420682497	4.30797743922768	2.16933256433070
C	8.69178674854375	4.73271612904380	3.43108784538685
C	11.41972141974592	5.20339586250369	2.99168977640337
H	11.25480764727866	3.80051013738951	1.34520718315513
C	9.38933653641979	5.62480111712944	4.24822900049535
H	7.62442328221181	4.53678465111792	3.60901737491348
C	10.76776876610023	5.88275681680738	4.04998873442686
H	12.48278842229640	5.40677967851530	2.79318824458752
H	8.86227869536772	6.11190760768871	5.08225101130887
C	11.50384059778087	6.82866962915549	4.92079676563423
C	12.86551954525148	6.61530017248541	5.24407715893595
C	10.87072361891171	7.97473682347893	5.45654760905414
C	13.55946220454943	7.50188034415024	6.07268776768780
H	13.38063127419695	5.71992233079247	4.86577388017773
C	11.55055313755490	8.87747087762973	6.28444752783793

H	9.82295659163482	8.18506002628123	5.19544334486950
C	12.89957932460503	8.62884202793735	6.59212623057526
H	14.61202457878658	7.33464991329645	6.34148799542681
H	11.04411840397387	9.76069421137870	6.68900931707734
O	13.68865181128643	9.47417712658627	7.35714263260618
C	13.22042140331092	10.19000794091447	8.43150600035943
O	12.05735841326120	10.16286397206792	8.80553066199013
C	14.30359903830117	10.94220931988660	9.09005984604522
C	15.67056728300984	10.88108405894653	8.68805232672846
C	13.95265981070301	11.70472408810016	10.24628524628266
C	16.65507010066985	11.52222870105054	9.47525595493762
S	16.28885044601373	10.01924855867774	7.27015372676073
C	14.94891627402459	12.33912401804005	11.01955489633594
S	12.28218217391089	11.94329173181247	10.78516435882563
C	16.31557938548703	12.29028804462952	10.62156174263974
S	18.34155189392113	11.18142916274905	9.06883965210736
C	17.97206383094118	10.83780139859149	7.28831517172858
S	14.39253758506293	13.22555623268428	12.44724012113194
C	12.68461262827449	13.47518625176074	11.77909588897578
C	17.33336409008082	13.08252447606229	11.30315979199427
C	19.00139181721997	9.85000271004964	6.73836075419191
C	17.91826741265213	12.14827135728457	6.50001652227271
C	12.66925989377804	14.70013665722324	10.86056902063605
C	11.69816367718910	13.59071625263249	12.94014674470010
C	18.24033764666482	13.90256254651976	10.51418667422657
C	17.31157522561311	13.19774331267418	12.75036944459868
H	18.76239737180154	9.59710452612667	5.68526976787656
H	20.01084635507508	10.30938705603913	6.74902917239183
H	19.02263520978724	8.91822173151249	7.33439425093170
H	17.18889344445472	12.85471196299151	6.94459914746358

H	18.91350705629143	12.63851411596096	6.50805402707791
H	17.62475807153558	11.94872629329745	5.44823574774335
H	11.67699782950283	14.80269142785098	10.37431001025191
H	12.87695200803024	15.61903293487376	11.44783894471407
H	13.44710237954568	14.61244982461322	10.07615945727790
H	11.92971028659673	14.48712758011908	13.55159621249016
H	10.66497678954554	13.70780955585943	12.55315692761109
H	11.73436602520708	12.69540730973128	13.59013443259849
C	17.78603124486333	14.62972368435129	9.37913210707133
C	19.60972455481432	14.00673704672731	10.88290754450950
C	17.37785251575412	14.46947820175581	13.39862531016708
C	17.08811252547197	12.04296425301634	13.55445984056302
C	18.66301863919233	15.48095374904993	8.67174234952949
S	16.11372979321412	14.57427841410008	8.79602263430406
C	20.51269655175351	14.79320263010426	10.13222325459248
S	20.27886814927964	13.10631045457560	12.25337008348173
C	17.09213664959059	14.57922166994628	14.77946110710027
S	17.71705979433019	15.97821869390850	12.54207209486972
C	16.81985876393756	12.13812150354664	14.93816102005217
S	17.28814572149793	10.42181085248413	12.88807516560110
C	20.03755281869903	15.56019045243609	9.03155259724961
S	17.94565608251445	16.41117302514550	7.34626581898950
C	16.20855511855959	16.25296261936256	8.01598064625052
S	22.20298014942791	14.71118813377850	10.66018194887844
C	21.99276774713826	13.08001526392926	11.54102115647575
C	16.83658151231214	13.41659352569775	15.56670013763265
S	17.03556561313140	16.22349602617941	15.43434409225571
C	16.79567283268044	17.02344801362662	13.75970883712399
S	16.52472780115251	10.59385298955331	15.75275123842806
C	16.22641376814784	9.62488739000344	14.17956820109912

C	20.90864598248660	16.42739061662487	8.21076564062163
C	15.21687690171262	16.32802327817481	6.85681551890304
C	15.96299953905190	17.32101358980855	9.08484873910573
C	23.01126297649745	12.99167703836548	12.67617972504082
C	22.11505720137825	11.93289879390588	10.53587775900007
C	16.67269028713931	13.60984233040312	17.02384568370901
C	15.30605661552905	17.02341655181935	13.40657438470586
C	17.40484466966593	18.42476066421262	13.78357883739891
C	14.75244608325890	9.72440139306012	13.78292275815507
C	16.68980014992149	8.18325574275730	14.39473575612779
O	20.60621920097738	16.87465487322326	7.11069159059519
O	22.09315383593531	16.69840262417164	8.81492318887587
H	14.17937780075744	16.20301906313984	7.22986810989271
H	15.27811278081687	17.32116624873034	6.36588155860673
H	15.42258925834397	15.54556428178940	6.10111749641123
H	16.10440847840058	18.33498233237159	8.65595158819328
H	14.92792995961162	17.23726677078187	9.47662891982229
H	16.66094141934762	17.18983770067032	9.93547701919523
H	22.88724494972618	12.03557135435793	13.22522883832971
H	24.04297142901403	13.01154402947451	12.26848701098803
H	22.89020175551718	13.83077375216151	13.38819369021602
H	23.12091992694881	11.93851304618908	10.06657503406971
H	21.96022972909373	10.95959134867127	11.04614645897066
H	21.35204384354272	12.02852379092932	9.73770901708235
O	16.77071819079508	14.68842207374280	17.59827736604817
O	16.42875943038213	12.45160963959884	17.68591801795289
H	14.73059443801556	17.59587444542152	14.16329755146238
H	15.14980836822315	17.48496922081557	12.40874151068644
H	14.91469841201084	15.98741947861793	13.36744116644829
H	17.28573101258014	18.91373652457334	12.79433865505496

H	16.88313727773196	19.05395276652813	14.53327324674890
H	18.48140791206691	18.38542429991884	14.03661497224439
H	14.57536839360444	9.17939064636806	12.83306786716037
H	14.11352702867661	9.28361628121218	14.57617186709543
H	14.44879786530178	10.77844556094324	13.62799847773470
H	16.09670260902861	7.70903552932285	15.20314525826765
H	16.52817681944891	7.59054056637372	13.47103197807746
H	17.76190607183543	8.14276709422244	14.66623099661986
C	23.02377154340376	17.47512150428604	8.05028054934126
C	16.29350022618249	12.55475378603866	19.10790034496326
H	23.92447415683498	17.57319601503877	8.68060179129283
H	23.26891679848242	16.96649121937861	7.09686999144673
H	22.59968025371273	18.47142716823327	7.81656329625377
H	16.12334509902177	11.52585495243790	19.46970444282904
H	17.21180331152323	12.98109058995600	19.55884417949938
H	15.43729472445762	13.20664826305321	19.37367836456018

4a$^{\bullet\bullet}$

224 atoms

C	-5.23062816388911	-0.04235766603479	0.25234972020021
C	-3.83279050026582	-0.02067167710098	0.23089978296267
C	-3.12423949337408	0.53089384450772	-0.86318221043726
C	-3.87881891014106	1.06888127875475	-1.93140162232393
C	-5.28054847697136	1.05950046374248	-1.92733490936486
C	-5.95052235536604	0.49479682459541	-0.82829974559667
H	-5.78663648368304	-0.48632496590154	1.08987995993307
H	-3.27720478092863	-0.47241481185490	1.06592544081671
H	-3.35642688819962	1.53040234116960	-2.78263127881625
H	-5.84816729964477	1.47924582009766	-2.76512418144229
C	-1.64146910730892	0.53478348474822	-0.88680871480368

C	-0.92584970099376	0.38526902366586	-2.09598184876121
C	-0.89089103122688	0.67547191494323	0.30533035828336
C	0.47588913282575	0.36982761118418	-2.13909596647331
H	-1.47944100481693	0.24374172522037	-3.03647127851687
C	0.50540412848112	0.65803808926465	0.28778271981936
H	-1.41238093057778	0.82577540592029	1.26241778744211
C	1.18923556564121	0.50383269650503	-0.93314669360794
H	1.01324393579741	0.23328658225630	-3.08263896549382
H	1.09307115710350	0.76904408917190	1.20979012299678
O	-7.33458065597054	0.46960184516440	-0.69501293084275
O	2.57127501450591	0.51180358113842	-0.80514126852690
C	-8.18121485357841	0.21223491497661	-1.74537665736721
C	3.47689868960044	0.22704467529805	-1.78814319001624
O	3.17689309968861	-0.04535697541037	-2.94307614420504
O	-7.82098361246413	0.14816019462285	-2.91088580418070
C	4.86475229142564	0.30658382490466	-1.28852387397216
C	5.22140137085300	0.78156945590690	0.00773066216193
C	5.90153663609099	-0.06735696959904	-2.19920980299465
C	6.58751899188362	0.91170641603826	0.34682241499341
C	7.26151582263731	0.05373860185724	-1.83567445676173
C	7.62497719928110	0.49392945158102	-0.53068085569684
C	-9.57359665204727	0.04381184896975	-1.28880722087782
C	-9.93984762593870	-0.21837640354841	0.06385339516770
C	-10.59485947478499	0.10795132929898	-2.27977135805505
C	-11.29932468800640	-0.38688272755457	0.40565266794601
C	-11.94556225274875	-0.10989008289458	-1.93071782817164
C	-12.31883345654136	-0.35259480181862	-0.58250241807889
C	-13.71695901746505	-0.54905217228792	-0.21079266892999
C	8.99864417075194	0.41029264853248	-0.04717718016048
C	9.24846998347897	-0.20209659626769	1.25069523212125

C	8.54220075532944	-1.36283255195171	1.67086704031414
C	10.21644771568732	0.35718919224813	2.12853247748883
C	8.82320654745768	-1.96584292787083	2.91605897984829
C	10.45067180367385	-0.19559957123861	3.40818518628397
C	9.76785058684630	-1.37974881545817	3.80404327709231
C	10.10539555088798	0.74876142912572	-0.92248851808978
C	11.21415638226050	-0.13536699609280	-1.09746137293952
C	10.04683232510033	1.92355179806355	-1.72610143090647
C	12.14139398647691	0.08678968549890	-2.14263510440704
C	10.98001948087266	2.16824319340057	-2.75829493732615
C	12.04982138851267	1.24935983924551	-2.96546662266533
C	-14.57802275471846	-1.33769942727694	-1.08042534526783
C	-14.11055153687784	-2.55172566654111	-1.65697039446917
C	-15.87454784543244	-0.87381683924528	-1.44528775777776
C	-14.85135540482254	-3.23693024683404	-2.64873875815457
C	-16.62799219467428	-1.54969051263819	-2.42928269940643
C	-16.13506103644678	-2.74896997260613	-3.02639042649521
C	-14.25330084141854	0.11509079632404	0.96763649099870
C	-15.11663919542600	-0.58089166675766	1.86082935642598
C	-13.93149223318190	1.47225397475786	1.26642445659019
C	-15.66696772112485	0.06014154999030	2.99347406726970
C	-14.40440937487402	2.09766057989748	2.44279077310318
C	-15.29846436532951	1.39970735561665	3.30687180358689
S	13.36445803669271	-1.17218981250890	-2.37715811675693
S	11.59378724952590	0.70474678433956	4.42085901294196
S	7.93657364435914	-3.45619434945434	3.27787880700324
S	10.71242762240208	3.66169905441847	-3.67204281519648
S	8.45140115292270	-0.42413578362311	-3.05593374093277
S	6.95826541549486	1.76103896655359	1.85179179676243
S	-11.66330542326087	-0.68872510761411	2.11551670567825

S	-13.11918846017758	-0.03302382700413	-3.25908984896016
S	-18.17143781727170	-0.80183643828420	-2.87208403394020
S	-16.73036872477250	-0.92698910342024	4.00093203294334
S	-13.86224558997520	3.76816934206955	2.68046733634683
S	4.09082741367925	1.29992034708639	1.26993819820821
S	5.58709651716312	-0.73920552878094	-3.80785651467314
S	8.89411935484317	3.20853304050019	-1.36321057500468
S	11.45840305972566	-1.58966186462672	-0.12246162052557
S	11.12158158560068	1.82129847216689	1.71084292297221
S	7.30664976595528	-2.16020907145825	0.68141049701649
C	11.37485146657878	2.30380229282232	3.48491506894323
C	7.56980189271557	-3.81178917730427	1.48133247633041
C	12.38935661819302	-2.47524667617828	-1.45461152067966
C	8.95986560385019	3.92657528524636	-3.06993088439889
C	7.27367614886892	-1.53089253629432	-3.95784656184450
C	5.35389803931539	1.30282888024402	2.64965989190178
C	7.98017281348549	3.18872707152802	-3.98386154562721
H	6.94041494531212	3.33668431727280	-3.62402677227234
H	8.05900195198162	3.57840627251719	-5.02039922775936
H	8.18509504899133	2.09968308793806	-3.99432889728993
C	8.68919900577074	5.42979005864431	-2.99479190727356
H	8.76223008449458	5.88308887313401	-4.00445648280604
H	7.65987513765771	5.61125769388113	-2.62332233728220
H	9.40871050279663	5.93527186013092	-2.32305419528725
C	7.25489787934797	-2.90782882115742	-3.28845798401567
H	6.49205043139677	-3.55527507632525	-3.76852174789409
H	8.24923516233631	-3.39323444965204	-3.38099338393309
H	7.02034853506039	-2.81291980074419	-2.20988920179981
C	7.66502146299922	-1.59111666104510	-5.43413099429764
H	8.67438388030211	-2.03766492371714	-5.54595554958390

H	6.95159753172788	-2.23181451873332	-5.99141211051825
H	7.66868936850599	-0.58318772159062	-5.89104405414090
C	11.39458380439093	-3.13719760616494	-2.41110395832218
H	11.93133664394228	-3.61262489251538	-3.25825974231446
H	10.80529146473242	-3.91057848476014	-1.87550281354756
H	10.68528161046319	-2.38655998250531	-2.81374779540738
C	13.36119412901740	-3.46759090463276	-0.81830630118975
H	12.80556156091792	-4.24421789309893	-0.25304378095850
H	13.94910011155055	-3.98363846483221	-1.60507433005413
H	14.06052516702904	-2.95511008748655	-0.13009354991027
C	12.65355237852610	3.13153641457314	3.60770218651982
H	12.55176642747283	4.07912530387839	3.04093823301191
H	12.83935324997364	3.39403071698773	4.66912385014785
H	13.52662964211407	2.57448307384851	3.21815277405006
C	10.13330820265790	3.03490554395033	4.00019860356376
H	10.25344095249656	3.28742264410726	5.07414577064619
H	9.97322891915313	3.96895628529651	3.42352217312183
H	9.23180955035473	2.40076514030038	3.88377922445697
C	6.28336967735634	-4.63066612110522	1.38493334542258
H	6.03599327456484	-4.83369146458119	0.32290137239140
H	6.41426514935807	-5.60900968982166	1.89062912454313
H	5.43346673157855	-4.09929922390002	1.85365968896825
C	8.77042281016925	-4.50510933236228	0.83202954126047
H	8.98650158351544	-5.46411913344380	1.34669407345561
H	8.55869494471974	-4.70854055972617	-0.23814561333118
H	9.66947970383923	-3.85937985844504	0.88579695092422
C	4.96623510189631	2.37764610608548	3.66546381773335
H	3.98253025807792	2.13690459032991	4.11783865274248
H	5.71050641703190	2.41231058168335	4.48735558701119
H	4.90693834683241	3.37574006306417	3.19132866283794

C	5.45381783891546	-0.09563794447123	3.26188862119815
H	5.74422331996607	-0.84683376098822	2.50099144770966
H	6.22097281830272	-0.10877201573966	4.06378116985085
H	4.47598440678837	-0.39294259040379	3.69454390229226
S	-8.80421393508415	-0.37323822087401	1.42021302503410
S	-10.28026944813923	0.45569800706985	-3.98795129269052
S	-15.40334690240507	-2.31550251685743	1.71969203240355
S	-13.08304338095129	2.51272230982789	0.11466609424663
S	-12.66980072616826	-3.36912369000941	-1.04099275927357
S	-16.59189670916975	0.58706606529404	-0.75506097749901
S	-14.08623148080020	-4.70219635611398	-3.28436678127169
C	-17.69020216477346	0.88316705968588	-2.21775853152123
C	-16.97754363452547	-2.25299340875586	2.69744204415278
C	-12.47671307863810	3.66681813718485	1.42970143824260
C	-12.02686989354314	1.02954147516710	-4.31592218609505
C	-9.97494677827094	-1.36910182153273	2.47073893013710
C	-12.38561447662351	-4.32634106355344	-2.59872355885916
C	-18.94997035894843	1.62218714169899	-1.76763700334226
H	-18.68499899566130	2.61849130780155	-1.35857201716976
H	-19.62550695315542	1.78370080508475	-2.63255559917623
H	-19.49394531200094	1.04951618867387	-0.99241214371038
C	-16.90460740699987	1.64245082469978	-3.29131717285210
H	-17.52826045484645	1.77518636957014	-4.19955341582902
H	-16.60278603192080	2.63999124220088	-2.90989228215606
H	-15.98568256018677	1.08920705296023	-3.56936313555162
C	-11.69069392400878	-5.64655539391788	-2.26101720173901
H	-11.55195695231669	-6.24837236330142	-3.18202430727394
H	-10.68450668362560	-5.44950201723345	-1.83796943823585
H	-12.27989857726116	-6.23565499693715	-1.53257673946867
C	-11.59743788783943	-3.47634825590726	-3.59723165912484

H	-10.58962773934741	-3.24746455176695	-3.19379044206983
H	-11.48335295796889	-4.02315651126966	-4.55613221803382
H	-12.10851387479085	-2.51358482731956	-3.79504798344980
C	-12.36606569107772	0.78887497737704	-5.78630703355496
H	-12.25622537406589	-0.27959173528377	-6.05277883184568
H	-13.40883812156337	1.10460666011312	-5.99491620597040
H	-11.69822560598029	1.38937990499017	-6.43664086323762
C	-12.17719438367830	2.49466386373852	-3.90011166009641
H	-11.49430866755216	3.13589260454634	-4.49461008380806
H	-13.22230911619673	2.83111784934365	-4.06283056239768
H	-11.93803284444278	2.62494488446790	-2.82628795870888
C	-9.63642283526200	-1.14438985022332	3.94383541332544
H	-10.34660538234676	-1.70141012528789	4.58784512755786
H	-8.61747998753193	-1.52360894130621	4.16351899759175
H	-9.68329350197923	-0.07041127738581	4.20629251567926
C	-9.91595342889771	-2.84222065022512	2.06130685928526
H	-8.89328589516288	-3.24390304393043	2.21915270152926
H	-10.63498335543354	-3.43418407549080	2.66364403353945
H	-10.18272948477289	-2.96373892876843	0.99314258366254
C	-11.21742518810167	3.09493910338529	2.08341052066584
H	-11.39765122488942	2.07126656890219	2.46695626968750
H	-10.39482313104186	3.03380551538327	1.34057118558644
H	-10.89660529944060	3.74265356533915	2.92581573628085
C	-12.25819254348577	5.05335280054993	0.82386417616876
H	-11.93630148081372	5.76595369736609	1.61044524125809
H	-11.45379456552791	5.01009457697448	0.06079247760405
H	-13.18311380504464	5.43620473508099	0.35205022272720
C	-17.18994119674775	-3.59354353797689	3.40094544964162
H	-18.11490641057198	-3.55752550283806	4.01198875687308
H	-17.31426468375124	-4.40312785535012	2.65292088124676

H	-16.33638757288812	-3.84049887850870	4.06091957651999
C	-18.13843041937361	-1.87014703572370	1.77745447786487
H	-17.92852030691118	-0.92341861155975	1.24062409504060
H	-18.30057111836047	-2.65951653298342	1.01367138761266
H	-19.06845111981318	-1.74814940644802	2.37126626271208
C	-15.86320083672216	1.98521553835815	4.54112295267277
O	-16.46299877264515	1.34913859385251	5.40004575199594
O	-15.66172122753251	3.32410848011322	4.62762686942741
C	-17.03854857279822	-3.46053871093509	-3.95564088947282
O	-18.19118746594415	-3.12152015374463	-4.20019930969180
O	-16.47308091233564	-4.56644727335578	-4.50086223276033
C	9.96679096104239	-2.03043512718459	5.11586637074576
O	9.20853295775619	-2.86185385340616	5.60123886872830
O	11.10224840838206	-1.61982071273516	5.73489403763331
C	13.12947242599209	1.46584170341685	-3.95279659248056
O	14.10571093401495	0.73619542734978	-4.08763637695330
O	12.95227610274153	2.58947461969275	-4.69136745229908
C	13.97639135474259	2.88226825443397	-5.64926554839290
H	13.67808513593735	3.83185929709005	-6.12658539421527
H	14.96031699841722	2.98737044376573	-5.15096836285545
H	14.05035316547394	2.07307324314231	-6.40306669570251
C	11.32919317869583	-2.15163111114796	7.04606898415564
H	12.26879523880161	-1.69237834832308	7.39878984681935
H	10.49073262693791	-1.89538760036055	7.72438831430869
H	11.42496373352332	-3.25508487920038	7.00730168210628
C	-16.13748638877389	3.95698645709714	5.82211863229063
H	-15.83119289297277	5.01470439452042	5.74466567863297
H	-17.24046231761082	3.87395380061340	5.89426104927955
H	-15.69069653710458	3.48612500783775	6.71980927051105
C	-17.30757362966063	-5.33341201618188	-5.37742425253363

H	-16.70221276214399	-6.20384039636718	-5.68424222315272
H	-18.22778490868200	-5.66094276993598	-4.85440342629780
H	-17.60206496904626	-4.73343572576146	-6.26115939300873

5. References

1] Valera, S.; Taylor, J.E.; Daniels, D.S.B.; Dawson, D.M.; Athukorala Arachchige, K.S.; Ashbrook, S.E.; Slawin, A.M.Z.; Bode, B.E. A Modular Approach for the Synthesis of Nanometer-Sized Polynitroxide Multi-Spin Systems. *J. Org. Chem.* **2014**, *79* (17), 8313–8323. DOI:10.1021/jo5015678. Available Online: https://pubs.acs.org/doi/pdf/10.1021/jo5015678 (accessed on 16.02.2018).

2] Saigo, K.; Usui, M.; Kikuchi, K.; Shimada, E.; Mukaiyama, T. New Method for the Preparation of Carboxylic Esters. *BCSJ* **1977**, *50* (7), 1863–1866. 10.1246/bcsj.50.1863. Available Online: http://www.journal.csj.jp/doi/pdf/10.1246/bcsj.50.1863 (accessed on 16.02.2018).

3] Liu, Y.; Villamena, F.A.; Sun, J.; Xu, Y; Dhimitruka, I.; Zweier, J.L. Synthesis and Characterization of Ester-Derivatized Tetrathiatriarylmethyl Radicals as intracellular Oxygen Probes. *J. Org. Chem.* **2008**, *73* (4), 1490–1497. DOI:10.1021/jo7022747. Available Online: https://pubs.acs.org/doi/pdf/10.1021/jo7022747 (accessed on 16.02.2018).

4] McConnell, H.-M. *J. Chem. Phys.* **1956**, *24* (2), 460–467.

5] Rajca, A. *Chem. Rev.* **1994**, *94* (4), 871–893.

6] Bode, B. E.; Margraf, D.; Plackmeyer, J.; Dürner, G.; Prisner, T. F.; Schiemann, O. Counting the Monomers in Nanometer-Sized

Oligomers by Pulsed Electron− Electron Double Resonance. *J. Am. Chem. Soc.* **2007**, *129* (21), 6736–6745.

7] Jeschke, G.; Sajid, M.; Schulte, M.; Godt, A. Three-Spin Correlations in Double Electron–electron Resonance. *Phys. Chem. Chem. Phys.* **2009**, *11* (31), 6580–6591.

8] von Hagens, T.; Polyhach, Y.; Sajid, M.; Godt, A.; Jeschke, G. Suppression of Ghost Distances in Multiple-Spin Double Electron–electron Resonance. *Phys. Chem. Chem. Phys.* **2013**, *15* (16), 5854–5866.
